# A review on synthesis of furonaphthoquinones through lawsone derivatives annulation reactions and their biological properties

**DOI:** 10.1039/d4ra08843c

**Published:** 2025-02-04

**Authors:** Abolfazl Olyaei, Mahdieh Sadeghpour, Seyede Bita Sajjadi

**Affiliations:** a Department of Chemistry, Faculty of Science, Imam Khomeini International University Qazvin Iran; b Department of Chemistry, Qazvin Branch, Islamic Azad University Qazvin Iran mahdieh.sadeghpour@qiau.ac.ir

## Abstract

Furonaphthoquinones and their dihydro derivatives have attracted significant attention due to their diverse pharmacological activities. These compounds can be derived from natural sources, including various plants, or synthesized through chemical methods, resulting in a wide variety of structures with distinct biological properties. As a result, numerous methods have been developed over the past decades for the preparation of these compounds, particularly utilizing 2-hydroxy-1,4-naphthoquinone derivatives as key precursors. Considering these concepts, this review aims to offer a comprehensive overview of the chemical synthesis of linear and angular furonaphthoquinones, along with their dihydro derivatives derived from 2-hydroxy-1,4-naphthoquinones annulation reactions, and to explore their diverse biological activities.

## Abstract 2

Furonaphthoquinones and their dihydro derivatives have attracted significant attention due to their diverse pharmacological activities.

## Graphical abstract

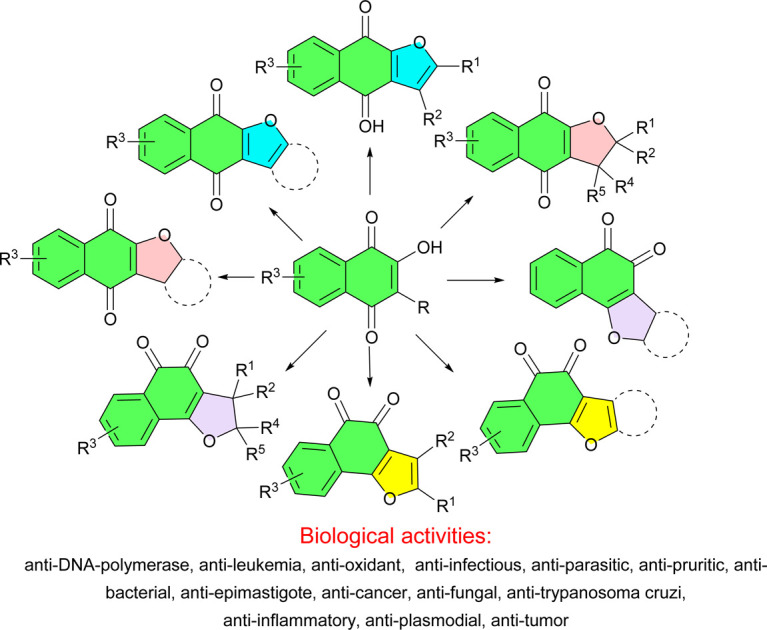

## 1. Introduction

**Fig. 1:**
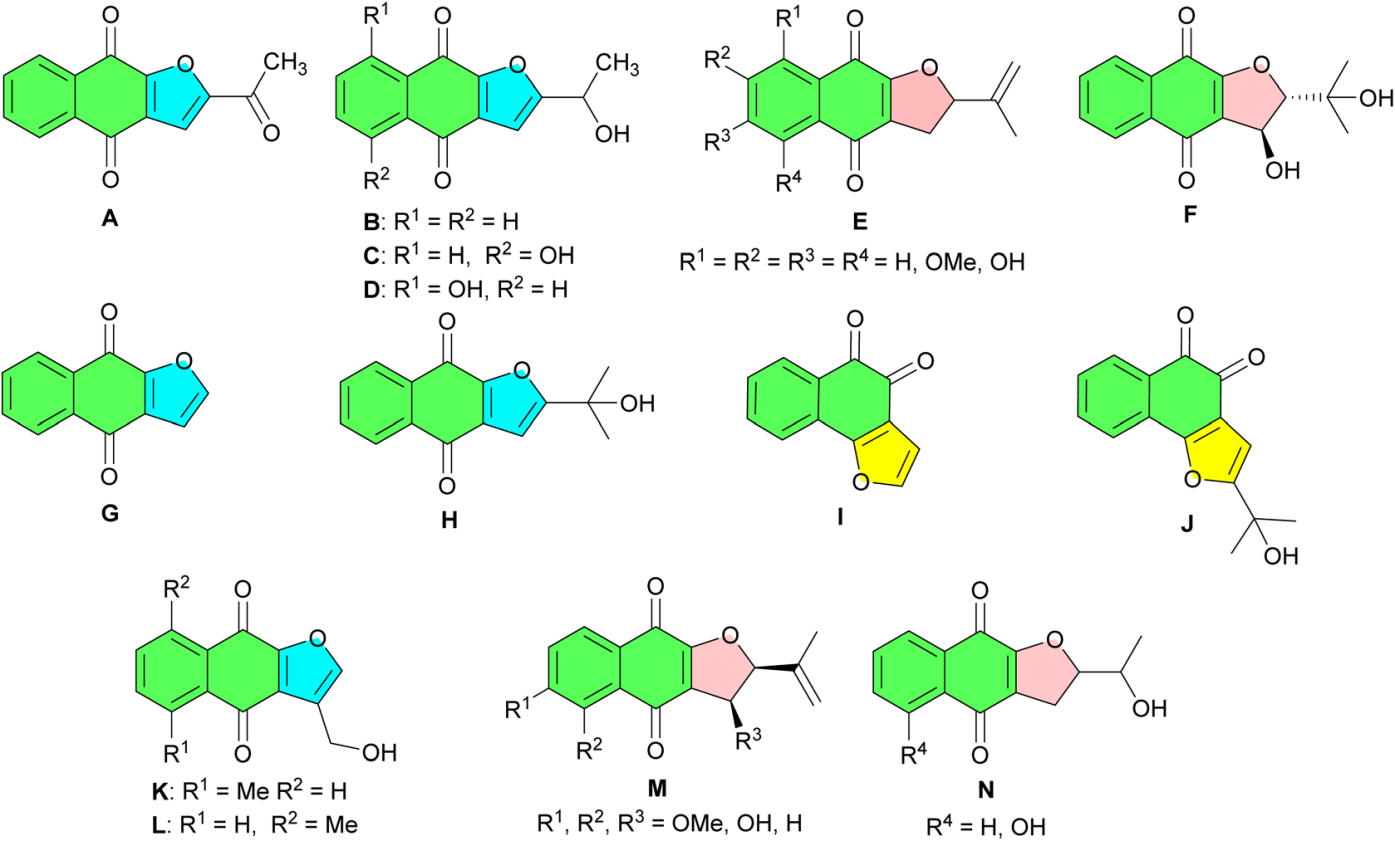
Some natural, bioactive furonaphthoquinones and their dihydro derivatives.

2-Hydroxy-1,4-naphthoquinone, commonly referred to as lawsone or hennotannic acid, is a naturally occurring naphthoquinone compound that has attracted considerable attention in the field of synthetic organic chemistry. Among the simplest naturally occurring naphthoquinones, it is best known for its presence in the leaves of the henna plant (*Lawsonia inermis*), where it contributes to the characteristic red-orange coloration.^1,2^ Henna extracts containing lawsone have been used for thousands of years as hair, nails, wool, cotton and skin dyes, highlighting its historical and cultural significance.^3–6^ In addition to its well-known dyeing capabilities, 2-hydroxy-1,4-naphthoquinone demonstrates a variety of biological activities, including anti-inflammatory, antibacterial, antiviral, antifungal, and antineoplastic effects.^7,8^ In organic synthesis, lawsone has been extensively utilized in various reactions, playing a pivotal role in constructing diverse molecular frameworks such as furonaphthoquinones and their dihydro derivatives. They constitute an important group of oxygen heterocycles. Many members of this group are widely distributed in nature and isolated from various plants.^9^ For example, as indicated in Fig. 1, furonaphthoquinones A–C isolated from *Tabebuia cassinoides*, exhibit anticancer activity.^10^ Kigelinone D, isolated from the fruits of *Kigelia pinnata*, exhibits antibacterial, antifungal, and antiviral activities.^11^ Compounds E were isolated from *Cresentia cujete*, and showed interesting mutagenic activities.^12^ Avicequinones F–H have been isolated from the stem bark of *Avicennia alba*, while naphthoquinones I and J have been obtained from *Avicennia marina*.^13^ Maturone K and Maturinone L, isolated from *Cacalia decomposita*, are derived from its root extract, which has been traditionally used for the treatment of diabetes.^14^ Naphthofuroquinones M and N, isolated from the dried wood chips of *Crescentia cujete*, exhibit moderate but selectivity against the repair-deficient rad52 yeast strain, suggesting their role as DNA-damaging agents.^12^

Furonaphthoquinones exhibit a diverse biological activities, including antitumor effects,^15^ antiviral activity against the Japanese encephalitis virus^16^ and Vero cells,^11^ and inhibition of human keratinocyte hyperproliferation.^17^ They also demonstrate antibacterial,^18^ anti-inflammatory,^19^ antiallergic,^20^ antiapoptotic,^21^ and antileishmanial properties^22^ along with inhibitory activity against receptor tyrosine kinases.^23^ Furthermore, they function as antipsoriatic^24^ and antileukemic agents^10^ as well as antiprotozoan activity against *Trypanosoma cruzi*.^25^ Additionally, these compounds possess antifungal properties^26^ and other cytotoxic effects.^27^ Due to their significant role in pharmaceutical research and drug discovery, substantial efforts have been directed toward developing synthetic strategies for furonaphthoquinone ring systems. Considering the importance of furonaphthoquinone derivatives and as part of our ongoing research into synthesizing organic compounds from lawsone and review articles,^28^ this review highlights the key synthetic methodologies used for preparing linear and angular furonaphthoquinone derivatives derived from 2-hydroxy-1,4-naphthoquinones. Emphasis is placed on advancements in reaction design, catalyst development, reaction mechanisms, and the discovery of new reaction pathways. Additionally, we explore the applications of these synthesized compounds in medicinal chemistry, highlighting their promising potential in drug development.

## 2. Synthesis of furonaphthoquinones

**Scheme 1:**
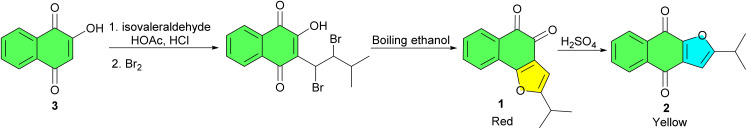
Synthesis of naphthofuroquinones 1–2.

In 1896, Hooker reported the first synthesis of naphthofuroquinone. 2-Iso-propylnaphtho[1,2-*b*]furan-4,5-dione (1) and 2-iso-propylnaphtho[2,3-*b*]furan-4,9-dione (2) were synthesized from 2-hydroxy-1,4-naphthoquinone (Lawsone) (3) through a multi-step process, as outlined in Scheme 1.^29^

**Scheme 2:**
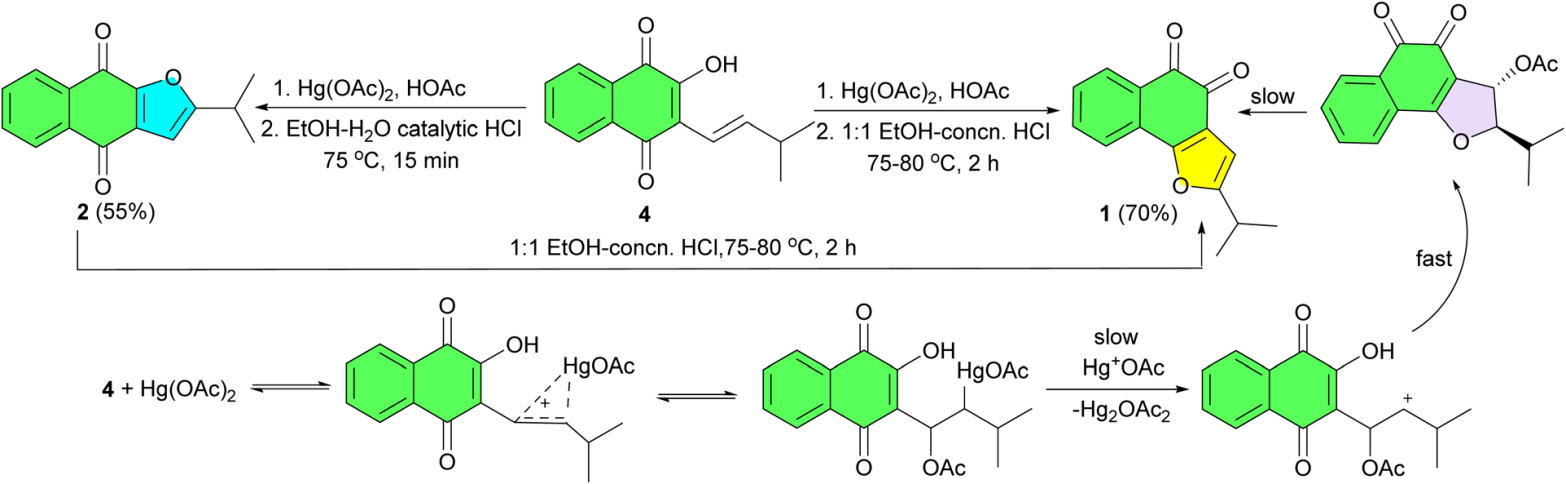
Hg(OAc)_2_ oxidation of iso-lapachol (4) to furanonaphthoquinones 1–2.

In 1967, Dudley and Waynemill reported that the mercuric acetate oxidation of iso-lapachol (4) proved to be an efficient synthetic method, yielding angular furanonaphthoquinone 1 with a 70% yield and linear furanonaphthoquinone 2 with a 55% yield. As seen in Scheme 2, the only differences in the experimental procedures involved the time of heating and amount of mineral acid employed in the second step. A plausible mechanism for the preparation of 1 is shown in Scheme 2.^30^

**Scheme 3:**
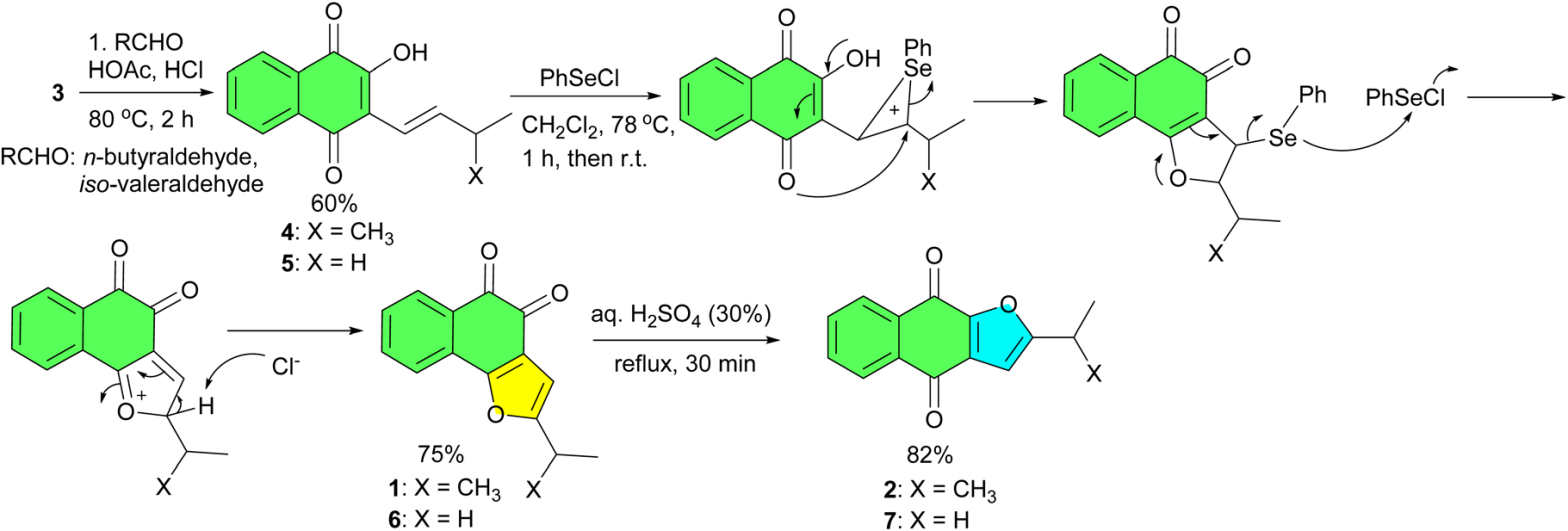
Selenium reagent in the synthesis of furanonaphthoquinones 1–2, 6–7.

In 1990, phenylselenoetherification was used to synthesize furanonaphthoquinones from lawsone. When derivatives 4 and 5 prepared from 3 according to Hooker's procedure, was treated under the same condition, the reaction was incomplete and requires 2 equivalents of benzeneselenyl chloride to give to completion, giving naphtho[1,2-*b*]furan-4,5-diones 1 or 6 in 75% yield, and dibenzenediselenide. The orthoquinones 1 and 6 could be isomerized with acid to lead to the corresponding naphtho[2,3-*b*]furan-4,9-diones 2 or 7 in 82% yield. In the proposed mechanism, the intermediate selenide was not isolated. A nucleophilic displacement reaction at Se(ii) with the participation of the oxygen atom of the dihydrofuran ring may be invoked to explain this process (Scheme 3).^31^

**Scheme 4:**
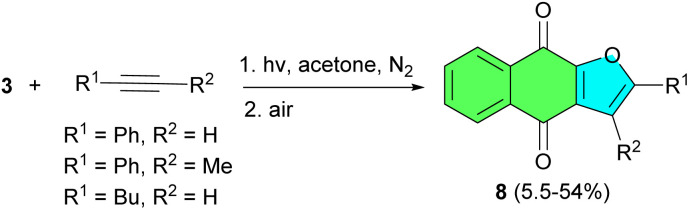
Regioselective synthesis of naphtho[2,3-*b*]furan-4,9-diones 8.

Next, the Suginome group demonstrated naphtho[2,3-*b*]lfuran-4,9-diones 158 can directly and exclusively be formed by an unprecedented regioselective [3 + 2] photoaddition of 3 with various alkynes in acetone for 7.5–30 h in 5.5–54% yields (Scheme 4). The photoaddition of hydroxynaphthoquinone with alkynes leading to 8 may proceed in a manner parallel to the photoaddition of alkenes and may also involve vinyl cations.^32^

**Scheme 5:**
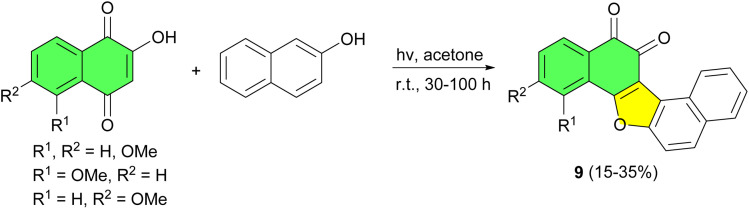
One-step synthesis of furanonaphthoquinones 9.

In 1995, one-step synthesis of furanonaphthoquinones 9 reported in 15–35% yields by a [3 + 2] photoaddition of 2-hydroxy-l,4-naphthoquinones with 2-naphthol in acetone at room temperature for 30–100 h (Scheme 5).^33^

**Scheme 6:**
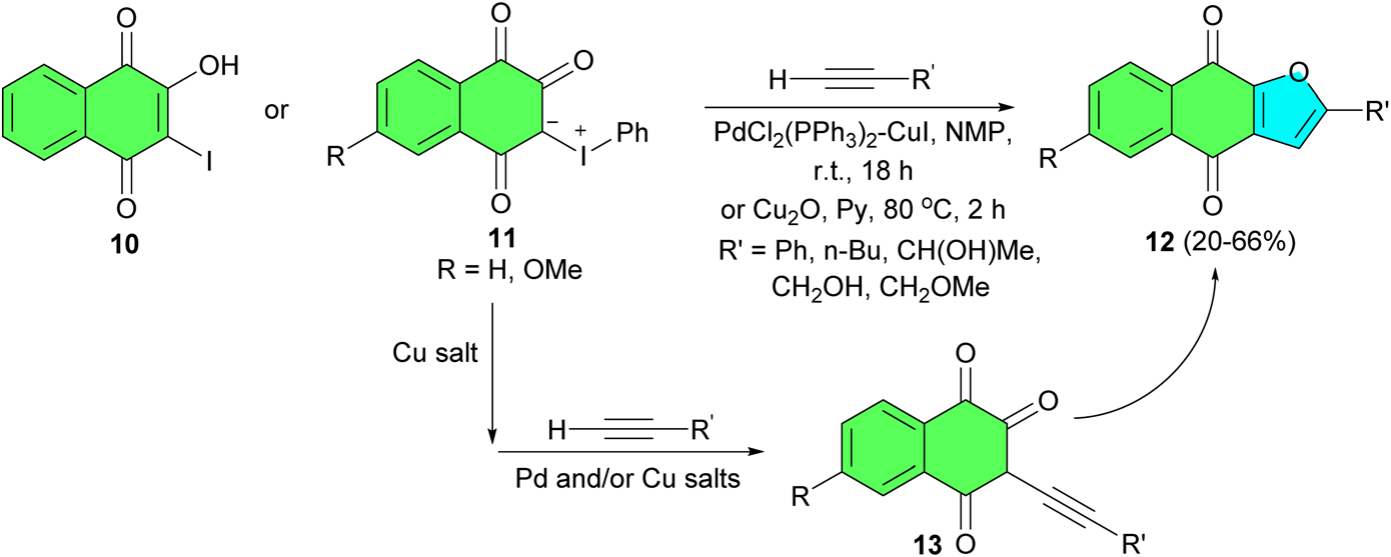
Transition metal-mediate synthesis of naphtho[2,3-*b*]furan-4,9-diones 12.

In 1997, Kobayashi and co-workers described treatment of 2-hydroxy-3-iodo-l,4-naphthoquinone (10) or 3-phenyliodonio-1,2,4-trioxo-1,2,3,4-tetrahydronaphthalenides 11 with terminal acetylenes in the presence of a bis(triphenylphosphine)palladium chloride-cuprous iodide catalyst at room temperature or cuprous oxidem *N*-methylpiperidine or pyridine at 80 °C, respectively, by sequential coupling/ring closure reactions furnished the corresponding 2-suhstituled naphtho[2,3-*b*]furan-4,9-diones 12 in 20–66% yields. In the proposed mechanism, the reactions using the iodonium ylides 11 proceed through formation of the corresponding 2-hydroxy-3-iodo-l,4-naphthoquinones as outlined in Scheme 6. Their formation, followed by coupling with a terminal acetylene, gives rise to the alkynylated hydroxy quinones 13, which subsequently undergo intramolecular ring closure to give 12.^34^

**Scheme 7:**
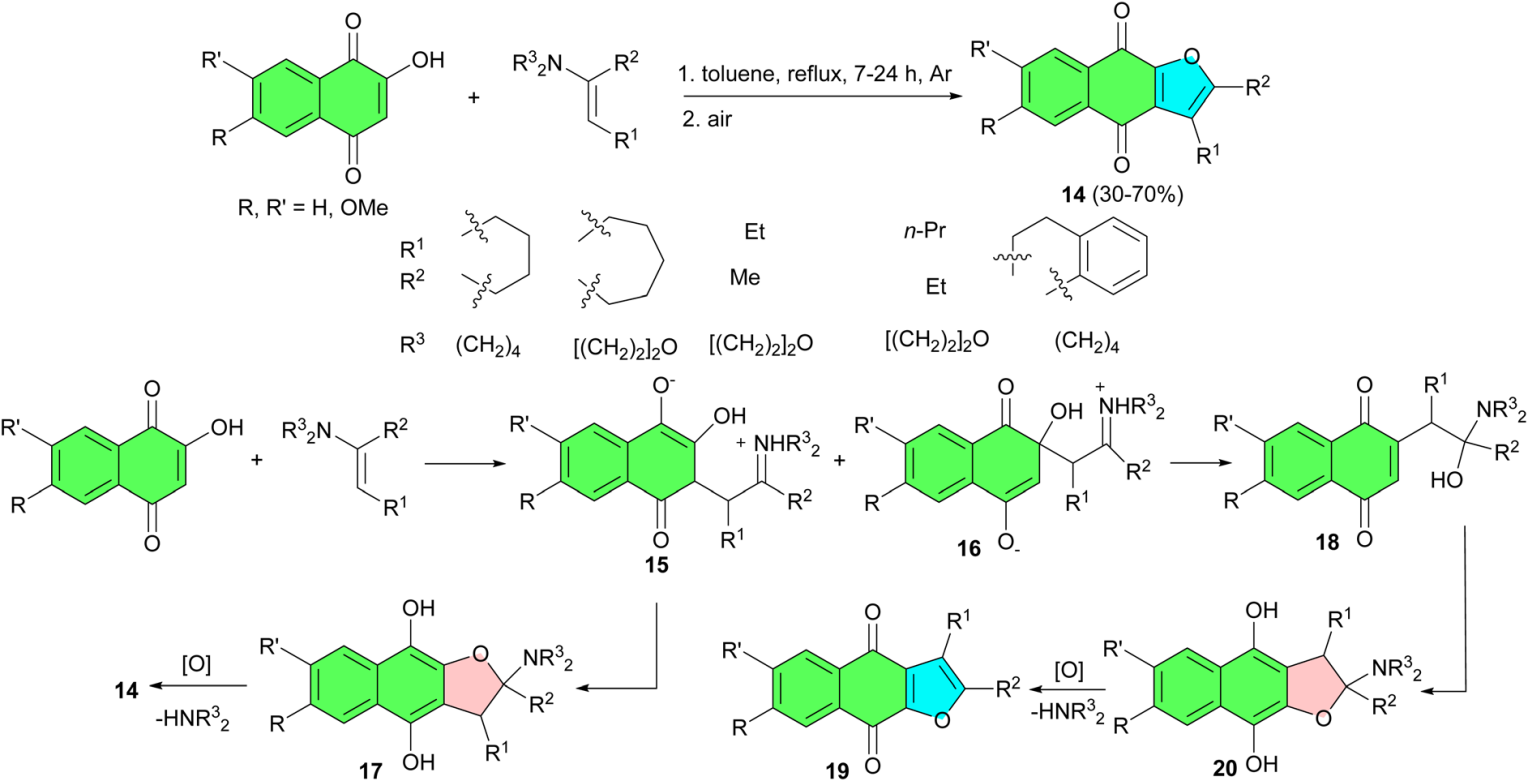
Preparation of naphtho[2,3-*b*]furan-4,9-diones 14.

The reaction of 2-hydroxy-1,4-naphthoquinones with enamines, derived from ketones, in refluxing toluene afforded the corresponding 2,3-disubstituted naphtho[2,3-*b*]furan-4,9-dione derivatives 14 after 7–24 h in moderate to good yields. The probable pathways leading to the formation of 14 are outlined in Scheme 7. Initially, the nucleophilic attack of the enamine to the quinone at both of the 2- and 3-positions to give the intermediate adducts 15 or 16. The 2-oxygen of 15 intramolecularly adds to the immonium carbons, resulting in the formation of a dihydrofuran ring. The dihydrofuran intermediate 17 gives rise to 14 through elimination of the amine and oxidation. On the other hand, the intermediate 16 rearranges to the aminal intermediate 18 through elimination of the hydroxide, which gives 19*via* the dihydrofuran intermediate 20. When *R* equals *R*′, compounds 14 and 19 are identical.^35^

**Scheme 8:**
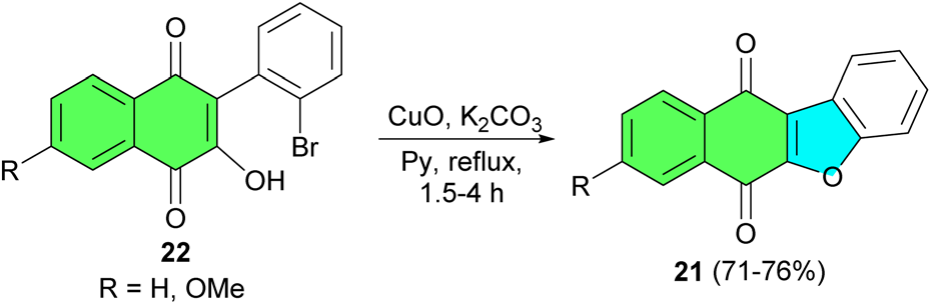
CuO catalyzed preparation of benzofuronaphthoquinone 21.

After that, Estevez and co-workers described synthesis of benzofuronaphthoquinone 21 in 71–76% yields by the reaction of 3-hydroxy-2-phenyl-1,4-naphthoquinones 22 using CuO, K_2_CO_3_ in pyridine under reflux conditions for 1.5–4 h (Scheme 8).^36^

**Scheme 9:**
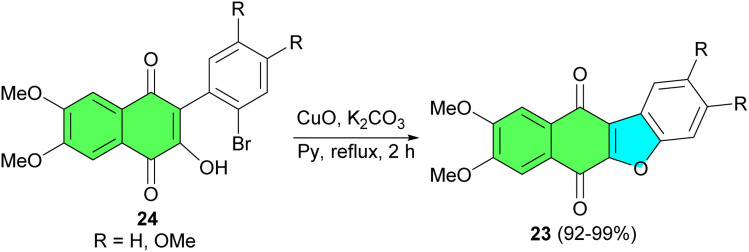
CuO catalyzed synthesis of benzofuronaphthoquinone 23.

Further, the same group reported synthesis of benzofuronaphthoquinone 23 in 92–99% yields by refluxing a solution of 24 in the presence of cuprous oxide, potassium carbonate and dry deoxygenated pyridine under argon for 2 h (Scheme 9).^37^

**Scheme 10:**
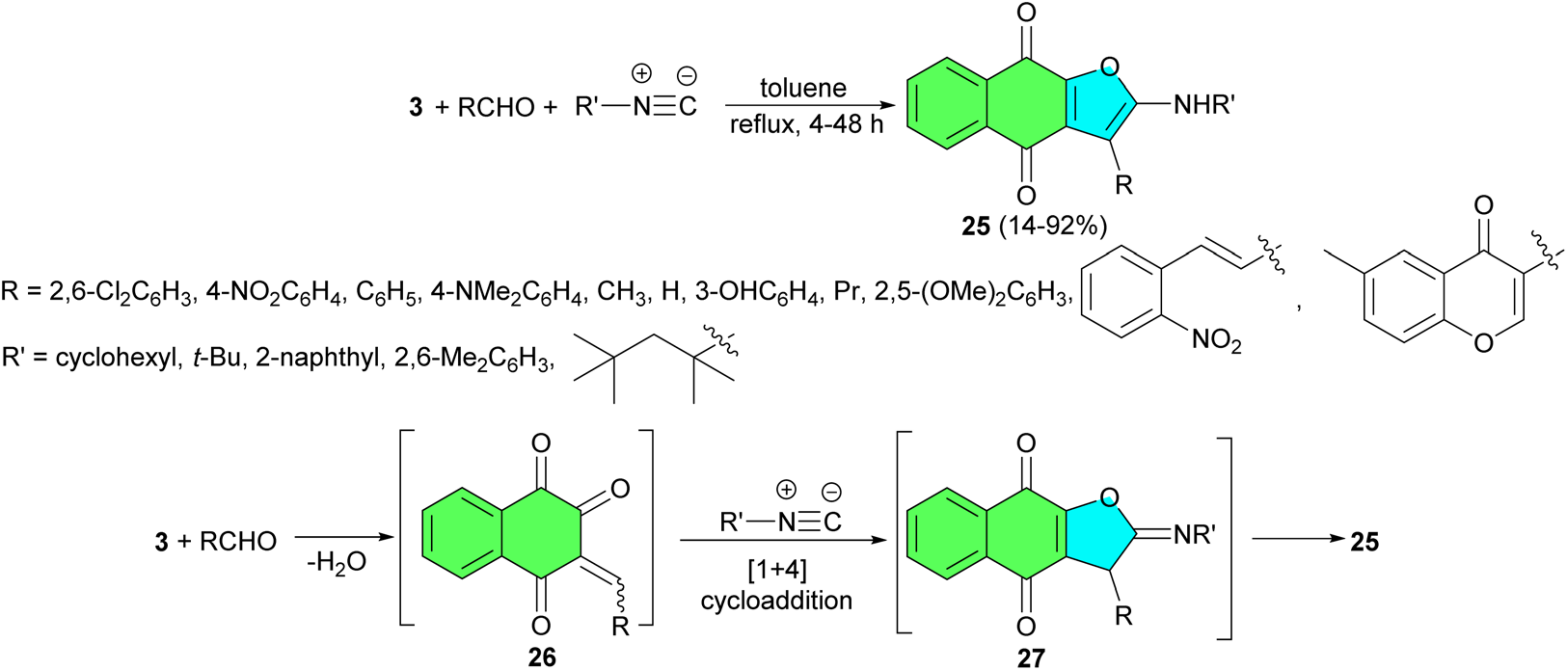
Regioselective synthesis of linear naphtho[2,3-*b*]-furan-4,9-diones 25.

In 2007, the Teimouri group described regioselective one-pot three-component condensation reaction of lawsone with isocyanides in the presence of a variety of aldehydes in refluxing toluene for 4–48 h afforded linear naphtho[2,3-*b*]-furan-4,9-dione derivatives 25 in 14–92% yields. The elucidation of regiochemistry accomplished by X-ray determination. In the proposed mechanism (Scheme 10), at first, a conjugated electron-deficient enone 26 obtained by a Knoevenagel condensation of 3 and the aldehyde. The next step of this mechanism could involve a [4 + 1] cycloaddition reaction of the electron-deficient heterodyne moiety of adduct 26 with the isocyanide to afford an iminolactone intermediate 27. The subsequent isomerization of iminolactone 27 leads to formation of product 25.^38^

**Scheme 11:**
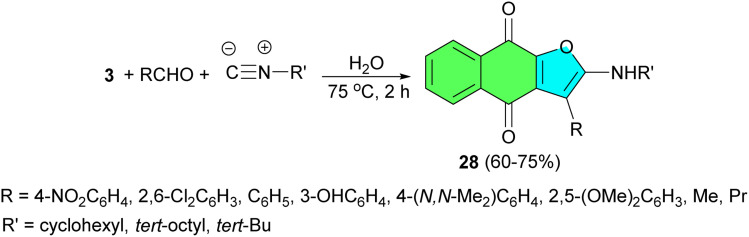
Synthesis of highly substituted linear naphtho[2,3-*b*]-furan-4,9-dione derivatives 28.

After that, an efficient regioselective and clean green synthesis of highly substituted linear naphtho[2,3-*b*]-furan-4,9-dione derivatives 28, starting from lawsone, alkyl isocyanides and a variety of aliphatic and aromatic aldehydes containing electron-withdrawing groups and electron-donating groups, is described. This [3 + 1 + 1] furannulation strategy affords furanonaphthoquinones in moderate to high yields, using water at 75 °C after 2 h (Scheme 11). The proposed mechanism is similar to that described in Scheme 10.^39^

**Scheme 12:**
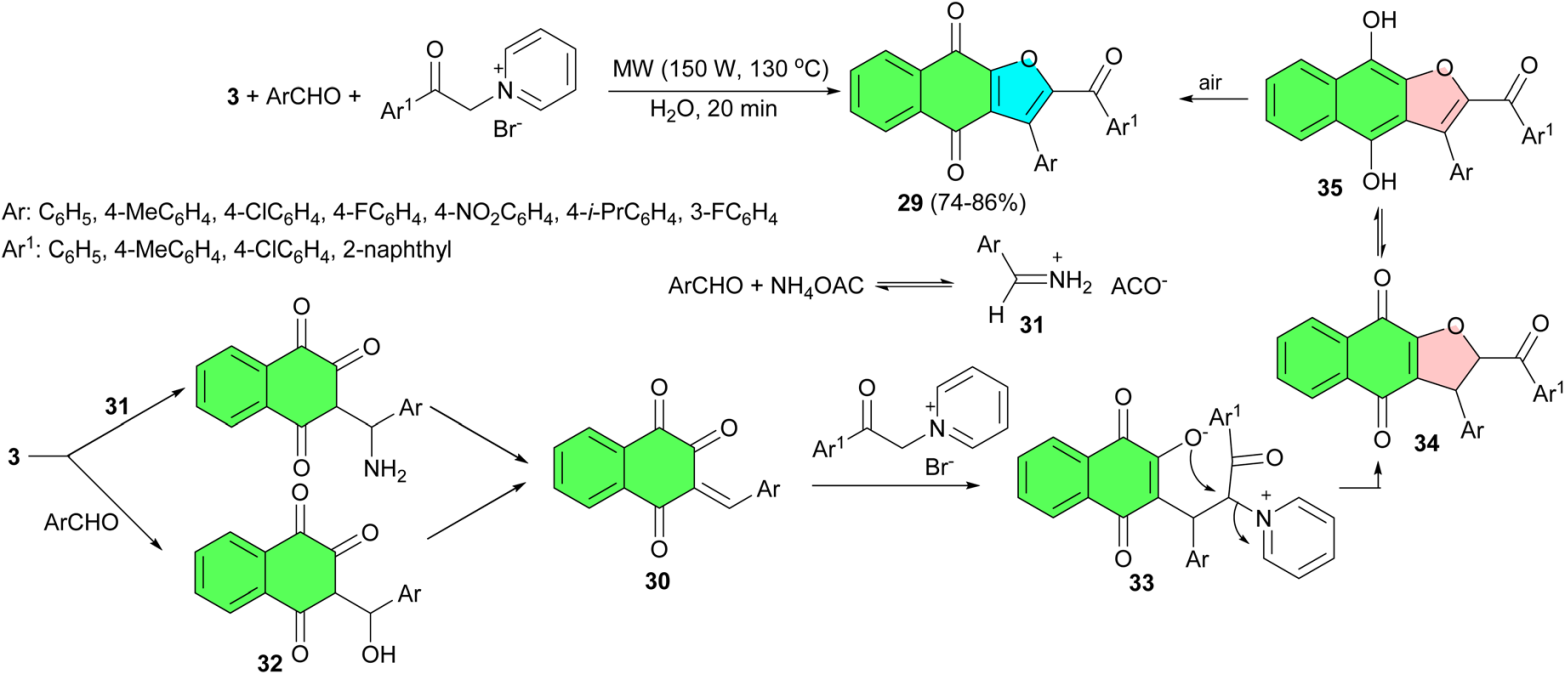
Microwave-assisted synthesis of functionalized naphtho[2,3-*b*]furan-4,9-diones 29.

In 2011, a three-component domino reaction of lawsone, aromatic aldehydes and a pyridinium salt in the presence of ammonium acetate, under microwave irradiation (at 130 °C and 150 W) and using water as solvent, furnished a library of 2-arylcarbonyl-3-aryl-4,9-dihydronaphtho[2,3-*b*]furan-4,9-diones 29 in 74–86% yields after 20 min. A plausible mechanism for the formation of 29 is depicted in Scheme 12. Presumably, the intermediate 30 could arise in two ways: (i) *via* the Mannich reaction of lawsone with an iminium ion 31 generated from aldehyde and ammonium acetate, followed by elimination of ammonia, or (ii) the ammonium acetate-catalyzed reaction of lawsone with the starting aldehyde to afford aldol 32, which would undergo dehydration. Then, the Michael addition of pyridinium ylide to 30 presumably affords the pyridinium enolate 33, which subsequently undergoes annulation *via* displacement of pyridine to give 34, which is probably in tautomeric equilibrium with the corresponding hydroquinone 35. Ultimately, these intermediates are transformed into the final products 29*via* air oxidation.^40^

**Scheme 13:**
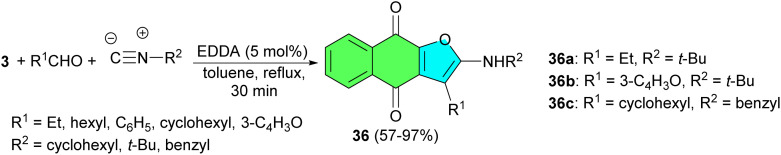
EDDA catalyzed synthesis of 2-amino-naphtho[2,3-*b*]furan-4,9-diones 36.

After that, 2-amino-naphtho[2,3-*b*]furan-4,9-diones 36 were synthesized in 57–97% yields using a one-pot three-component reaction of lawsone with various aldehydes and aromatic or aliphatic isocyanides in the presence of catalytic amounts of EDDA (ethylenediamino diacetate) under refluxing in toluene for 30 min under nitrogen atmosphere (Scheme 13). These compounds were tested for cytotoxicity against several human solid tumor cell lines (MCF7, MCF7/BUS, and SK-Br-3). Compounds 36a, 36b, and 36c exhibit the highest cytotoxicities. Compound 36a shows selectivity toward the hormone-dependent cell line MCF7/BUS with a GI_50_ value of 9.2 μM. Compounds 36b and 36c present good cytotoxicities against MCF7, MCF7/BUS, and SK-Br-3 tumor cell lines. The best GI_50_ was achieved by compound 36b against the SK-Br-3 cell line, with GI_50_ = 1.6 μM. Moreover, the electronic properties of these aromatic donor–acceptor derivatives were analyzed by means of their redox potentials and solvatochromic properties.^41^

**Scheme 14:**
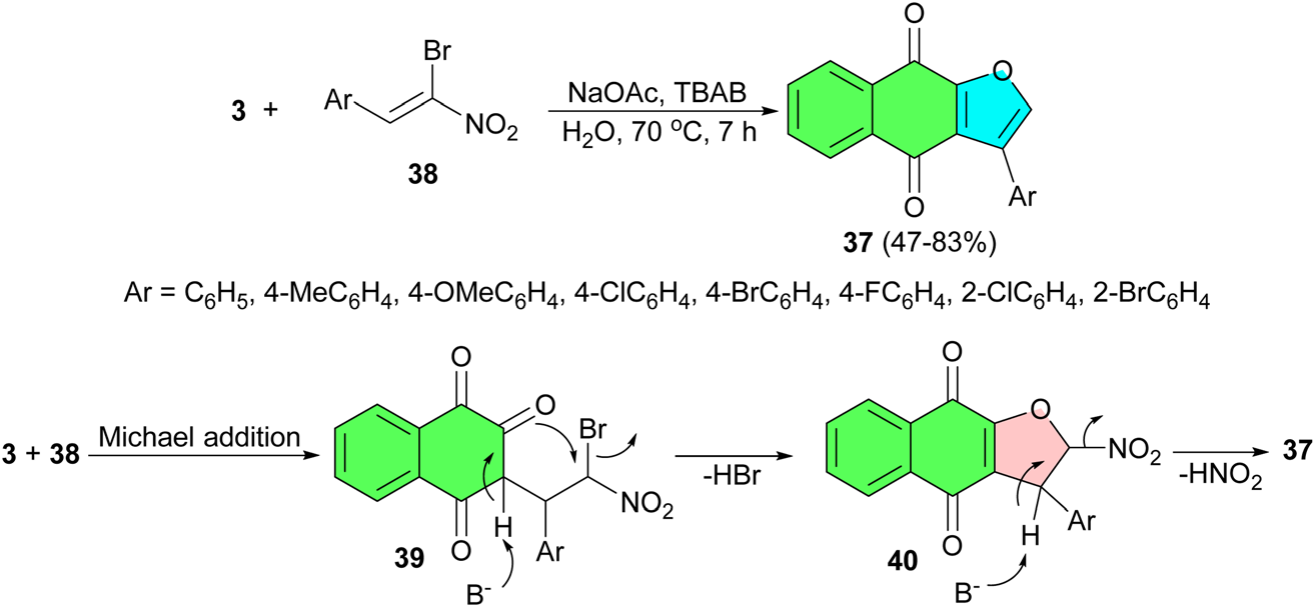
Preparation of 3-phenylnaphtho[2,3-*b*]furan-4,9-diones 37.

Further, a simple and efficient protocol developed for the synthesis of 3-phenylnaphtho[2,3-*b*]furan-4,9-diones 37 in 47–83% yields by domino reaction of α-bromonitroalkenes 38 to lawsone using NaOAc and TBAB (tetrabutylammonium bromide) in H_2_O at 70 °C for 7 h. A mechanistic rationalization for this reaction is provided in Scheme 14. The domino reaction of α-bromonitroalkenes with 3 gives the Michael addition product 39 catalyzed by NaOAc. Then, the enolate anion was formed under the basic conditions and the subsequent intramolecular nucleophilic displacement of 39 affords intermediate 40. Subsequently, elimination of the nitro group leads to the formation of the desired product 37. The absorption characteristics of the compounds were examined by UV-vis spectra and fluorescence spectroscopy. All compounds were fluorescent in solution emitting at blue light (432–433 nm), green light (512–536 nm), or yellow light (591 nm).^42^

**Scheme 15:**
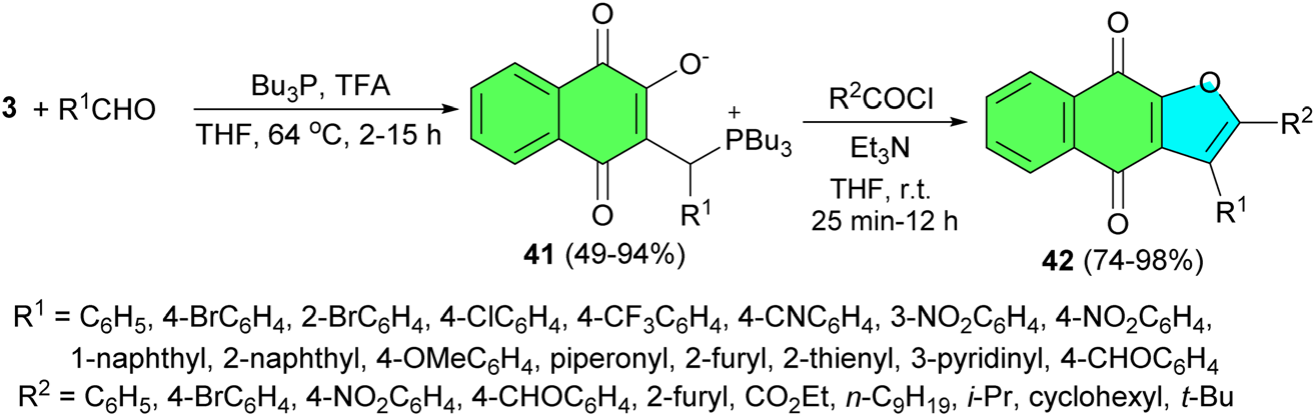
Regioselective synthesis of polysubstituted furanonaphthoquinones 42.

The Lin group described an efficient and attractive synthesis of a series of poly-functionalized phosphorus zwitterions 41 in 49–94% yields was achieved *via* three-component reactions of lawsone, aldehydes, and Bu_3_P in the presence of acidic promoter in THF at 64 °C for 2–15 h. These polysubstituted zwitterions could regioselectively undergo further transformations to synthetically important furanonaphthoquinones 42 in 74–98% yields *via* the intramolecular Wittig reaction in THF at room temperature for 25 min to 12 h (Scheme 15). The outstanding features of the synthetic methodology are the flexibility to synthesize furanonaphthoquinone derivatives with various substituents at the 2- and 3-positions of the furan segment, and a wide selection of substituents that can be easily obtained from commercially available aldehydes and acid chlorides.^43^

**Scheme 16:**
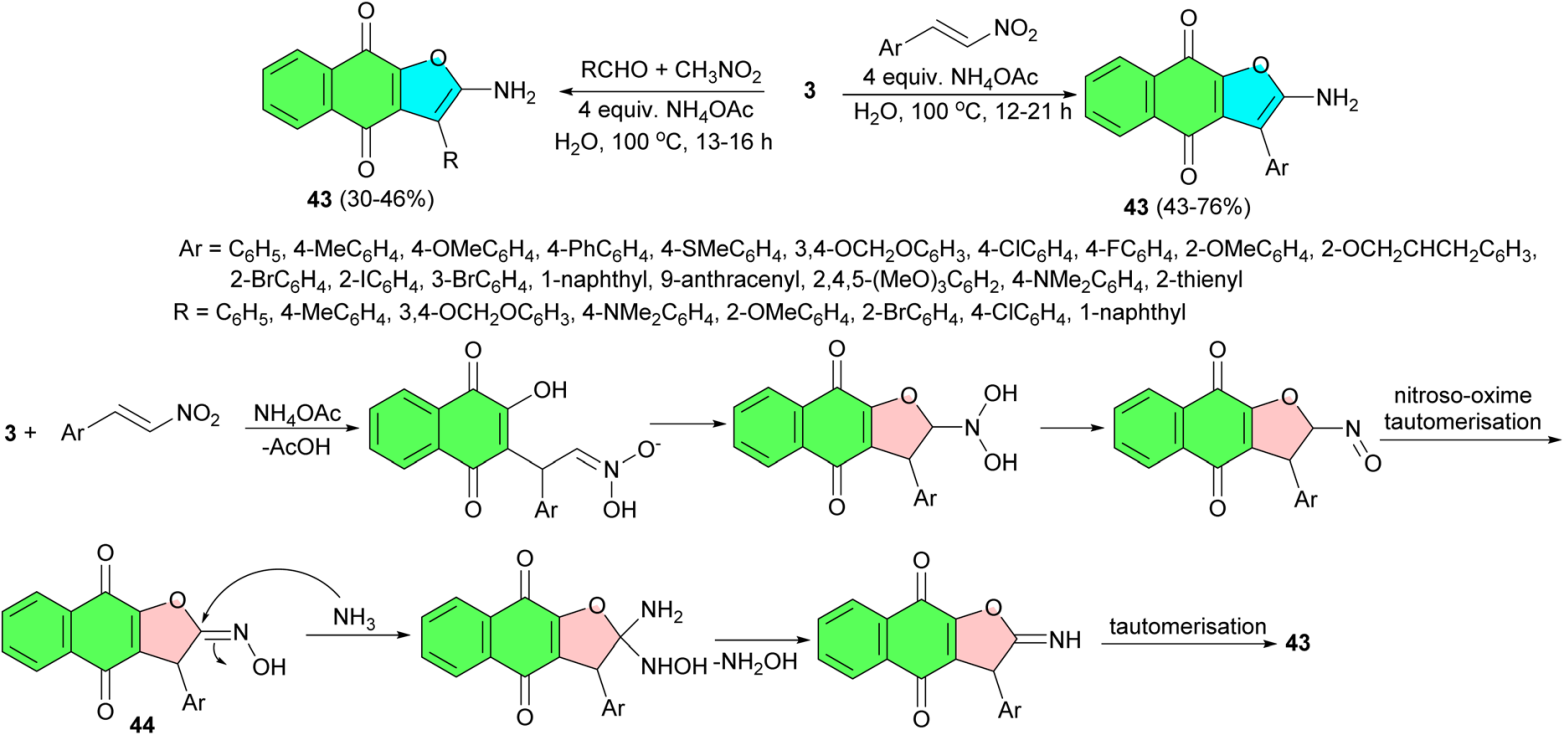
Synthesis of 3-substituted 2-aminonaphtho[2,3-*b*]furan-4,9-diones 43.

In 2013, Yao and co-workers demonstrated an efficient base-catalyzed synthesis of 3-substituted 2-aminonaphtho[2,3-*b*]furan-4,9-dione derivatives 43 in 43–76% yields from lawsone and nitroalkenes under aqueous conditions at 100 °C for 12–21 h. Moreover, synthesis of 43 could be achieved directly in a three-component reaction of lawsone, aldehyde, and nitromethane under the same conditions. The most important feature of this methodology is the conversion of the nitro group into an amino group without any reducing agent. The mechanism was thought to involve the formation of a Michael adduct from the reaction of a nitroalkene and lawsone, which undergoes cyclization in the presence of ammonium acetate to produce the oxime derivative 44. The oxime derivative is then transformed into 43 by the nucleophilic addition of ammonia to the oxime followed by the elimination of hydroxylamine (Scheme 16).^44^

**Scheme 17:**
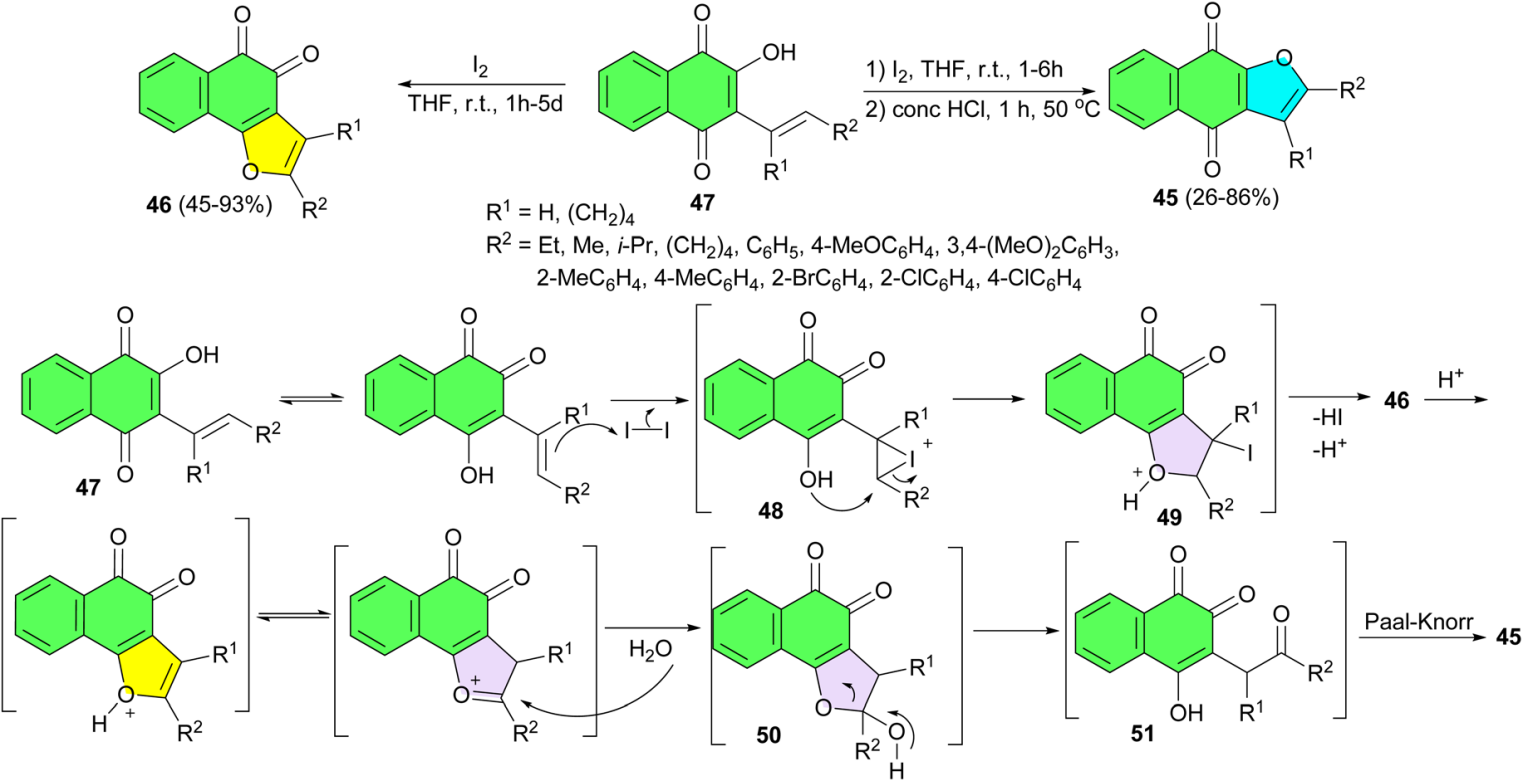
Iodine-mediated regioselective synthesis of naphthofuroquinones 45–46.

In 2015, a facile method for highly regioselective synthesis of both linear and angular naphthofuroquinones 45 and 46 in 26–93% yields were developed *via* iodine mediated cyclization of 2-hydroxy-3-substitutedvinyl-1,4-naphthoquinones 47 in THF at room temperature. The possible reaction mechanism is proposed in Scheme 17. The reaction was supposed to be initiated by the nucleophilic attack of the olefinic double bond to iodine to form the iodonium ion 48. The following decomposition of 48 might involve the attack from the neighboring hydroxyl group to form the unstable iodide intermediate 49 which would automatically eliminate one molecular of HI to give the angular naphthofuroquinone 46. Under acidic condition, hemiketal 50 could be formed through the nucleophilic attack of H_2_O to the C-2 position of the protonated angular naphthofuroquinone 46. The following decomposition of hemiketal 50 would give the 1,4-diketone intermediate 51, which would finally afford the linear naphthofuroquinone 45 through a classic Paal–Knorr process. Considering the very mild reaction condition, high yields, high angular/linear selectivity, and non-involvement of transitional metals, this method might find great application in the synthesis of naphthofuroquinones.^45^

**Scheme 18:**
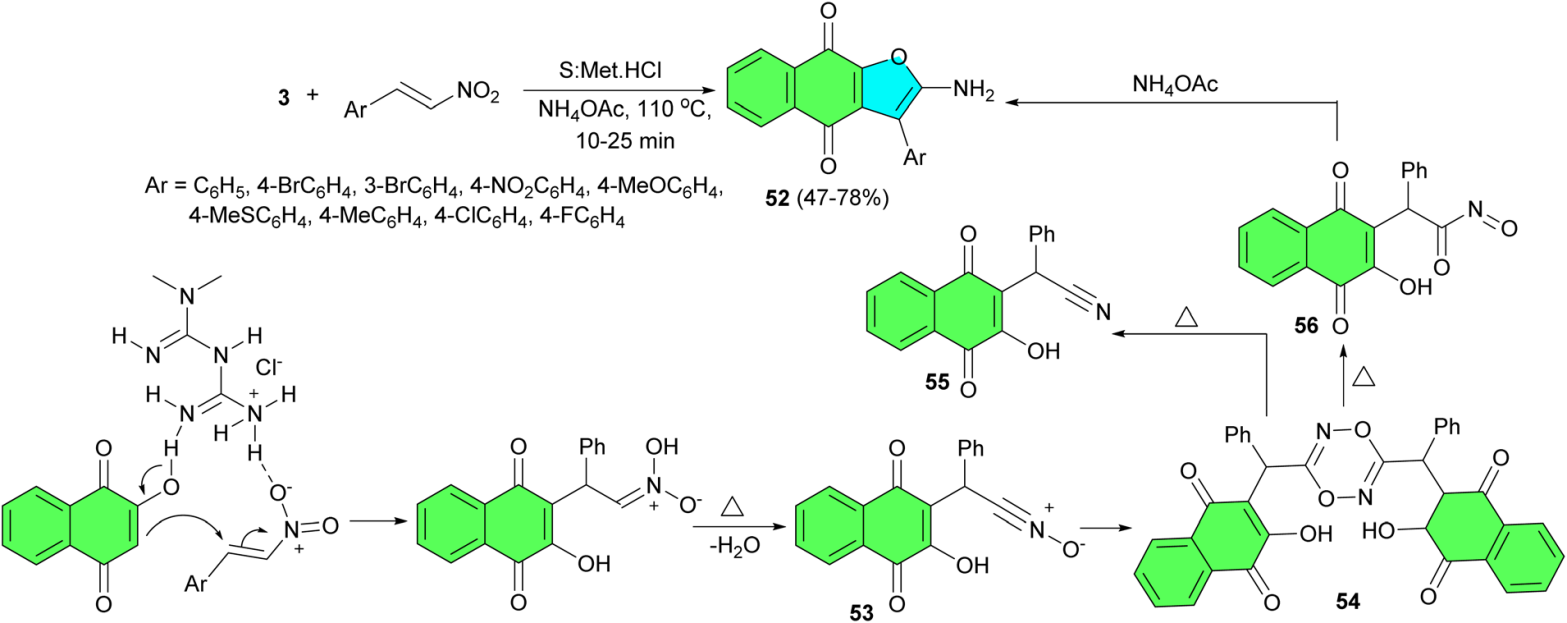
Synthesis of 2-amino-3-arylnaphtho[2,3-*b*]furan-4,9-diones 52.

In 2016, an efficient and environmentally benign one-pot method reported for the diverse synthesis of 3-substituted 2-aminonaphtho[2,3-*b*]furan-4,9-diones 52 in 47–78% yields *via* the domino reaction between lawsone, β-nitrostyrenes and ammonium acetate in a new deep eutectic solvent made of sorbitol and metformin HCl at 110 °C for 10–25 min. A pronounced positive solvatochromism was observed for the electron donor–acceptor conjugated system of these products, resulting in bathochromic and hyperchromic shifts of their visible absorption band in polar and protic solvents. A plausible mechanism is outlined in Scheme 18. The progress of the reaction involves thermal dehydration of the Michael adducts that occurs at temperatures above 90 °C to give the intermediate nitrile-*N*-oxides 53. These zwitterionic intermediates undergo a dipolar dimerization reaction to yield the intermediate 1,4,2,5-dioxadiazines 54 followed by a chain of thermal demanding reactions involving electrocyclic scission of the dioxadiazine ring and conversion of the resulting two fragments into naphtho[2,3-*b*]furan-4,9-diones. Upon heating, the fragmented intermediate 55 is presumed to undergo a direct cyclization while its twin 56 cyclocondenses with ammonia to deliver the product 52.^46^

**Scheme 19:**
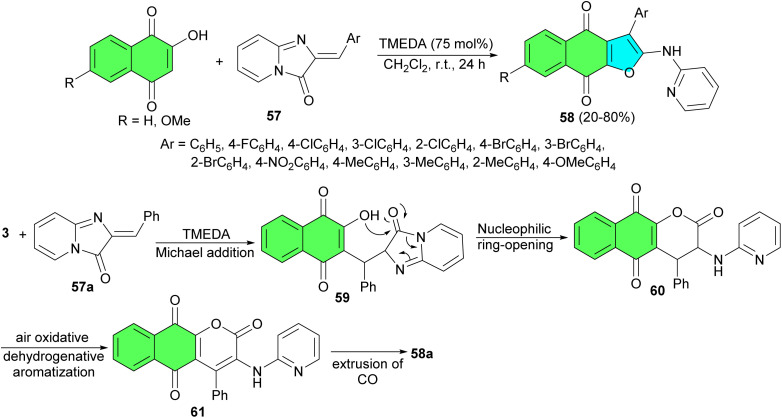
Synthesis of naphtho[2,3-*b*]furan-4,9-diones 58 under aerobic condition.

In 2017, Zhou *et al.* developed an unprecedented base-promoted oxidative coupling of lawsone derivatives with (*Z*)-2-ylideneimidazo[1,2-*a*]pyridin-3(2*H*)-ones 57, which provide a convenient approach to access naphtho[2,3-*b*]furan-4,9-dione derivatives 58 with a 2-aminopyridine moiety using TMEDA in 20–80% yields under mild reaction conditions. These compounds in ethanol showed the characteristic intense charge-transfer bands (π–π* transitions) occurring in the visible region. Compound 58a exhibited selective response to Hg^2+^ and Pd^2+^*via* a chelate-binding module and can be developed as a sensitive chromogenic sensor for Hg^2+^ in the presence of a range of competing cations in aqueous media. A tentative mechanism is postulated in Scheme 19. With the aid of the base catalyst, Michael addition of 3 to 57a afforded a adduct intermediate 59. Then, a suffered an intramolecular nucleophilic ring opening of the imidazo[1,2-*a*]pyridin-3(2*H*)-one moiety to provide a 3,4-dihydrobenzo[*g*]chromene-2,5,10-trione intermediate 60. Under aerobic conditions, benzo[*g*]chromene-2,5,10-trione 61 was formed through the air oxidative dehydrogenative aromatization of 60. Finally, extrusion of carbon monoxide from intermediate 61 resulted in the generation of thermodynamically more stable naphtho[2,3-*b*]furan-4,9-dione 58a.^47^

**Scheme 20:**
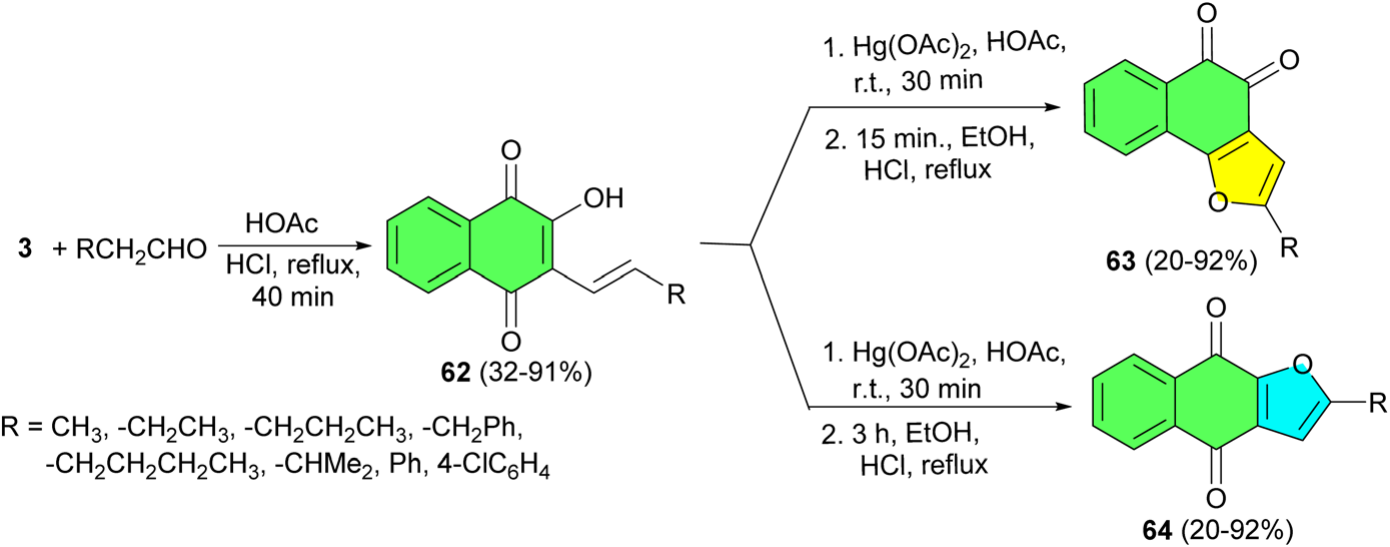
Synthesis of furanonaphthoquinones 63–64.

Next, the HNQs 62 synthesized in 32–91% yields by aldol condensation between lawsone and appropriate aldehydes in glacial acetic acid, followed by concentrated HCl under reflux conditions for 40 min. The FNQs 63 and 64 were prepared by oxidative cyclization of the corresponding 62 with Hg(OAc)_2_ in glacial acetic acid at room temperature for 30 min and at 80 °C for 15 min. All compounds disclosed higher *in vitro* antiplasmodial activity than lapachol. *Ortho*- and *para*-naphthoquinones with a furan ring fused to the quinonoid moiety were more potent than 2-hydroxy-3-(1′-alkenyl)-1,4-naphthoquinones, while *ortho*-furanonaphthoquinones were more cytotoxic (Scheme 20).^48^

**Scheme 21:**
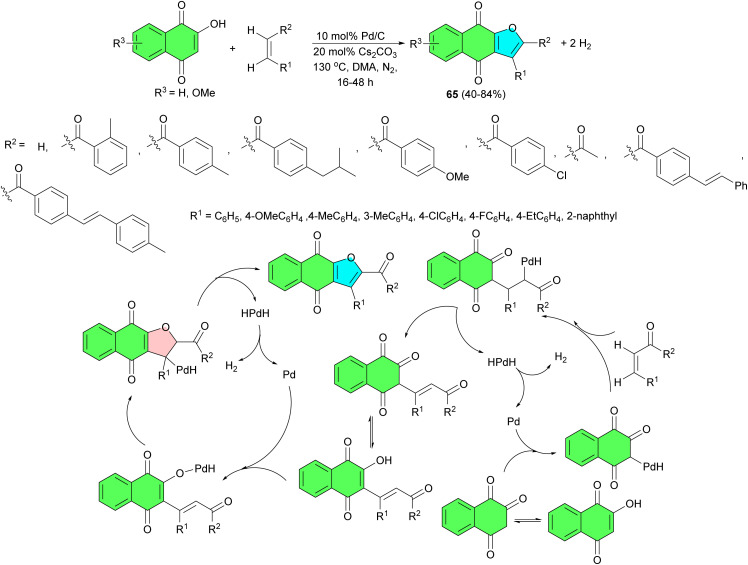
Palladium-catalyzed synthesis of naphtho[2,3-*b*]furan-4,9-diones 65.

In 2019, a reverse hydrogenolysis process developed for two-site coupling of 2-hydroxy-1,4-naphthoquinones with olefins to produce naphtha[2,3-*b*]furan-4,9-diones 65 in 40–84% yields and hydrogen. The reaction is catalyzed by commercially available Pd/C (10 mol%) without oxidants and hydrogen acceptors in DMA at 130 °C for 16–48 h. The results showed that enones with a variety of groups such as methyl, ethyl, isobutyl, and methoxyl could be directly coupled with 2-hydroxy-1,4-naphthoquinone to give moderate to high isolated yields. Fluorine- and chlorine-containing enones were unaffected. Non-aromatic enones could also be smoothly coupled with the 2-hydroxy-1,4-naphthoquinones to afford the corresponding products with moderate isolated yields. The proposed mechanism is illustrated in Scheme 21.^49^

**Scheme 22:**
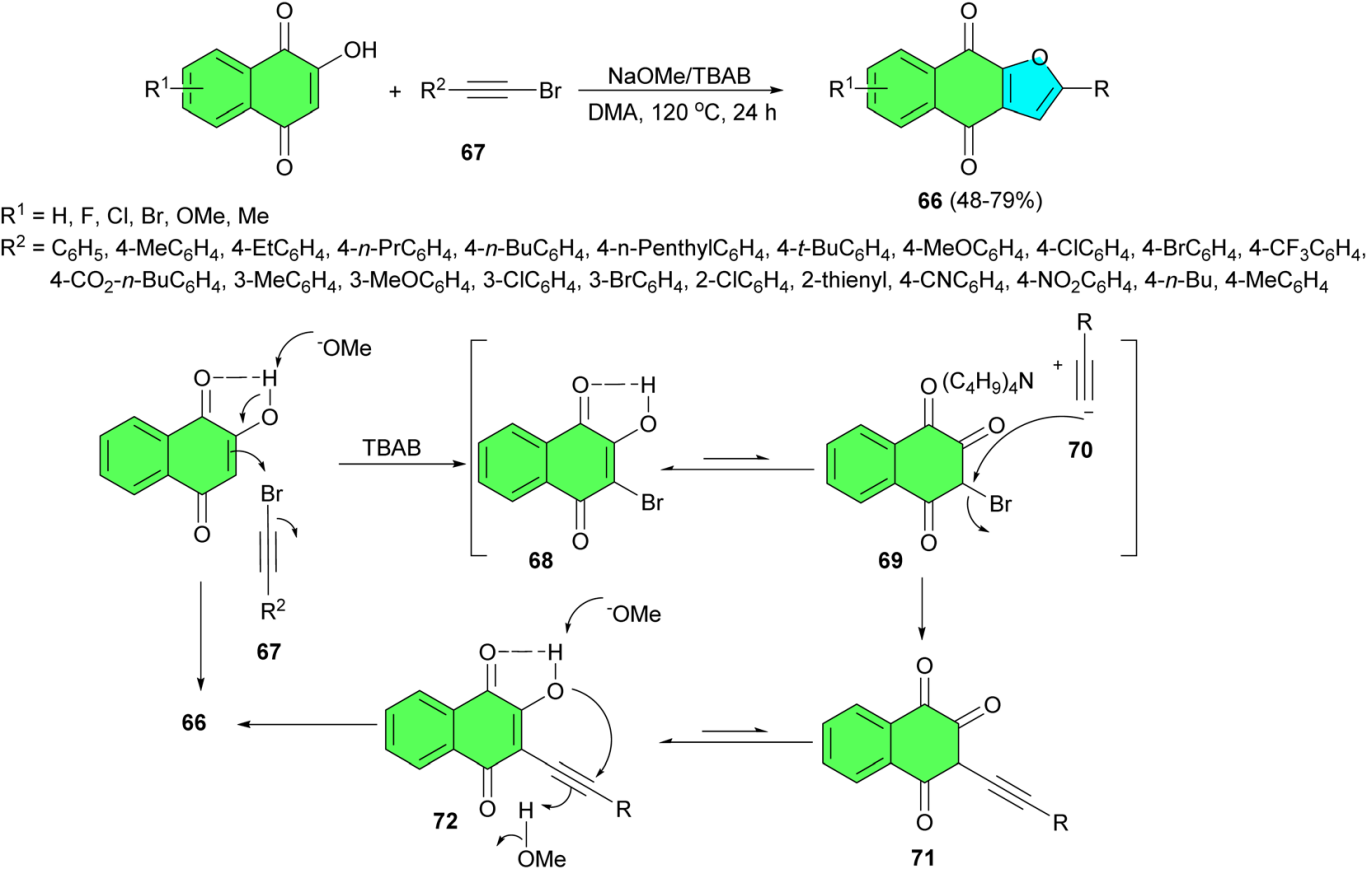
Transition-metal-free synthesis of naphthoquinonefurans 66.

In 2020, Peng *et al.* explored a transition-metal-free route for tandem one-pot synthesis of naphthoquinonefuran derivatives 66 in 48–79% yields from 2-hydroxynaphthoquinones and arylethynyl bromides 67 using NaOMe/TBAB in DMA at 120 °C for 24 h. A plausible reaction pathway is outlined in Scheme 22. A sodium methoxide-promoted deprotonation of 2-hydroxynaphthoquinones produced sodium enolate, which was then brominated at the 3-position to form the intermediate 68 using the alkynyl bromide 67 as the source of Br^+^. The tautomeric isomer 69 in the keto-form from 68 underwent a nucleophilic attack by the produced arylacetylide 70 to give the alkynated intermediate 71, which was transformed into 3-alkynated hydroxynaphthoquinone 72 through a keto–enol tautomeric process. A sodium methoxide-promoted deprotonation and subsequent intramolecular nucleophilic addition of enolate anion to conjugated ynone furnished the cyclized product 66.^50^

**Scheme 23:**
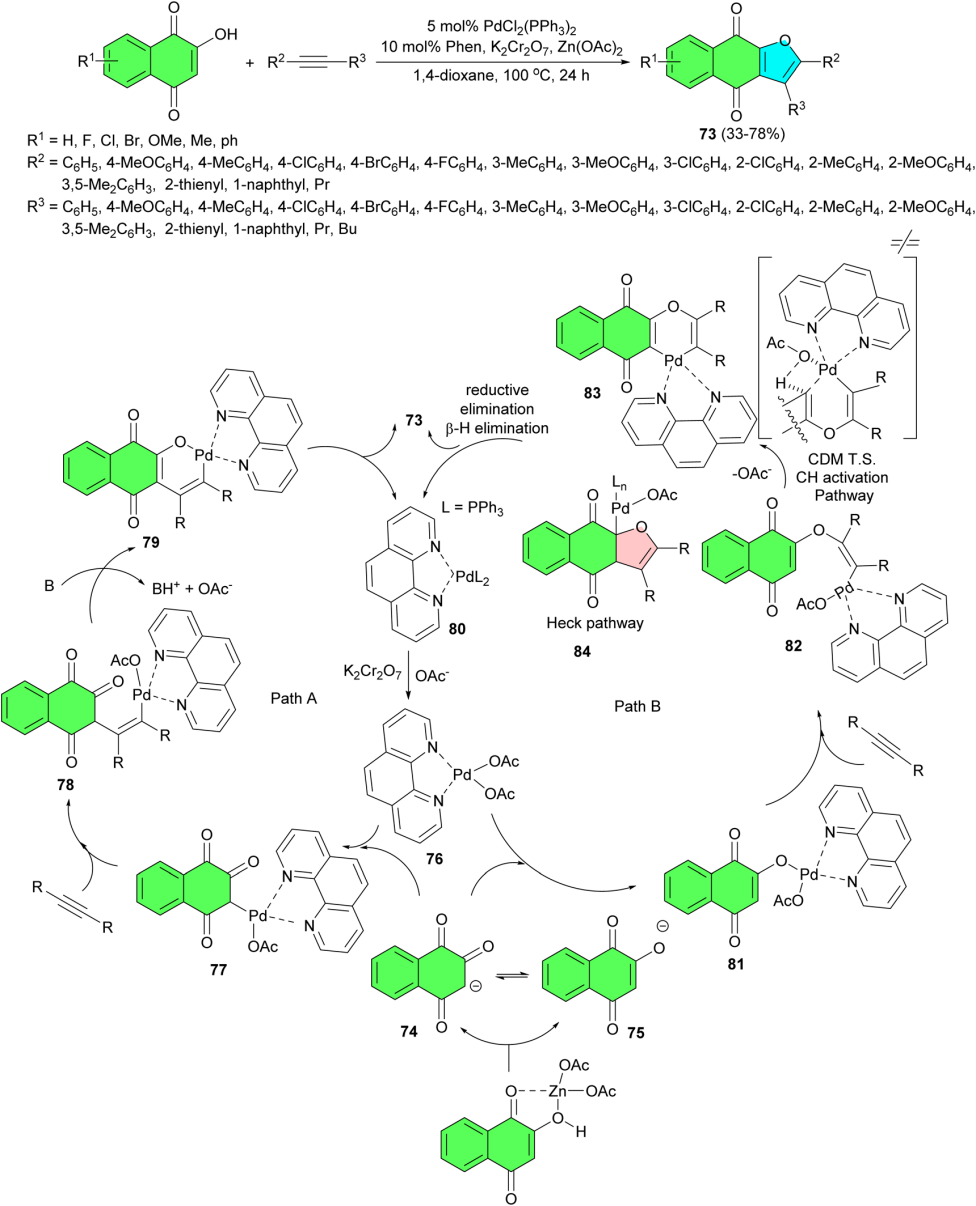
Palladium(ii)-catalyzed synthesis of naphtho[2,3-*b*]furan-4,9-diones 73.

The Peng group developed a modular approach for rapid syntheses of diverse naphtho[2,3-*b*]furan-4,9-dione derivatives 73 through Pd-catalyzed oxidative annulations of 2-hydroxynaphthalene-1,4-diones with readily accessible unactivated internal alkynes in 1,4-dioxane at 100 °C for 24 h. The combination of Zn(OAc)_2_ and K_2_Cr_2_O_7_ was found to be essential for the efficient formation of furonaphthoquinones in 33–78% yields. This synthetic method exhibits a broad substrate scope with good yields and excellent regioselectivity for aryl, alkyl substituted alkynes. Two competitive catalytic cycles for the synthesis of 73 are proposed in Scheme 23. In the presence of Zn(OAc)_2_ as the base, the deprotonation of 2-hydroxynaphthalene-1,4-dione yielded two tautomeric anions, which existed as either carbanion 74 in the keto-form or as oxygen anion 75 in the enol-form. At this stage, attack of anion onto the electrophilic Pd(ii) species may occur in two different ways (paths A and B). Path A involved the attack of carbanion 74 onto catalytic active species 76 to form alkyl-Pd(ii) species 77. Subsequently, the coordination of the internal alkyne to 77 would induce its carbopalladation to afford an alkenyl palladium(ii) complex 78. Base-assisted further deprotonation of the ketone α-carbon of 78 led to O–Pd bond formation, affording intermediate 79. Palladacycle 79 underwent C–O reductive elimination to afford the desired product 73 and a Pd(0) species 80, which was oxidized by K_2_Cr_2_O_7_ to regenerate the active Pd(ii) species 76 for the next catalytic cycle. On the other hand, mechanistic cycle B was initiated by the attack of enol anion onto the electrophilic Pd(ii) species 76, giving the enol-type palladium(ii) 81. The coordination followed by syn migratory insertion of internal alkyne into the O–Pd bond then afforded alkenyl-Pd(ii) species 82. The final product 73 can be formed possibly through two distinct pathways (C–H activation or Heck pathway). The C–H activation pathway involved a concerted metalation deprotonation (CMD) transition state of alkene to form the palladacycle 83, which underwent C–C bond forming reductive elimination to afford the desired furonaphthoquinone 73 and regenerate a Pd(0) species 80. The Heck pathway involved an intramolecular *syn* migratory insertion into the olefin moiety of 82 and then was followed by an isomerization process to give the σ-alkyl-palladium(ii) acetate 84 with β-hydrogen in a *syn* position relative to the palladium atom. A *syn* β-hydride elimination afforded 73 and hydridopalladium(ii) acetate, which underwent a reversible reductive elimination to regenerate Pd(0) complex 80. Finally, Pd(0) resulting from an elimination process was oxidized to Pd(ii) by K_2_Cr_2_O_7_.^51^

**Scheme 24:**
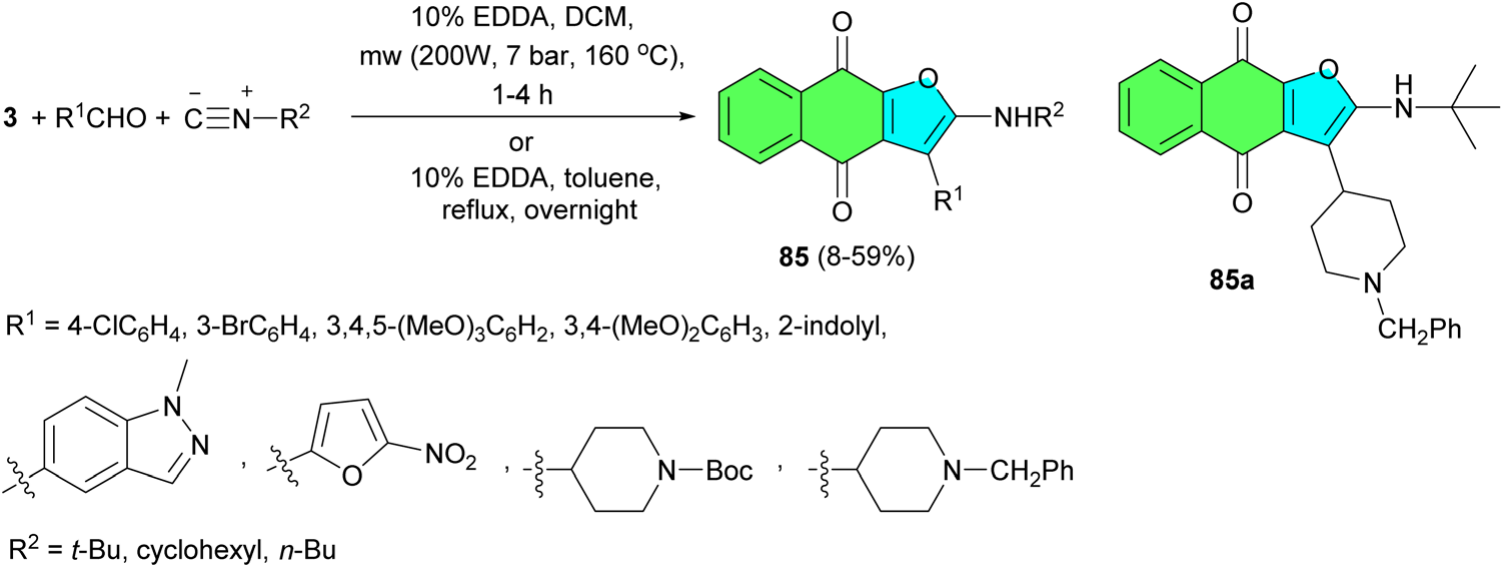
EDDA catalyzed synthesis of naphthofuroquinones 85.

In 2022, the García-Sosa reported synthetic approach to naphthofuroquinones 85 in moderate to good yields *via* a reaction involving lawsone, various aldehydes, and three isocyanides using EDDA in dichloromethane under microwave irradiation for 1–4 h at 160 °C and conventional method in the presence of EDDA in toluene under reflux overnight. In addition, for less-reactive aldehydes, two naphtho-enaminodione quinones were obtained. All compounds were evaluated for their anti-infectious activities. Among the naphthofuroquinone series, 85a exhibited comparatively the best activity against *P. falciparum* (IC_50_ = 2.5 μM) and *M. tuberculosis* (MIC = 9 μM) with better (*P. falciparum*) or equivalent (*M. tuberculosis*) values to already-known naphthofuroquinone compounds (Scheme 24).^52^

## 3. Synthesis of dihydronaphthofuroquinones

**Scheme 25:**
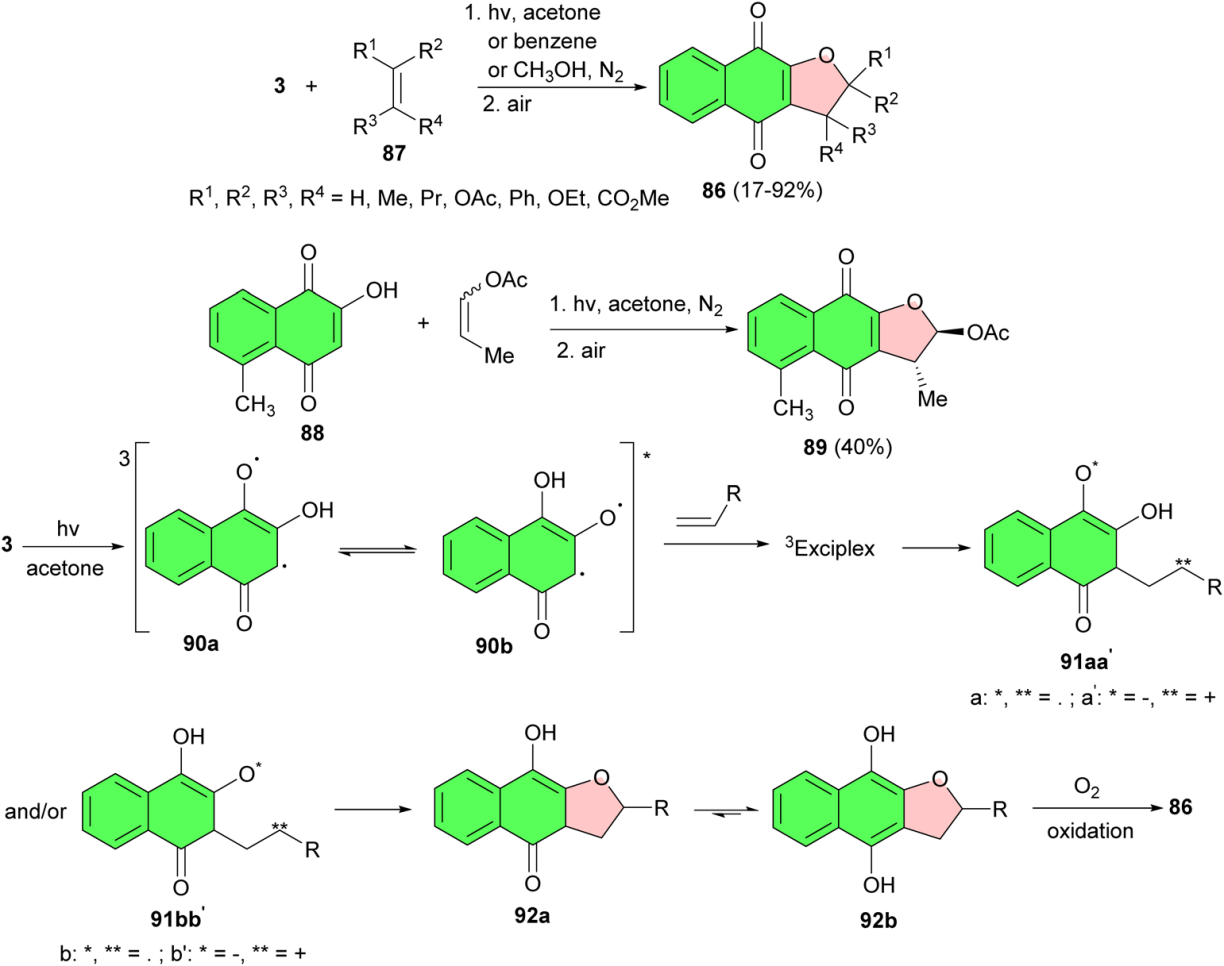
One-step synthesis of 2,3-dihydronaphtho[2,3-*b*]furan-4,9-diones 86.

In 1991, Suginome *et al.* synthesized a series of 2,3-dihydronaphtho [2,3-*b*]furan-4,9-diones 86 in 17–92% yields by a new [2 + 3] type regioselective photoaddition of 2-hydroxy-1,4-naphthoquinones with a variety of alkenes 87 in acetone or benzene after 4–24 h. The yield of adduct decreased appreciably when the photoaddition was conducted in methanol. Moreover, photoaddition of 2-hydroxyd-methyl-l,4-naphthoquinone (88) with 1-propenyl acetate in acetone gave *trans*-2-acetoxy-2,3-dihydro-3,5-dimethylnaphtho[2,3-*b*]-furan-4,9-dione (89) in 40% yield. Mechanistically, in this process, irradiation of 3 in acetone or benzene generates tautomeric exited triplets (90a) and (90b), which react with an alkene through a triplet exciplex to give biradical (91aa′) and/or (91bb′). Intramolecular cyclization of the intermediate gives hydroquinones (92a) and (92b). 2,3-Dihydronaphthofuran-4,9-dione 86 is then formed by air oxidation of the hydroquinone during the workup and isolation procedures (Scheme 25).^53^

**Scheme 26:**
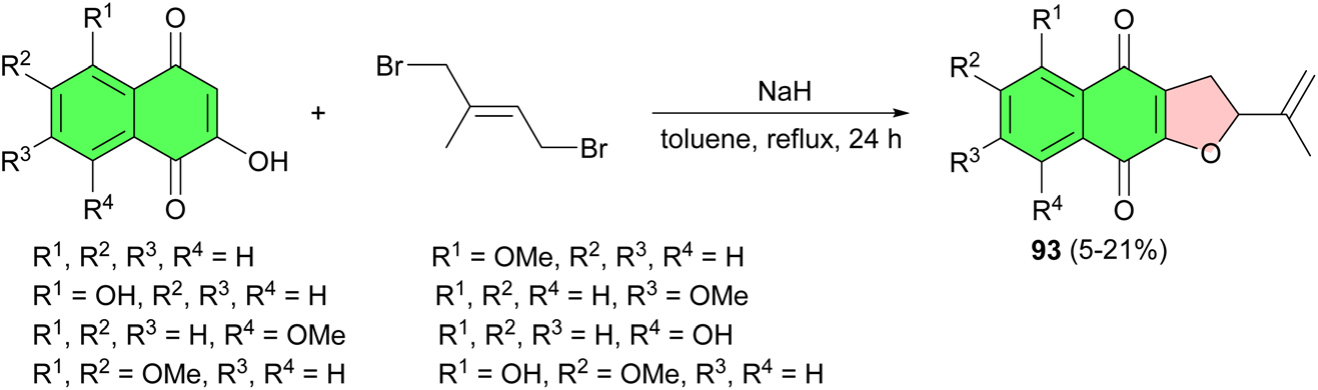
One-step preparation of 2-isopropenyl-2,3-dihydronaphtho-[2,3-*b*]furan-4,9-diones 93.

In 2001, Yamaguchi *et al.* one-step preparation of some 2-isopropenyl-2,3-dihydronaphtho-[2,3-*b*]furan-4,9-diones 93 in 5–21% yields described by the reaction of lawsone derivatives with 1,4-dibromo-2-methylbut-2-ene in dry toluene in the presence of sodium hydride under reflux condition for 24 h (Scheme 26).^54^

**Scheme 27:**
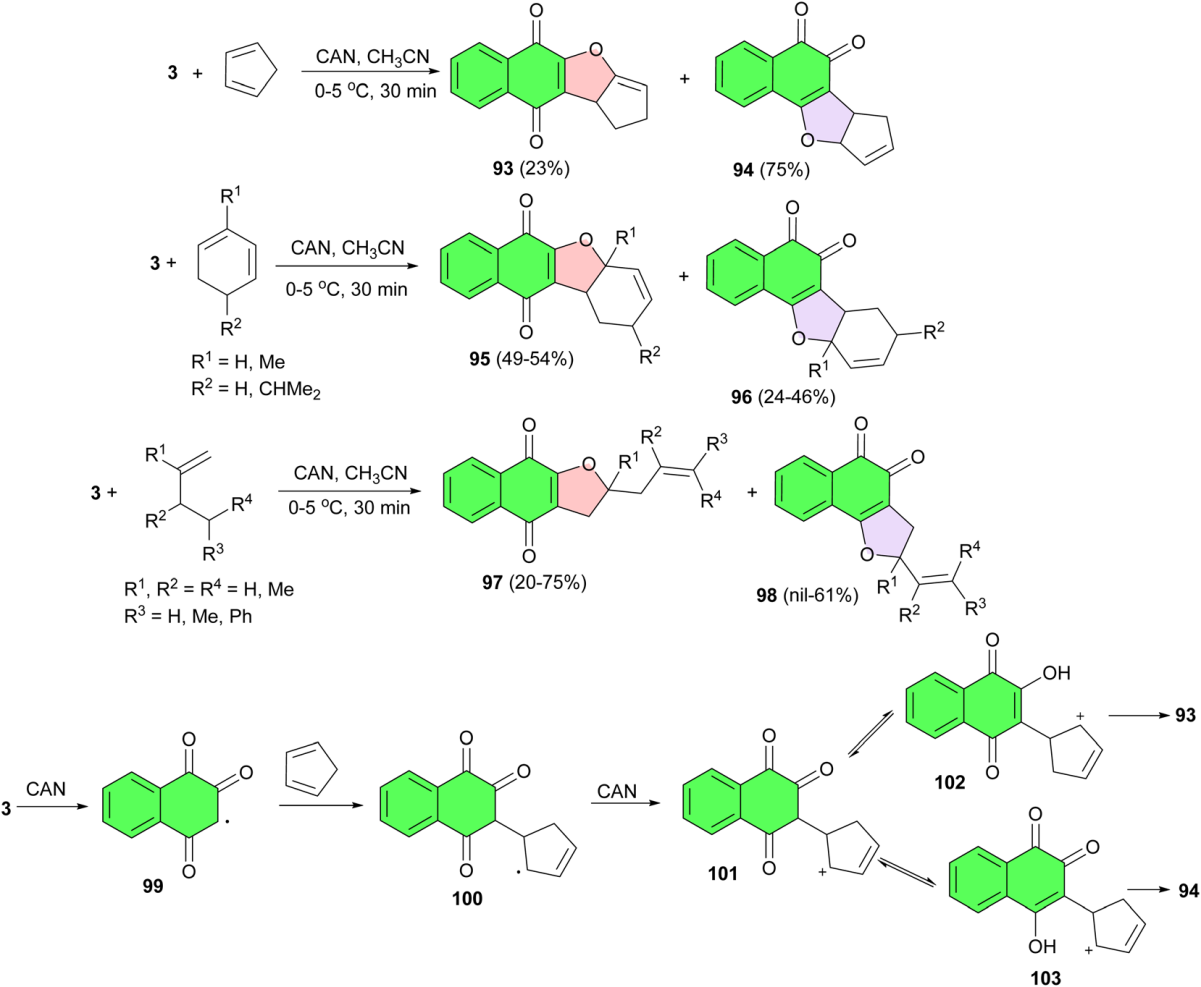
CAN mediated synthesis of linear and angular naphthofurandiones 93–98.

Lawsone undergoes can mediated oxidative addition to various dienes followed by ring closure in CH_3_CN at 0–5 °C for 30 min yielding linear and angular naphthofurandiones 93–98 in 20–75% yields. The proposed mechanism is outlined in Scheme 27. Oxidation of lawsone by CAN lead to the radical 99, which is trapped by the diene to yield the reactive intermediate 100. The latter further oxidized by CAN to the cation 101, which in turn undergoes rearrangement yielding 102 and 103. The cyclization of 102 leads to 93 whereas 103 affords 94.^55^

**Scheme 28:**
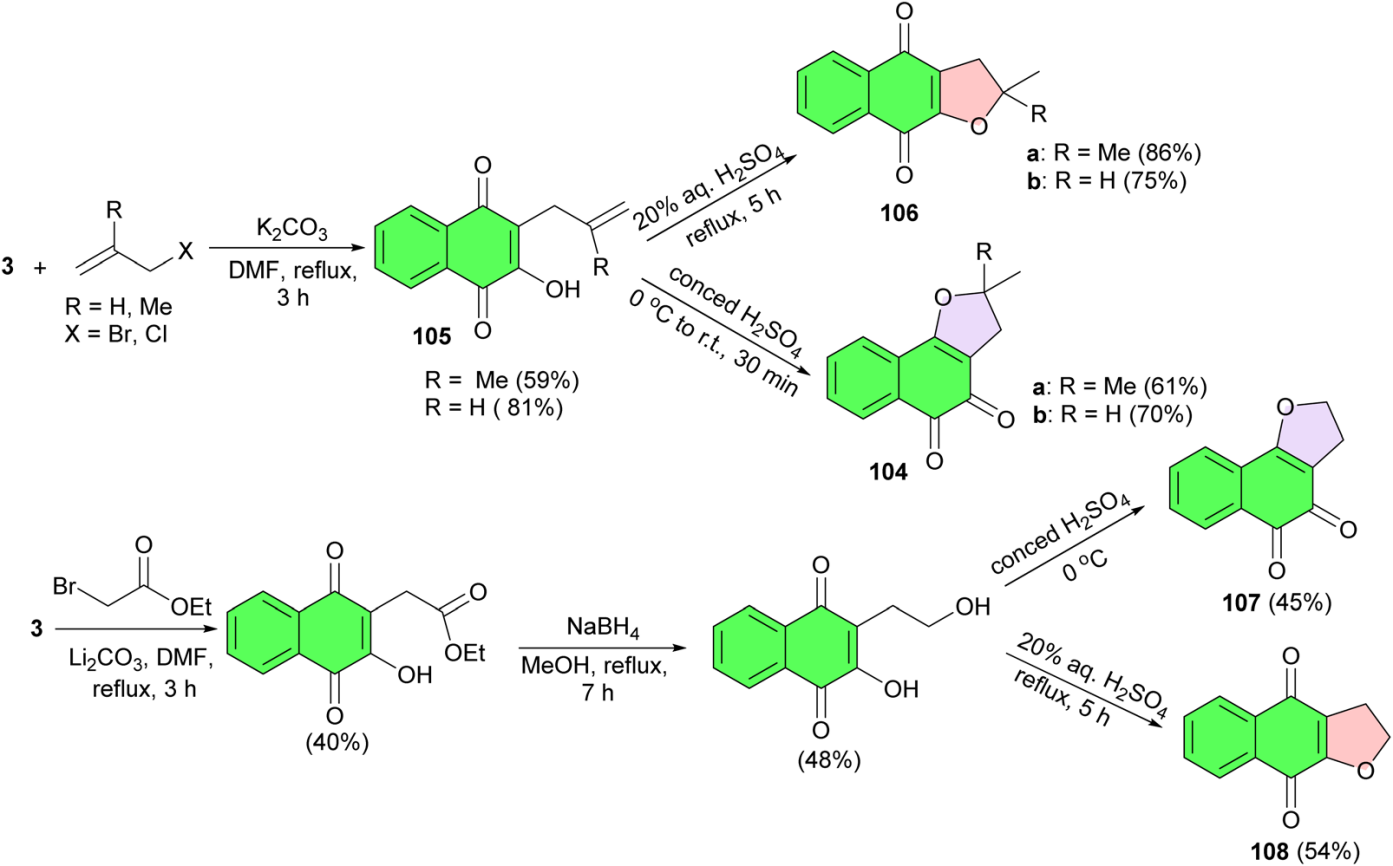
Synthesis of dihydrofuranonaphthoquinones 104, 106–108.

Dihydrofuranonaphthoquinones 104 were synthesized in only two steps (C-allylation and cyclization) from lawsone which could be isolated from the shrub, *Lawsonia inermis*. C-Allylation of lawsone using K_2_CO_3_ in DMF under reflux conditions for 3 h gave lawsone derivatives 105 in 59 and 81% yields. Compounds 105 were cyclized to furano-1,2-naphthoquinone 104a–b by concentrated sulfuric acid at 0 °C to room temperature for 30 min in 61 and 70% yields. With respect to cyclization of olefinic alcohols 105, using 20% aqueous sulfuric acid under reflux conditions for 5 h, it was found that the 1,4-naphthoquinone products 106a–b were obtained in 75 and 86% yields. It seemed that the mechanism of using concentrated sulfuric acid is different from using aqueous sulfuric acid. Also, furanonaphthoquinones 107 and 108 were accessed in 45 and 54% yields from C-alkylation of lawsone by α-bromoacetate ethyl ester followed by reduction with NaBH_4_ in MeOH and then cyclization by sulfuric acid. The mechanism of concentrated H_2_SO_4_ action may be mediated by tautomerization of protonated naphthoquinone whereas using aqueous H_2_SO_4_, protonation of the double bond at the side chain may be faster than protonation of the carbonyl group of naphthoquinone and then the hydroxy group at C-3 position attacked the carbocation straight away (Scheme 28). Compound 108 had significant cytotoxicity against HeLa cancer cell line (IC_50_ value of 9.25 mM) while it showed no toxic to vero cell.^56^

**Scheme 29:**
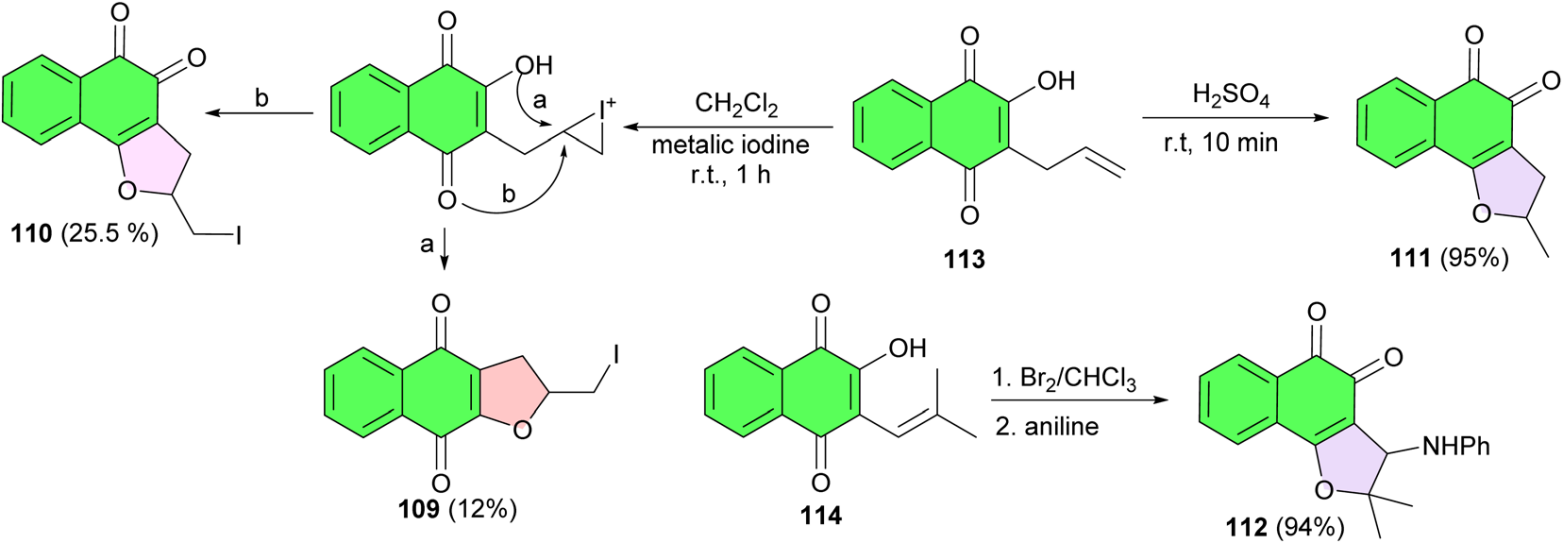
Synthesis of naphthofuranquinones 109–112 with activity against *Trypanosoma cruzi*.

In 2006, The de Castro group disclosed four naphthofuranquinones 109–112, obtained from 2-hydroxy-3-allyl-naphthoquinone (113) and nor-lapachol (114) and their structures established by physical and X-ray analysis. Compounds 109 and 110 were obtained by addition of iodine to 113 followed by cyclization generating a furan ring and separated by column chromatography. Compound 111 was obtained through the acid-catalyzed reaction by dissolution of 113 in sulfuric acid. Compound 112 was synthesized by addition of bromine and aniline to 114. The IC_50_/24 h for 109–112 in assays with *T. cruzi* trypomastigotes was between 157 and 640 μM, while the value for crystal violet was 536.0 ± 3.0 μM. Compounds 109–111 also inhibited epimastigote proliferation (Scheme 29).^57^

**Scheme 30:**
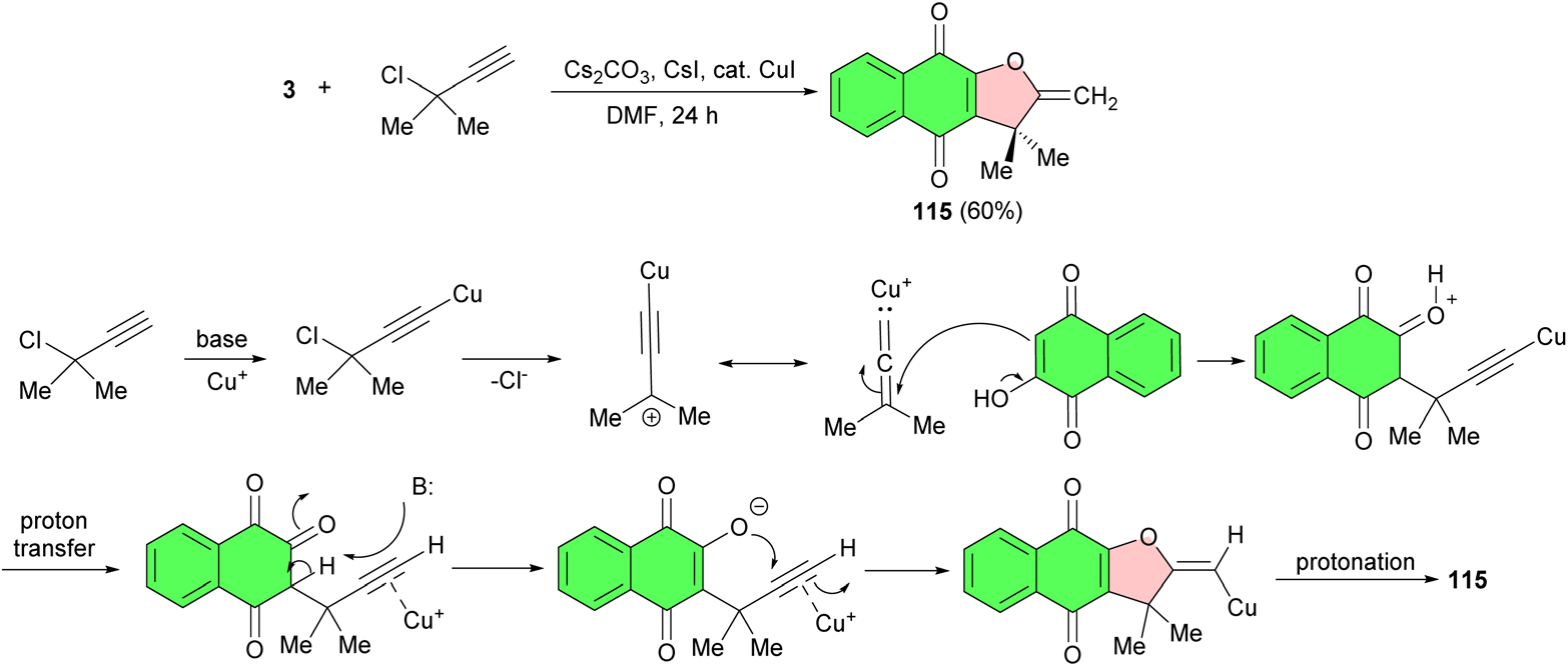
Synthesis of dehydro-α-dunnione 115.

The Cooke group reported synthesis of dehydro-α-dunnione 115 in 60% yield by the reaction of lawsone with 3-chloro-3-methyl-1-butyne *via* one-pot, formal [3 + 2] cyclization. The reaction was carried out in the presence of CuI as catalyst in DMF as solvent for 24 h. The reduction of the exocyclic double bond in compound 115 for the synthesis of α-dunnione, using Pd/C and pressurized H_2_ gas in MeOH, showed no progress even after several days or weeks. A plausible mechanism is illustrated in Scheme 30. They suggested that under the conditions of the coupling, a zwitterion-vinyl carbene intermediate is formed. This species couples with lawsone at C-3. Proton transfer (intramolecular or intermolecular) provides the terminal alkyne, π-activated by the copper ions present. Cyclization by the nucleophilic oxygen at C-2 on the activated alkyne thus forms the exocyclic enol vinylcuprate, which is protonated *in situ* or during the aqueous work-up.^58^

**Scheme 31:**
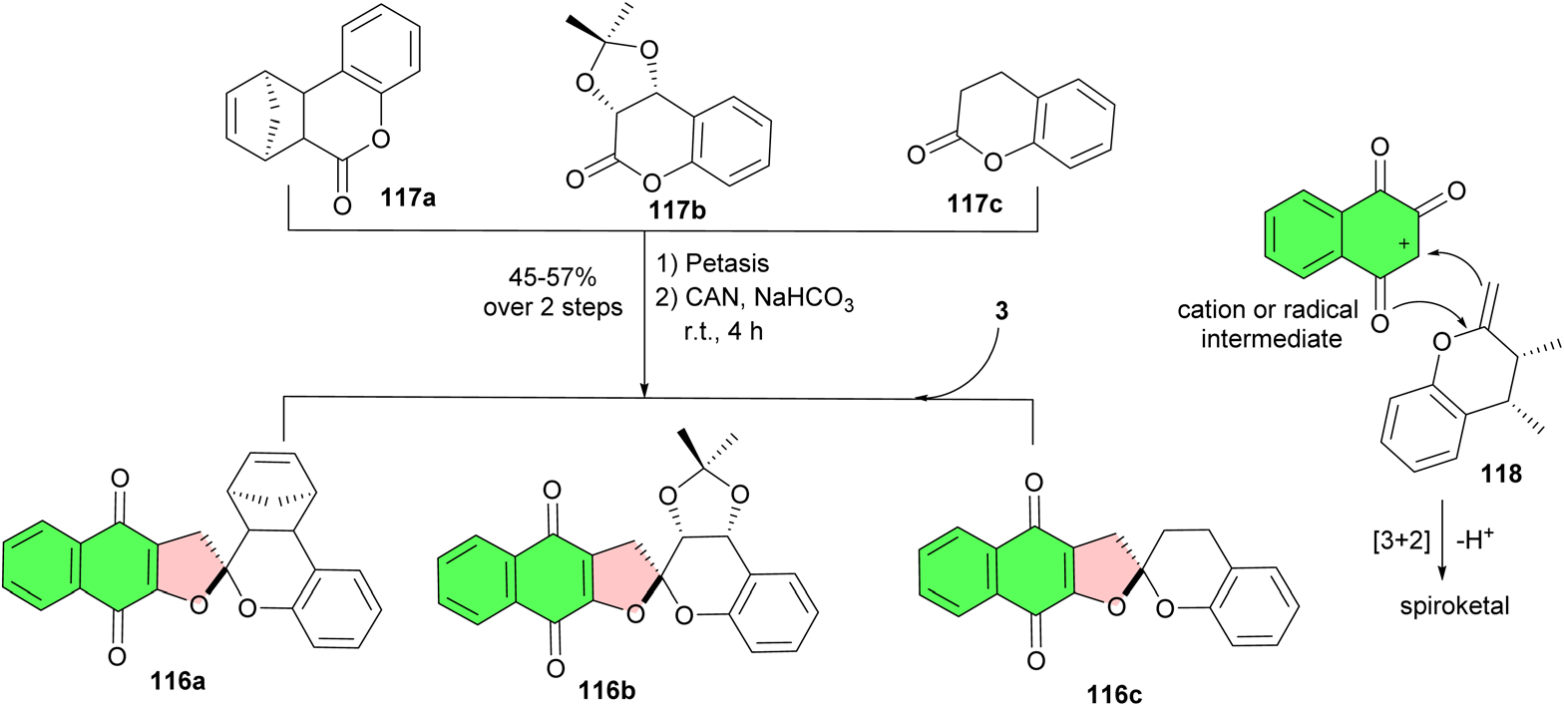
Synthesis of naphthoquinone spiroketals 116 by diastereoselective oxidative [3 + 2] cycloaddition.

Reich and co-workers described a highly selective oxidative [3 + 2] cycloaddition of chiral enol ethers and hydroxynaphthoquinone. This convergent strategy is amenable to an enantioselective synthesis of naphthoquinone spiroketals 116a–c. In this reaction, initially, the carbonyl functionality in chromenone compounds 117a–c was individually subjected to methylenation using the Petasis reagent. After purification, the corresponding exocyclic enol ether 118 was subjected to oxidation by cerium ammonium nitrate in the presence of the 2-hydroxy-1,4-naphthoquinone (Scheme 31). Naphthoquinone spiroketal 116a was found to inhibit DNA-polymerase and telomerase in a manner resembling α-rubromycin and β-rubromycin.^59^

**Scheme 32:**
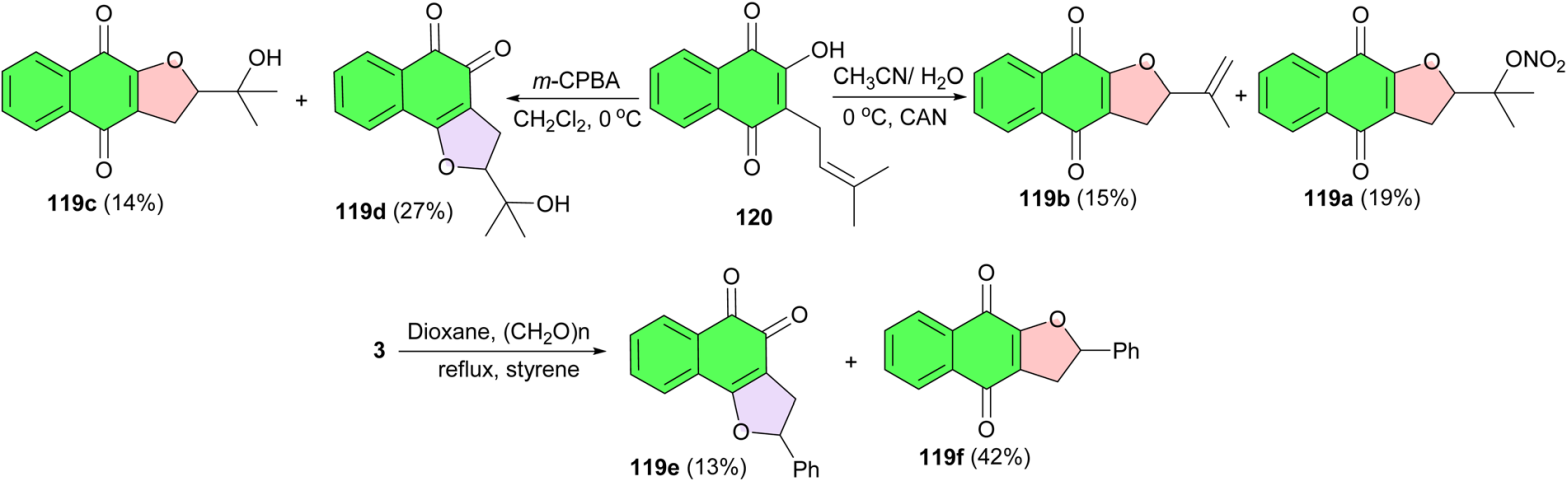
Synthesis of dihydronaphthofuranquinone derivatives 119a–f.

The Estevez-Braun group reported synthesis of naphthofuranquinone derivatives 119a–f from lapachol (120) or lawsone under various conditions (Scheme 32). The synthesized naphthoquinone derivatives tested in human promyelocytic leukemia HL-60 cell line and the computational models have facilitated the identification of structural elements of the ligands that are key for antitumoral properties. The results of the study provided a valuable tool in designing new and more potent cytotoxic analogues.^60^

**Scheme 33:**
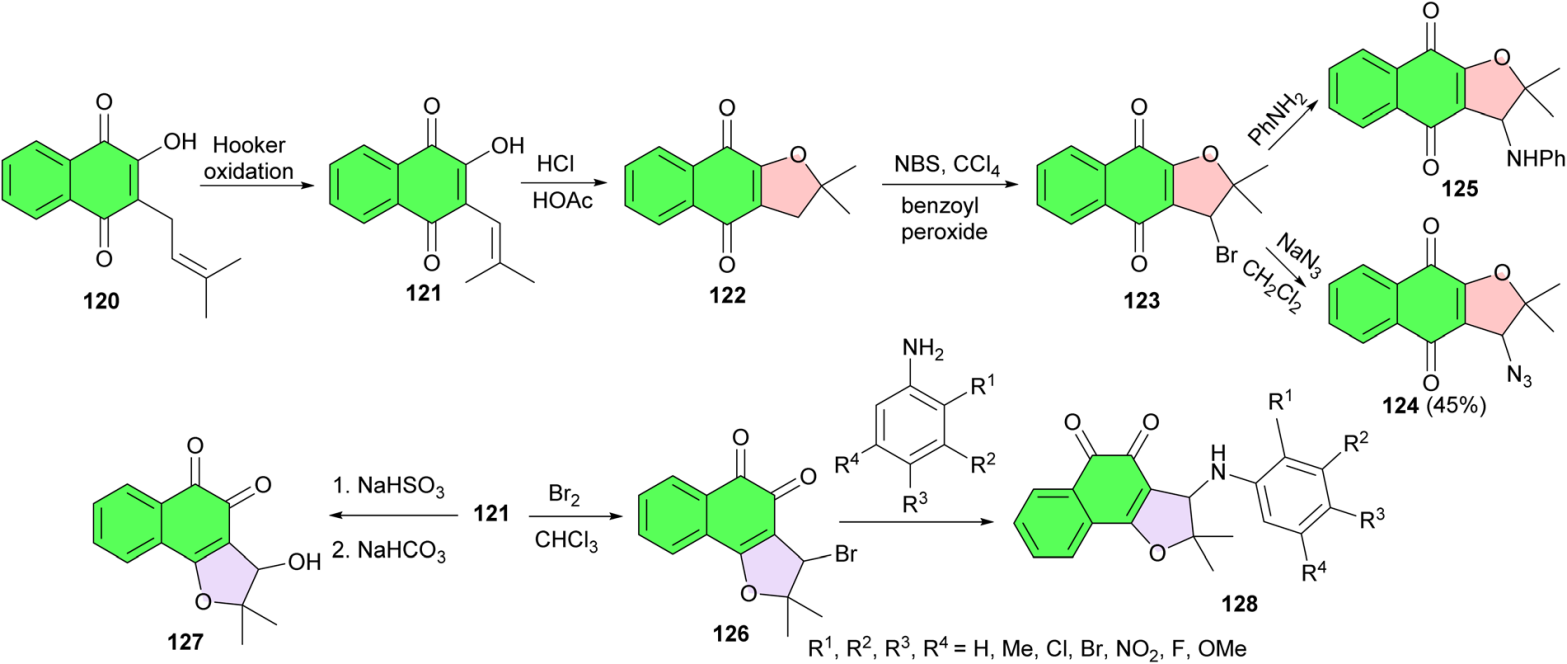
Synthetic route for preparing the naphthoquinones 122–128 assayed against trypomastigote forms of *T. cruzi*.

In 2008, the de Castro group reported synthesis of some naphthofuranquinone derivatives from nor-lapachol. Nor-lapachol (2-hydroxy-3-(2-methyl-propenyl)-[1,4]-naphthoquinone) 121 was obtained from lapachol (2-hydroxy-3-(3′-methyl-2-butenyl)-1,4-naphthoquinone) (120) by Hooker oxidation.^61^ The treatment of 121 with HCl/AcOH produced nor-α-lapachone 122 which was transformed into 3-bromo-nor-a-lapachone 123. Reaction of 123 with sodium azide in CH_2_Cl_2_ and with aniline gave, respectively, the corresponding azide and arylamino derivatives 124 and 125. The reaction of 121 with bromine in CHCl_3_ originated 126, the starting material for the synthesis of the naphthoquinone 127 and of the *ortho*-arylaminonaphthofuranquinones 128. The compounds were rationalized based on hybrid drugs and appear as important compounds against *Trypanosoma cruzi* (Scheme 33).^62^

**Scheme 34:**
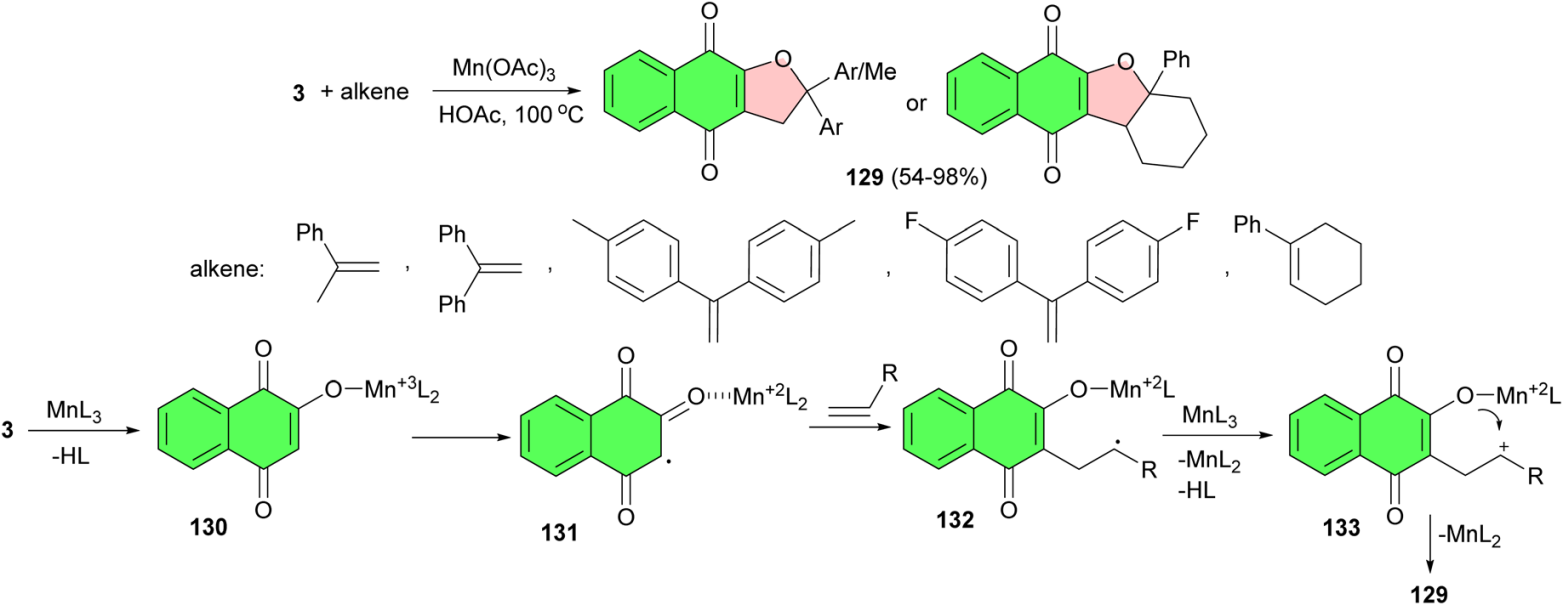
Mn(OAc)_3_ catalyzed synthesis of 2,3-dihydronaphtho[2,3-*b*]furan-4,9-diones 129.

Next, the Yılmaz group reported synthesis of 2,3-dihydronaphtho[2,3-*b*]furan-4,9-diones 129 in 54–98% yields in different molar ratios by the radical cyclizations of 2-hydroxy-1,4-naphtaquinone with electron-rich alkenes in the presence of manganese(iii) acetate under nitrogen in HOAc at 100 °C. According to the proposed mechanism, Mn(OAc)_3_ (MnL_3_) and hydroxyenone give manganese(iii)-enolate complex 130, and an α-carbon radical 131 is formed on this structure while Mn^3+^ is reduced to Mn^2+^. A radical intermediate product 132 is obtained in the addition of the α-carbon radical to alkene. This product is oxidized to carbocation 133 with equivalent of Mn(OAc)_3_. The intramolecular cyclization of 133 can produce linear product 129 (Scheme 34).^63^

**Scheme 35:**
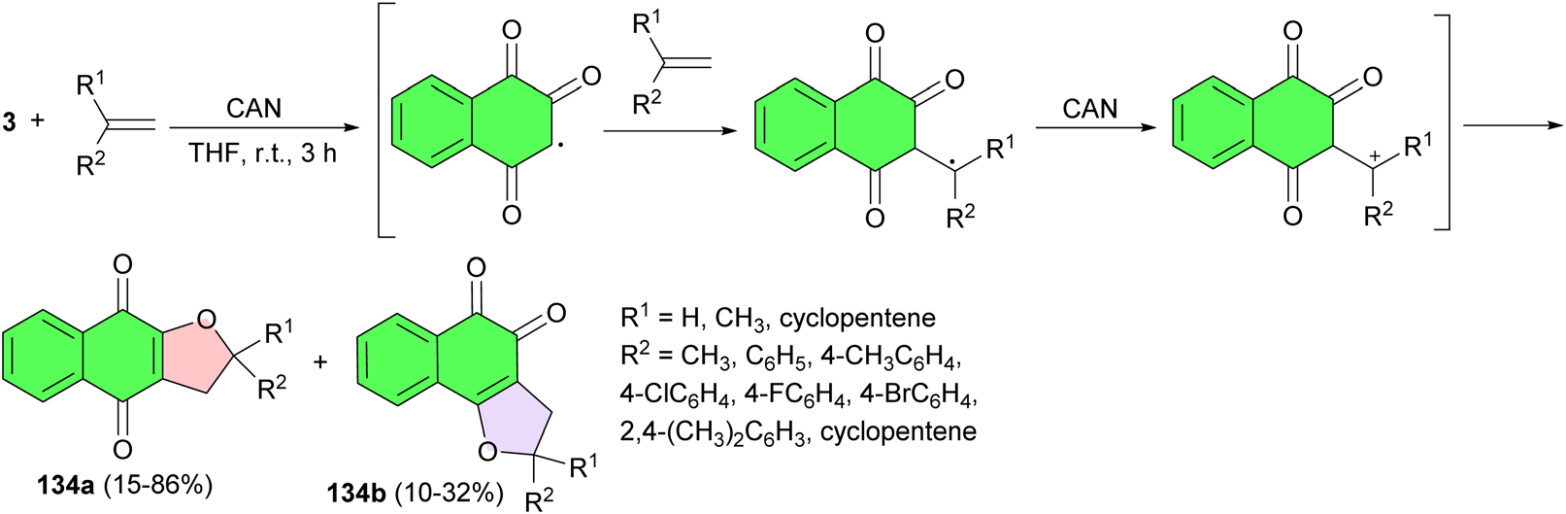
CAN catalyzed synthetic route used for the preparation of α- and β-furan naphthoquinones 134a–b.

In 2010, the Ferreira group described synthesis and antifungal activity of substituted α- and β-dihydrofuran naphthoquinones 134a–b. The furan naphthoquinones were obtained in 10–86% yields by oxidative [3 + 2] cycloaddition of lawsone, to the alkene, mediated by cerium(iv) ammonium nitrate (CAN) in THF at room temperature for 3 h (Scheme 35). It is noteworthy that all the products were formed regioselectively, with respect to the double bond of the alkene. The compounds evaluated against the following six strains of *Candida* (*C*): *C. albicans*, *C. krusei*, *C. parapsilosis*, *C. kefyr*, *C. tropicalis* and *C. dubliniensis*. Some of the α-furan naphthoquinones exhibited potent antifungal activity, with no hemolytic activity or cytotoxic effects.^64^

**Scheme 36:**
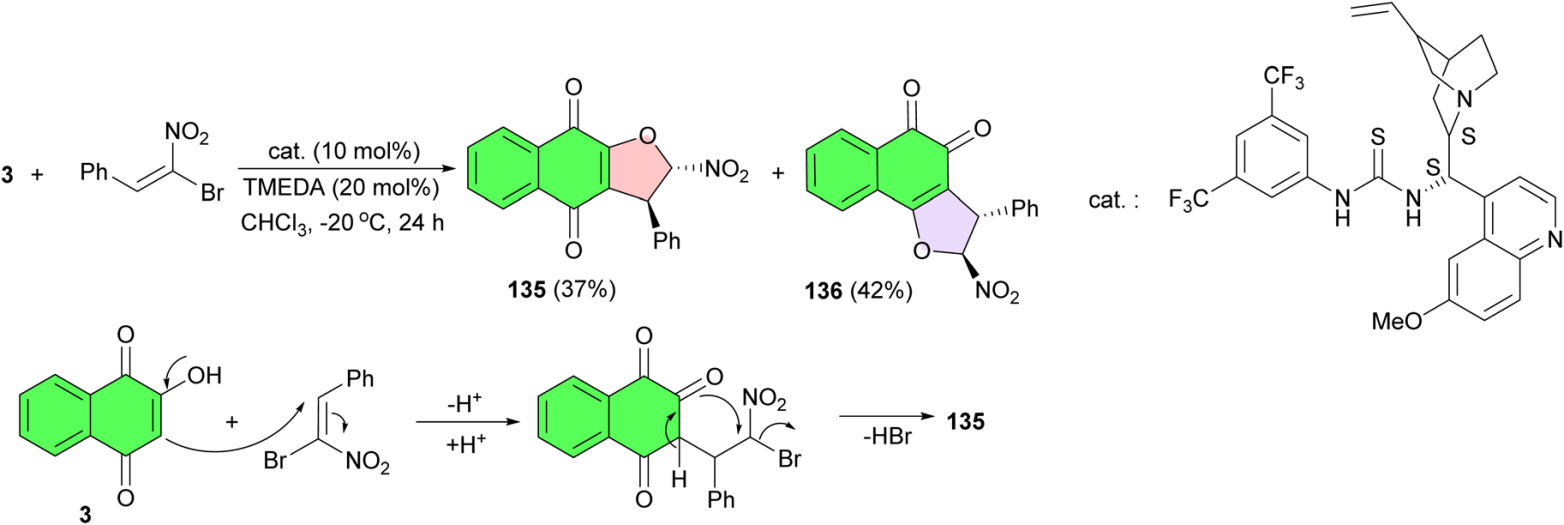
Enantioselective synthesis of dihydrofuranonaphthoquinones 135–136.

The Rueping group developed synthesis of dihydrofuranonaphthoquinones 135 and 136 as the two regioisomers in 37 and 42% yields and with excellent enantioselectivies of 90 and 92% ee, respectively. The reaction was carried out between lawsone and β,β-bromonitrostyrene in the presence of a chiral bifunctional thiourea catalyst in CHCl_3_ at −20 °C for 24 h. The proposed reaction sequence would involve the enantiocontrolled Michael addition of lawsone to the (*E*)-β,β-bromonitrostyrene, followed by the diastereoselective cyclization requiring nucleophilic substitution of the bromide to yield the desired polysubstituted dihydrofurans (Scheme 36).^65^

**Scheme 37:**
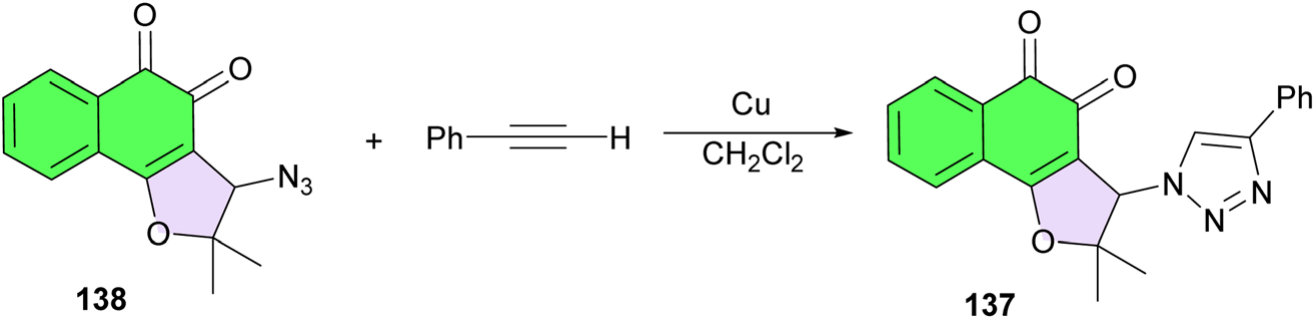
Synthesis of triazolic naphthofuroquinone 137.

In 2012, Menna-Barreto reported the mechanism of action of the triazolic naphthoquinone (TN; 2,2-dimethyl-3-(4-phenyl-1*H*-1,2,3-triazol-1-yl)-2,3-dihydronaphtho[1,2-*b*]furan-4,5-dione) (137), which is the most active compound against *T. cruzi* trypomastigotes among a series of naphthofuranquinones. It was synthesized by the reaction between 3-azido-nor-β-lapachone 138 and ethynylbenzene catalysed by Cu. The key intermediate, azidoquinone, was generated by nucleophilic substitution from 3-bromo-β-norlapachone with sodium azide in dichloromethane (Scheme 37).^66^

**Scheme 38:**
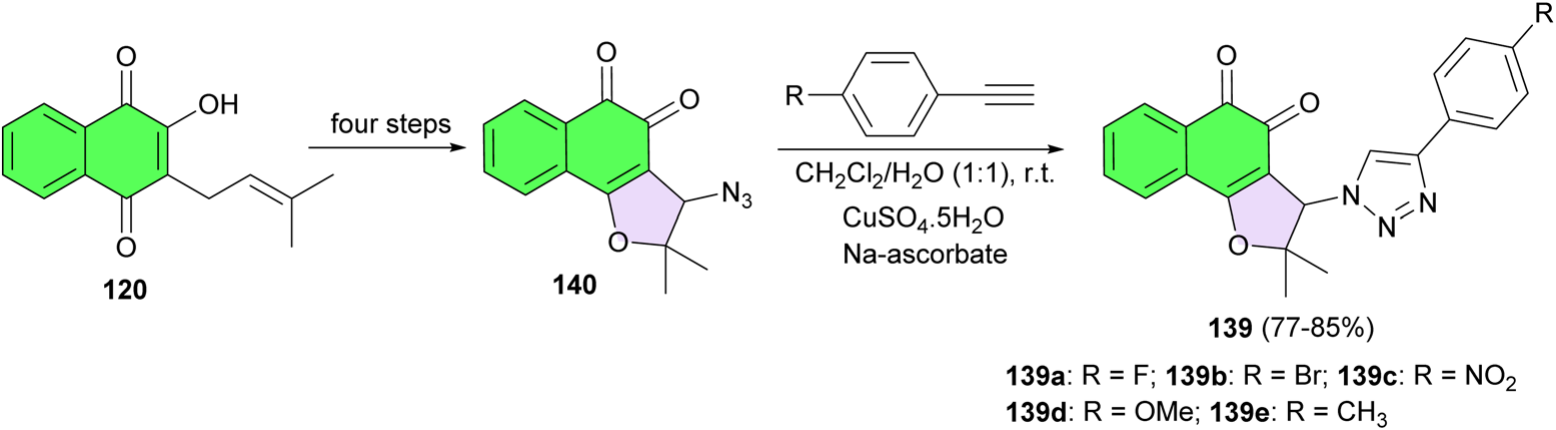
Synthesis of nor-β-lapachone-based 1,2,3-triazoles 139.

Next, synthesis of nor-β-lapachone-based 1,2,3-triazoles 139 reported in 77–85% yields by the reaction of substituted alkyne with 3-azido-2,2-dimethyl-2,3-dihydronaphtho[1,2-*b*]furan-4,5-dione 140 in the presence of CuSO_4_·5H_2_O and sodium ascorbate in CH_2_Cl_2_/H_2_O at room temperature. Compound 140 was previously synthesized from lapachol in a four-step process.^67^ These compounds were evaluated against the infective bloodstream form *Trypanosoma cruzi*, the etiological agent of Chagas disease. All of the compounds were considered potent trypanocidal compounds, with the exception of 139d (IC_50_/24 h ¼ 359.2 11.1 mM). The triazoles 139a, 139c and 139e were 2.5–5 times more active than the standard drug and represent potential candidates for further testing in preclinical assays of drugs for the treatment of Chagas disease. The insertion of electron withdrawing groups possibly amplifies the redox potential of the naphthoquinoidal structure, leading to an important increase in its biological activity (Scheme 38).^68^

**Scheme 39:**
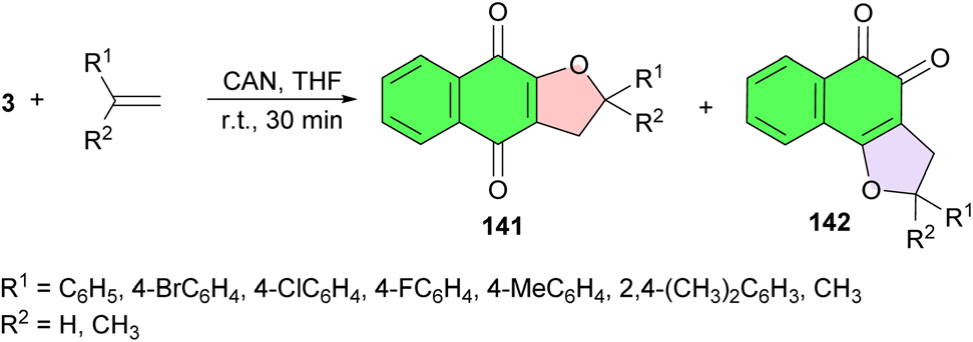
Synthetic route used for the preparation of α- and β-dihydrofuran naphthoquinones 141–142.

Next, α- and β-dihydrofuran naphthoquinones 141–142, respectively, synthesized in good yields from readily available lawsone and olefins in the presence of cerium(iv) ammonium nitrate in THF at room temperature for 30 min. The reaction led to two products: the α-and β-dihydrofuran naphthoquinones which were separated by column chromatography on silica gel (Scheme 39). The antitumor activity of the compounds against 4 human tumor cell lines, HL-60 (leukemia), SF-295 (CNS), HCT-8 (colon) and MDA-MB435 (melanoma), and their electrochemical parameters, in the absence and presence of oxygen were investigated in comparison with their non-substituted precursors. The β-dihydrofuran naphthoquinones were shown to be highly cytotoxic, while their α-isomers were shown to be less active.^69^

**Scheme 40:**
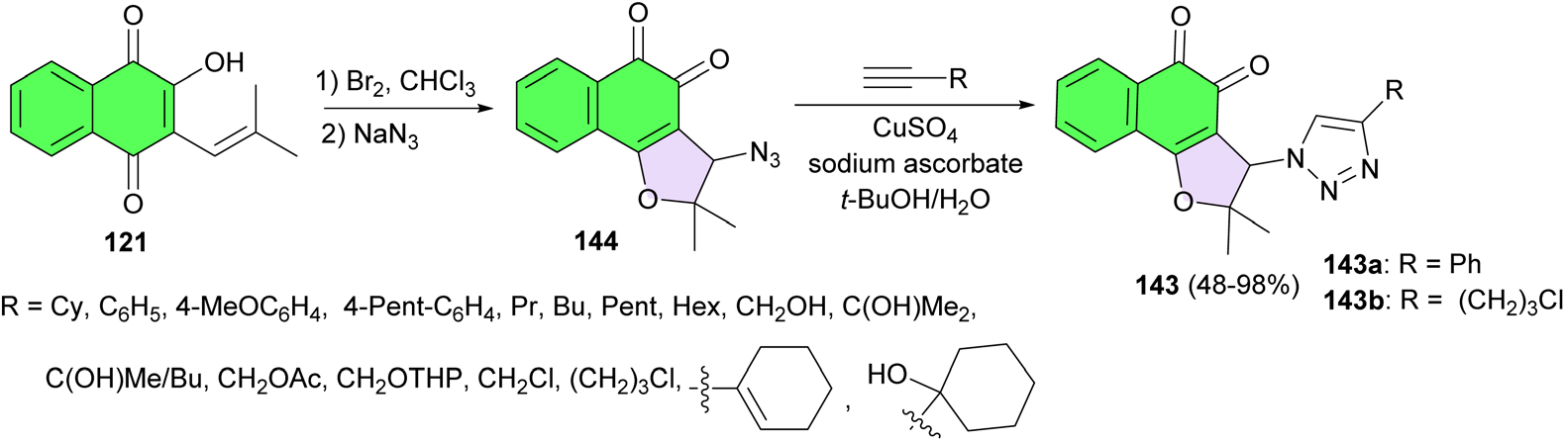
Preparation of 1,2-furanaphthoquinone triazoles 143.

In 2014, the da Silva group reported synthesis of 1,2-furanonaphthoquinones tethered to 1,2,3-1*H*-triazoles (1,2-FNQT) 143 and investigated their antileukemic activity. At first, nor-β-lapachol was reacted with excess of bromine in chloroform, followed by nucleophilic substitution with sodium azide in dichloromethane to yield 144 (3-azido-2,2-dimethyl-2,3-dihydronaphtho[1,2-*b*]furan-4,5-dione) in 90% yield. This key intermediate was employed in Huisgen 1,3-dipolar cyclization with an appropriate terminal alkyne catalyzed by copper(i) ion, also known as a click reaction, to obtain the desired product 143 in 48–98% yields. Compound 143a has great potential for further development as an anti-leukemia drug not only because of its potent and selective cytotoxicity (normal × cancer cells) but also because of its selectivity against leukemia lymphoid cell lines (approximately 7–19 times more effective than in leukemia myeloid cells). Additionally compound 143b is also promising due to its high cytotoxic activity against some leukemia cells (IC_50_ ranging from 0.48 to 1.38 μM) and its lower toxicity against normal hematopoietic cells (estimated IC_50_ > 10 μM) (Scheme 40).^70^

**Scheme 41:**
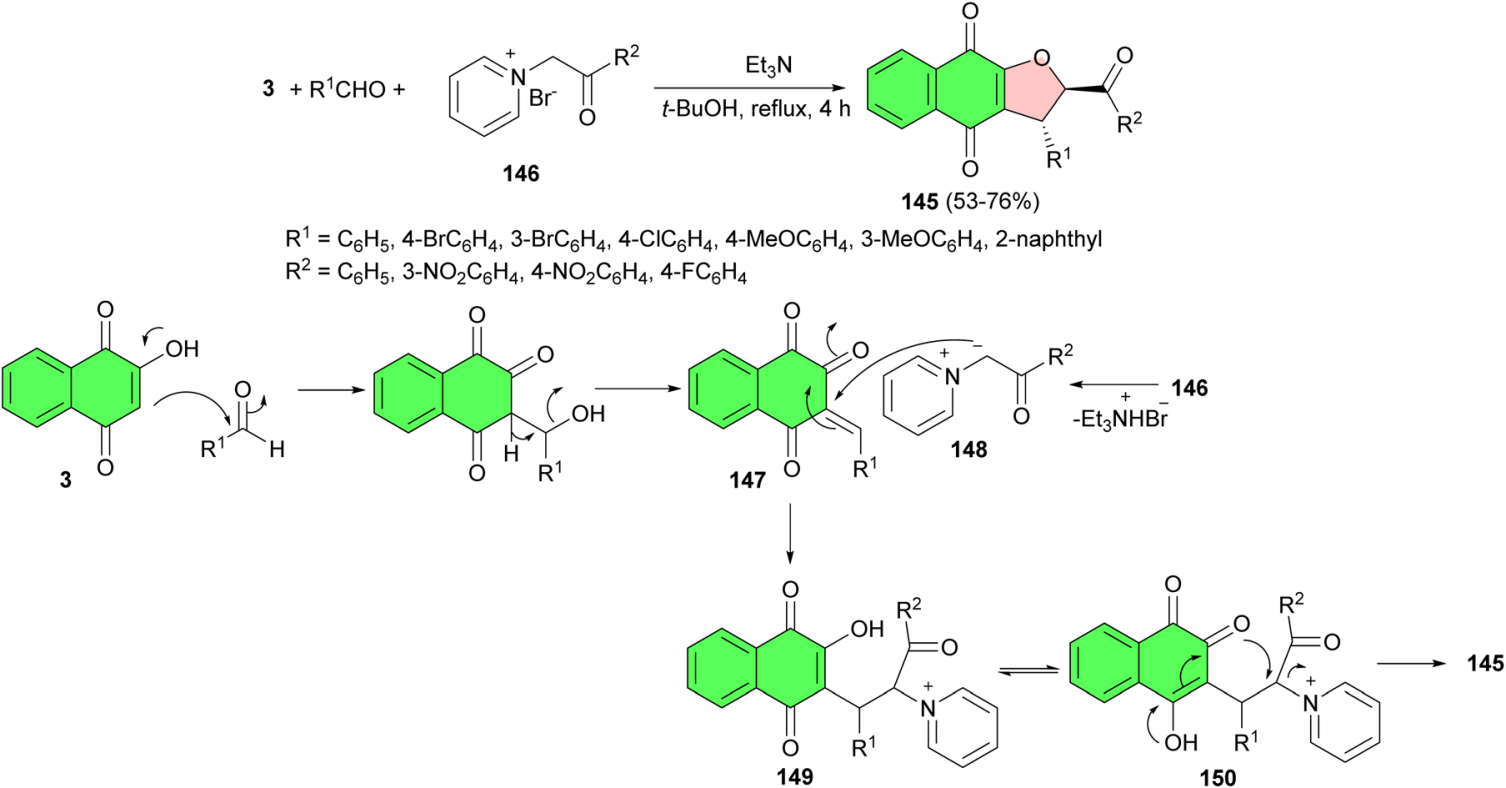
Stereoselective synthesis of dihydrofuranonaphthoquinones 145.

After that, Nguyen and co-workers explored an efficient stereoselective synthesis of dihydrofuranonaphthoquinones 145 in 53–76% yields by means of one-pot multicomponent reactions using lawsone, an aromatic aldehyde and pyridinium bromide 146 using Et_3_N in *t*-BuOH under reflux conditions for 4 h. A possible mechanistic explanation for this multicomponent reaction starts with a Knoevenagel condensation of lawsone with aromatic aldehydes, followed by dehydration resulting in the formation of 1,2,3,4-tetrahydro-1,2,4-naphthalenetriones 147. The next step is a Michael addition of pyridinium ylides 148, formed *in situ* by deprotonation of pyridinium bromides 146 by triethylamine, across Michael acceptors 147. The obtained naphthoquinones 149/150 undergo a cyclization to produce the desired substituted dihydrofuranonaphthoquinones 145 (Scheme 41).^71^

**Scheme 42:**
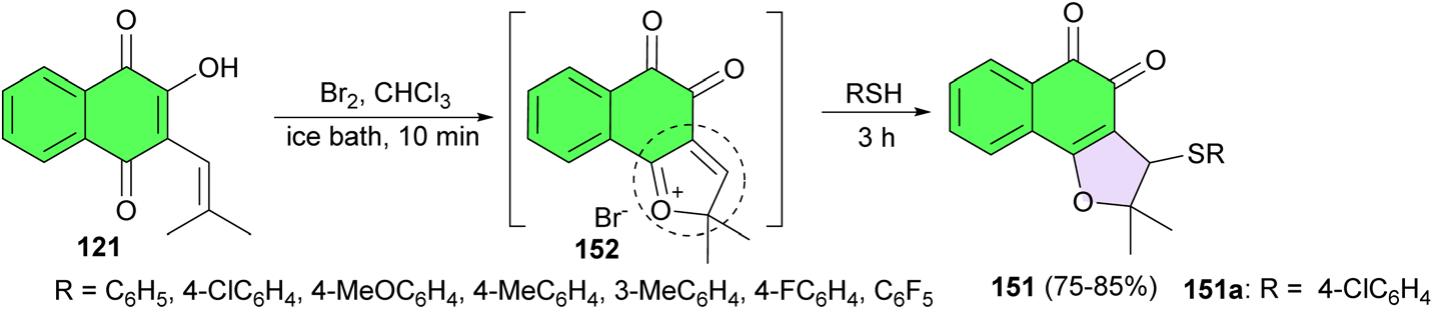
Synthesis of 3-phenylthio groups linked to 2,3-dihydronaphtho[1,2-*b*]-furan-4,5-diones 151.

Further, Ferreira and co-workers revealed an efficient route to prepare compounds containing 3-phenylthio groups linked to 2,3-dihydronaphtho[1,2-*b*]-furan-4,5-diones 151. This methodology involved one-step reactions starting with nor-lapachol 121, which upon reaction with bromine in chloroform generates *in situ* cationic *ortho*-quinone methide 152. This intermediate then reacts with phenyl thiols for 3 h to produce the corresponding product in 75–85% yields. The compounds possess a broad range of activity (IC_50_/24 h from 9.2 to 182.7 μM), higher than the original quinone (391.5 μM) and four of them higher than standard drug benznidazole (103.6 μM). The most active was compound 151a (9.2 μM), being 11 times active than benznidazole and the less toxic derivative to heart muscle cells (Scheme 42).^72^

**Scheme 43:**
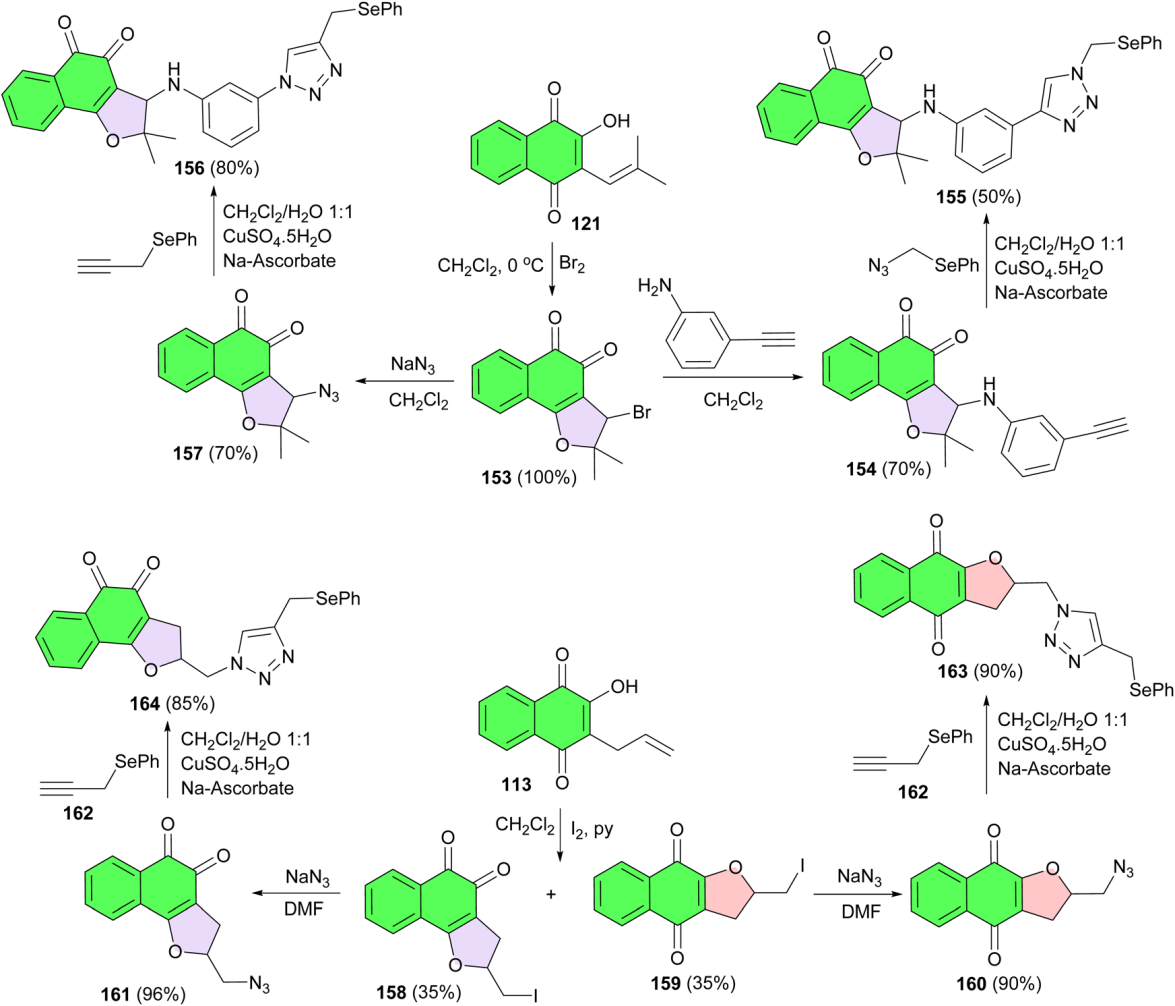
Synthesis of selenium-containing furanonaphthoquinones 155–156, 163–164.

Selenium-containing quinone-based 1,2,3-triazoles synthesized using click chemistry, the copper catalyzed azide–alkyne 1,3-dipolar cycloaddition, and evaluated against six types of cancer cell lines: HL-60, HCT-116, PC3, SF295, MDA-MB-435 and OVCAR-8. These compounds could provide promising new lead derivatives for more potent anticancer drug development and delivery. For the preparation of these compounds, bromo intermediate 153 was synthesized from nor-lapachol in CH_2_Cl_2_ at 0 °C. Subsequently, arylamino-substituted lapachone 154, featuring a terminal alkyne group, was prepared from 153 in 70% yield. The selenium-containing 1,2,3-triazole 155 was then obtained from 154 in 50% yield in the presence of CuSO_4_. Following the same strategy as outlined above, compound 156 was synthesized from azide derivative 157 in 80% yield. Compound 157 itself was prepared from 153 using sodium azide. The iodination of 113 affords compounds 158 and 159 in 68% yield and 1 : 1 ratio, which were easily separated by column chromatography. With these compounds in hand, the respective azide derivatives, compounds 160 and 161, were synthesized in 90 and 96% yields by the reaction of sodium azide in dimethylformamide, respectively. The respective selenium derivatives 162, compounds 163 and 164, were prepared in 90 and 85% yields by Cu-catalyzed azide–alkyne cycloaddition, respectively (Scheme 43).^73^

**Scheme 44:**
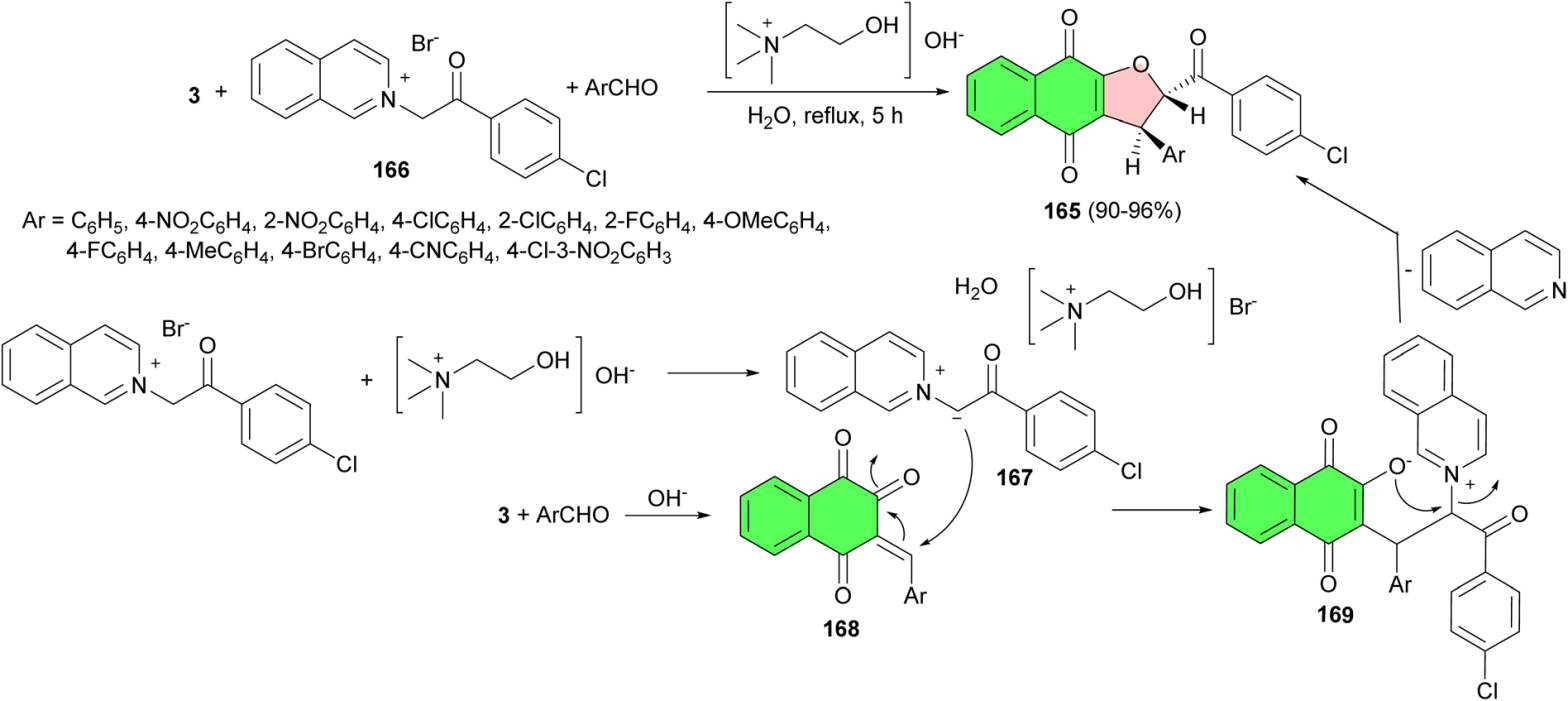
Choline hydroxide catalyzed synthesis of *trans*-2-(4-chlorobenzoyl)-3-aryl-2,3-dihydronaphtho[2,3-*b*]furan-4,9-diones 165.

Next, a green and highly efficient synthesis of *trans*-2-(4-chlorobenzoyl)-3-aryl-2,3-dihydronaphtho[2,3-*b*]furan-4,9-diones 165 achieved in 90–96% yields *via* a three-component, one-pot condensation of 2-[2-(4-chlorophenyl)-2-oxoethyl)]isoquinolinium bromide 166 with lawsone and an aromatic aldehyde in the presence of catalytic amounts of choline hydroxide in water under reflux conditions for 5 h. A proposed mechanism is shown in Scheme 44. Compound 166 undergoes deprotonation in the presence of aqueous choline hydroxide to give the reactive isoquinolinium ylide 167. Lawsone reacts with aromatic aldehyde in the presence of choline hydroxide to give the Knoevenagel product 168. This reacts instantly with the isoquinolinium ylide 167 to form the zwitterionic intermediate 169, which undergoes cyclisation with the elimination of isoquinoline to give the desired product 165.^74^

**Scheme 45:**
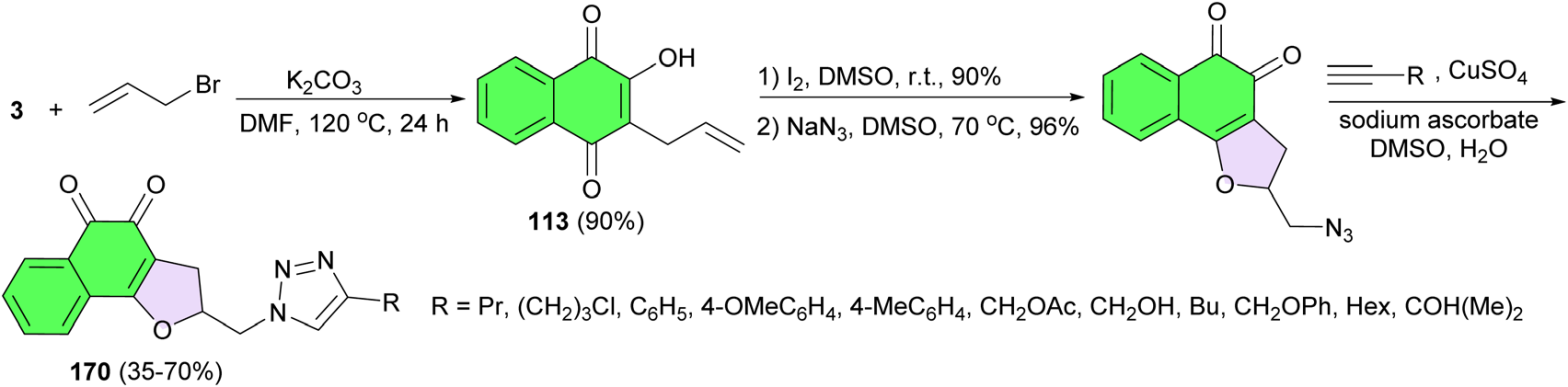
Synthesis of 1*H*-1,2,3-triazoles-linked to 2,3-dihydronaphtho[1,2-*b*]furan-4,5-dione 170.

Next, a series of 1,2-naphthoquinones tethered in C2 to 1,2,3-1*H*-triazoles 170 designed, synthesized and their cytotoxic activity evaluated using HCT-116 (colon adenocarcinoma), MCF-7 (breast adenocarcinoma) and RPE (human nontumor cell line from retinal epithelium). The chemical synthesis was performed from C-3 allylation of lawsone followed by iodocyclization with subsequent nucleophilic displacement with sodium azide and, finally, the 1,3-dipolar cycloaddition catalyzed by Cu(i) with terminal alkynes led to the formation of the desired product in 35–70% yields (Scheme 45). Compounds containing aromatic group linked to 1,2,3-triazole ring presented superior cytotoxic activity against cancer cell lines with IC_50_ in the range of 0.74 to 4.4 μM indicating that the presence of aromatic rings substituents in the 1,2,3-1*H*-triazole moiety is probably responsible for the improved cytotoxic activity.^75^

**Scheme 46:**
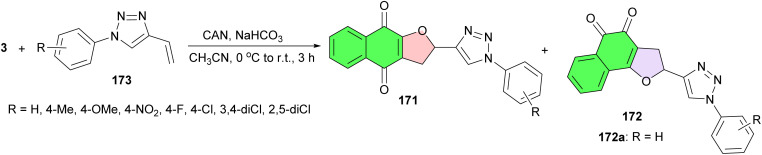
CAN mediated synthesis of furanaphthoquinones tethered to 1*H*-1,2,3-triazoles 171–172.

After that, Ferreira *et al.* revealed synthesis and evaluation of the cytotoxic activity of furanaphthoquinones tethered to 1*H*-1,2,3-triazoles 171–172 against human tumor cell lines (MDA-MB231, Calu-3 and Caco-2) and healthy cells (Vero). The reaction of lawsone with 4-vinyl-1*H*-1,2,3-triazoles 173 in the presence of CAN in acetonitrile at room temperature for 3 h. The isomers were isolated by column chromatography. The results showed that compound 172a exhibited the most promising profile due to its selective cytotoxic action against colonadenocarcinoma cells (Scheme 46).^76^

**Fig. 2:**
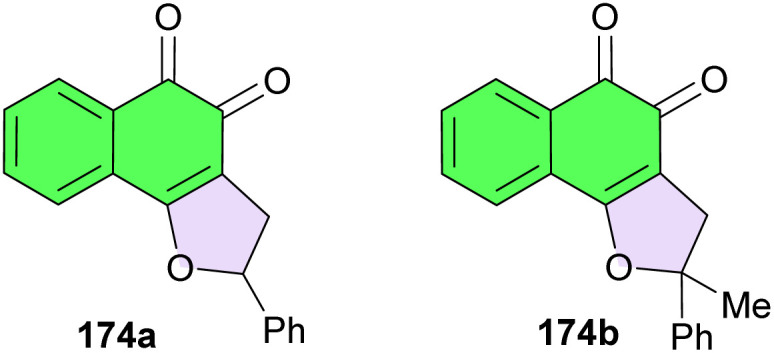
Structures of 2,3-dihydrofuranaphthoquinones 174a–b as antifungal.

Next, Ferreira *et al.* revealed antifungal activities of substituted α- and β-2,3-dihydrofuranaphthoquinones against *Sporothrix brasiliensis* and *Sporothrix schenckii*-the main etiological agents of sporotrichosis in Brazil. The results showed that compounds 174a and 174b were the most active dihydrofuranaphthoquinones *in vitro* for both species; in fungi, these compounds induced yeast-hyphae conversion and alteration in the hyphae and conidia structures. Compound 174b also exhibited a synergistic activity with itraconazole against *S. schenckii*, with a SFIC index value of 0.3 (Fig. 2).^77^

**Scheme 47:**
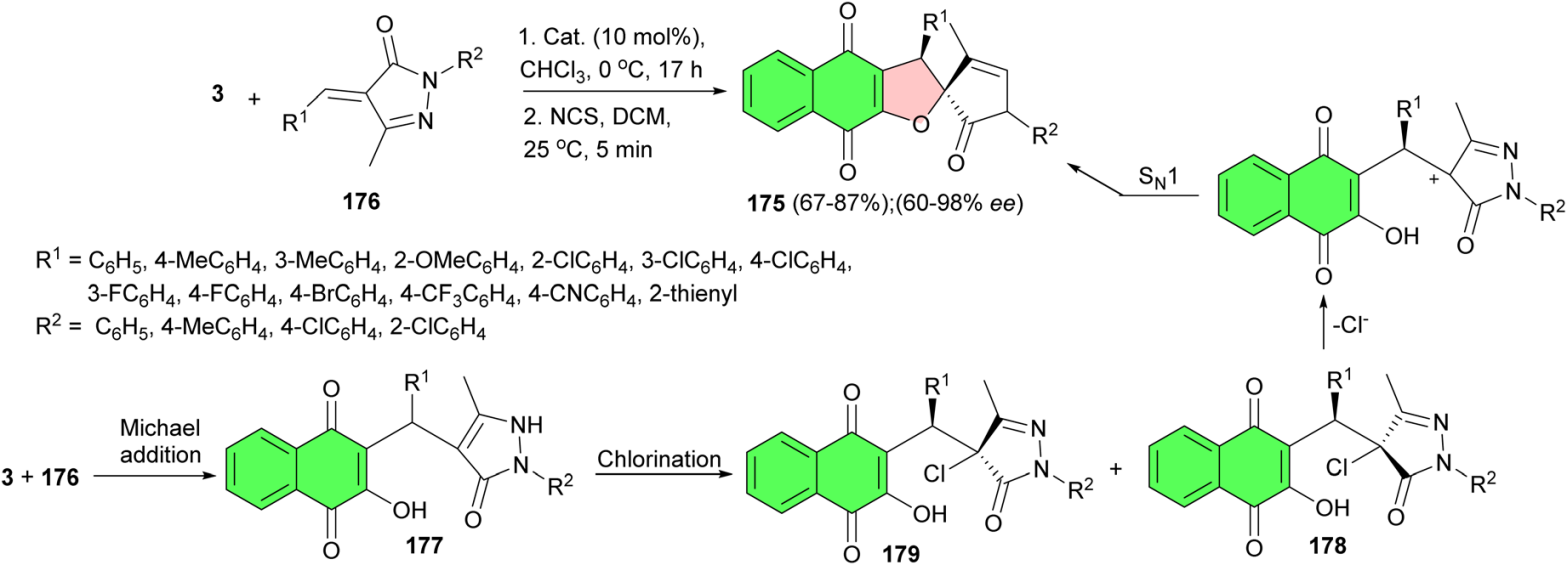
Asymmetric synthesis of spiropyrazolone-fused dihydrofurannaphthoquinones 175.

Next, the Song group revealed enantioselective synthesis of spiropyrazolone-fused dihydrofuran-naphthoquinones 175*via* a Michael addition/chlorination/nucleophilic substitution sequence. The reactions of lawsone and α,β-unsaturated pyrazolones 176 in the presence of the cinchona alkaloid derived hydrogen-bonding catalyst and NCS provide 175 bearing contiguous stereocenters in good to excellent yields (67–87%) and moderate to excellent enantioselectivities (60–98% ee). A plausible reaction mechanism is proposed in Scheme 47. Lawsone is deprotonated by the basic nitrogen atom of a tertiary amine to form a nucleophilic species, and the unsaturated pyrazolone 176 is synergistically activated by the squaramide moiety *via* two hydrogen bonds. Then Re-face attack occurs favorably in the Michael addition to give the enantioselective adduct 177. Computational study on the Hartree–Fock level revealed that chlorination of the Michael adduct 177 preferentially produces the thermodynamically stable product intermediate 178 rather than 179. The intermediate 178 then undergoes intramolecular nucleophilic substitution (S_N_1) to finally provide thermodynamically favored isomer 175 with exclusive diastereoselectivity and good enantioselectivity.^78^

**Scheme 48:**
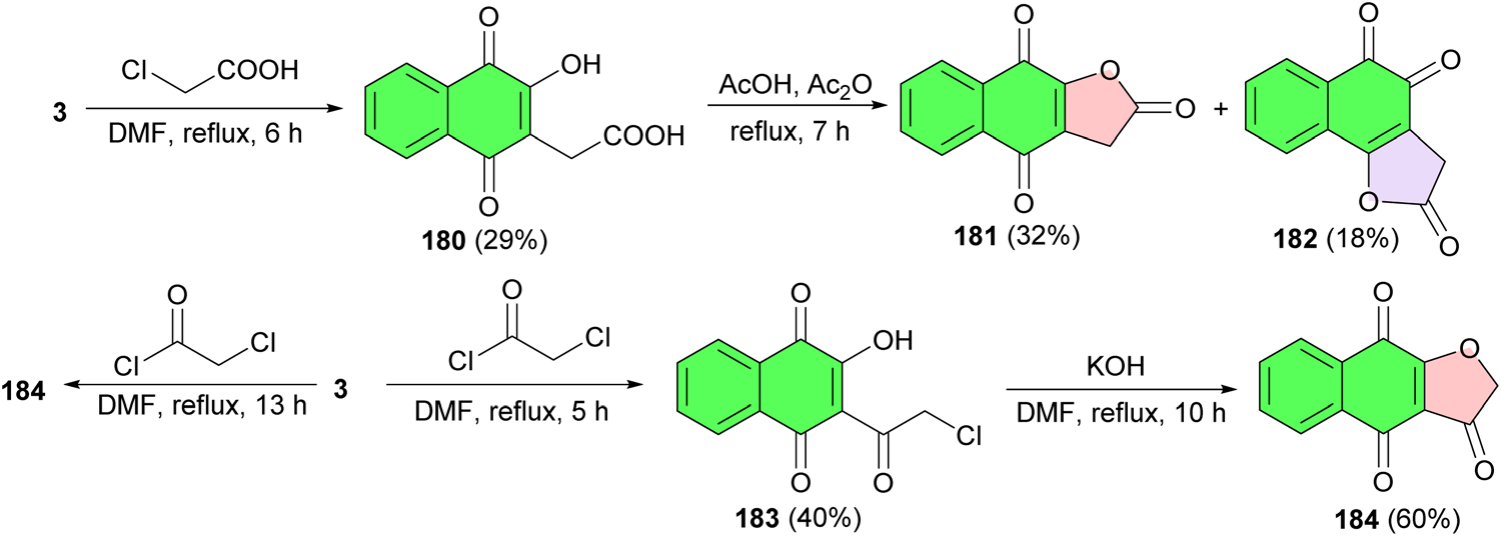
Synthesis of naphthofuranquinone derivatives 181–182, 184.

Further, Elbana and co-workers reported synthesis of 2-(3-hydroxy-1,4-dioxo-1,4-dihydronaphthalen-2-yl)acetic acid (180) by the rection of lawsone with chloroacetic acid in DMF under reflux conditions for 6 h. Then, the treatment of 180 with acetic acid and acetic anhydride under reflux for 7 h gave naphthofuran-2,4,9(3*H*)-triones 181 and 182. Moreover, the reaction of lawsone with chloroacetyl chloride in refluxing DMF for 5 h afforded 2-(2-chloroacetyl)-3-hydroxynaphthalene-1,4-dione (183). Also, naphtho[2,3-*b*]furan-3,4,9(2*H*)-trione (184) synthesized *via* two methods: refluxing a mixture of lawsone and chloroacetyl chloride in DMF for 13 h in the presence of the catalytic amount of potassium hydroxide and refluxing a mixture of 183 in DMF in the presence of a catalytic amount of potassium hydroxide for 10 h (Scheme 48). The target molecules were showed easy access to antioxidant and antitumor activities. Geometrical isomers (enol, Keto conformers, and *syn*, anti-conformers) were achieved by DFT that conformed to the spectral analysis of the investigated compounds.^79^

## 4. Synthesis of naphthofuroquinones together with their dihydro derivatives

**Scheme 49:**
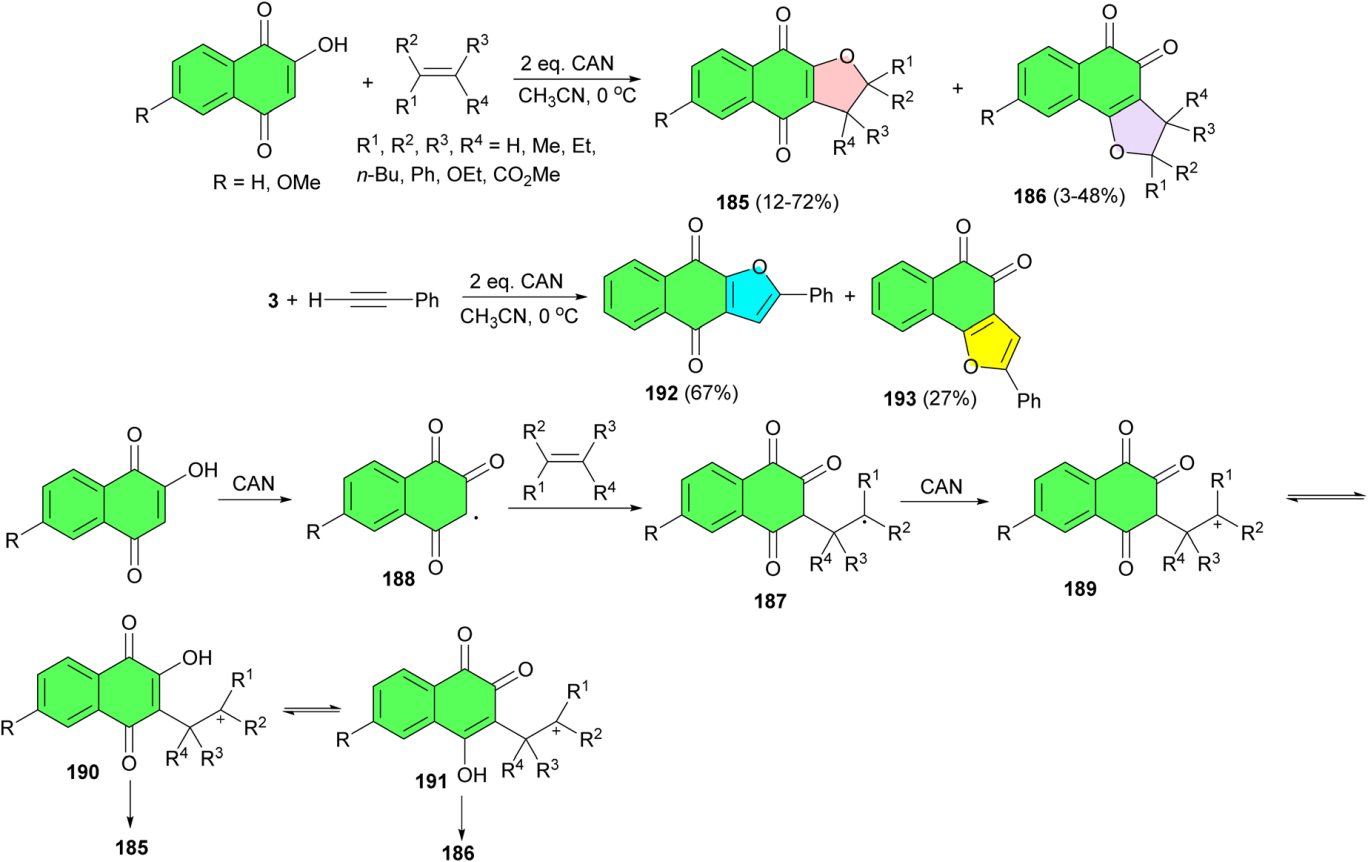
CAN mediated synthesis of furanonaphthoquinones 185–186, 192–193.

In 1996, the Kobayashi group reported a one-pot formation of 2,3-dihydronaphtho[2,3-*b*]furan-4,9-diones 185 in 12–72% yields and 2,3-dihydronaphtho[1,2-*b*]furan-4,5-diones 186 in 3–48% yields by the ceric ammonium nitrate (CAN) mediated [3 + 2] type cycloaddition of 2-hydroxy-1,4-naphthoquinones with alkenes or phenylacetylene in CH_3_CN at 0 °C. The proposed mechanism is outlined in Scheme 49. The formation of each product was completely regioselective. The initial formation of a reactive radical intermediate 187 (through 188) and its oxidation gives a carbonium ion intermediate 189, which is intramolecularly trapped with the hydroxyl group of 2-hydroxy-190 or 4-hydroxy-tautomer 191 to give 185 or 186, respectively. Analogous cycloaddition can be achieved with phenylacetylene instead of alkenes. Formation of the products 192 and 193 was almost quantitative.^80^

**Scheme 50:**
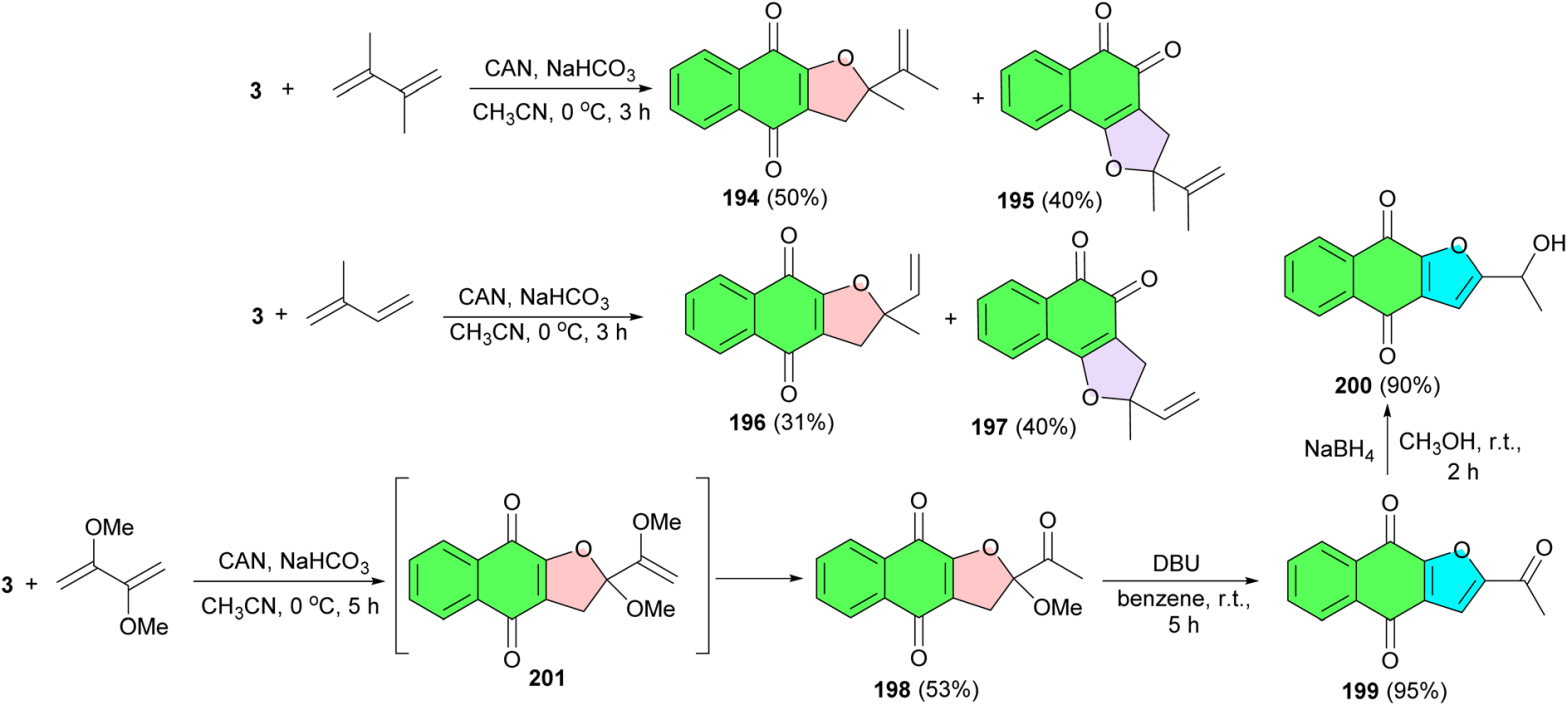
CAN-mediated synthesis of furonaphthoquinones 194–201.

In 2000, Lee and his group reported synthesis of dihydrofuronaphthoquinones 194 (50% yield) and 195 (40% yield) as a mixture of linear and angular regioisomers by the reaction of lawsone and conjugated dienes using ceric ammonium nitrate (CAN) in CH_3_CN at 0 °C for 3 h. The mixture was easily separated by column chromatography and the two isomers were assigned by their spectroscopic data. Similarly, with isoprene, two regioisomers, 196 and 197, were also obtained in 31 and 40% yields, respectively. Treatment of 3 with 2,3-dimethoxy-1,3-butadiene in the presence of 3.0 equiv. of CAN in acetonitrile at 0 °C for 5 h afforded the dihydrofuronaphthoquinone 198 in 53% yield. Reaction of 198 with DBU in benzene at room temperature for 5 h results in furonaphthoquinone 199 in 95% yield. Next, the synthesis of furonaphthoquinone 200 was easily achieved by reduction of 199 with sodium borohydride in methanol in 90% yield. The product 198 probably results from CAN-mediated oxidative cycloaddition and followed by the methyl group cleavage of intermediate 201 (Scheme 50).^81^ Compounds 199 and 200 are isolated from the *Tabebuia cassinoides* and they are reported to have significant biological properties such as antileukemic activity and *in vitro* cytotoxicity against KB, K562, and P388 cells.^82^ These furonaphthoquinone derivatives have been also used in traditional medicine as Pau d'Arco, IpeRoxo, Lapacho, and Taheebo for many years in North and South America as anticancer, antifungal, antibacterial, and antiinflammatory drugs.^83^

**Scheme 51:**
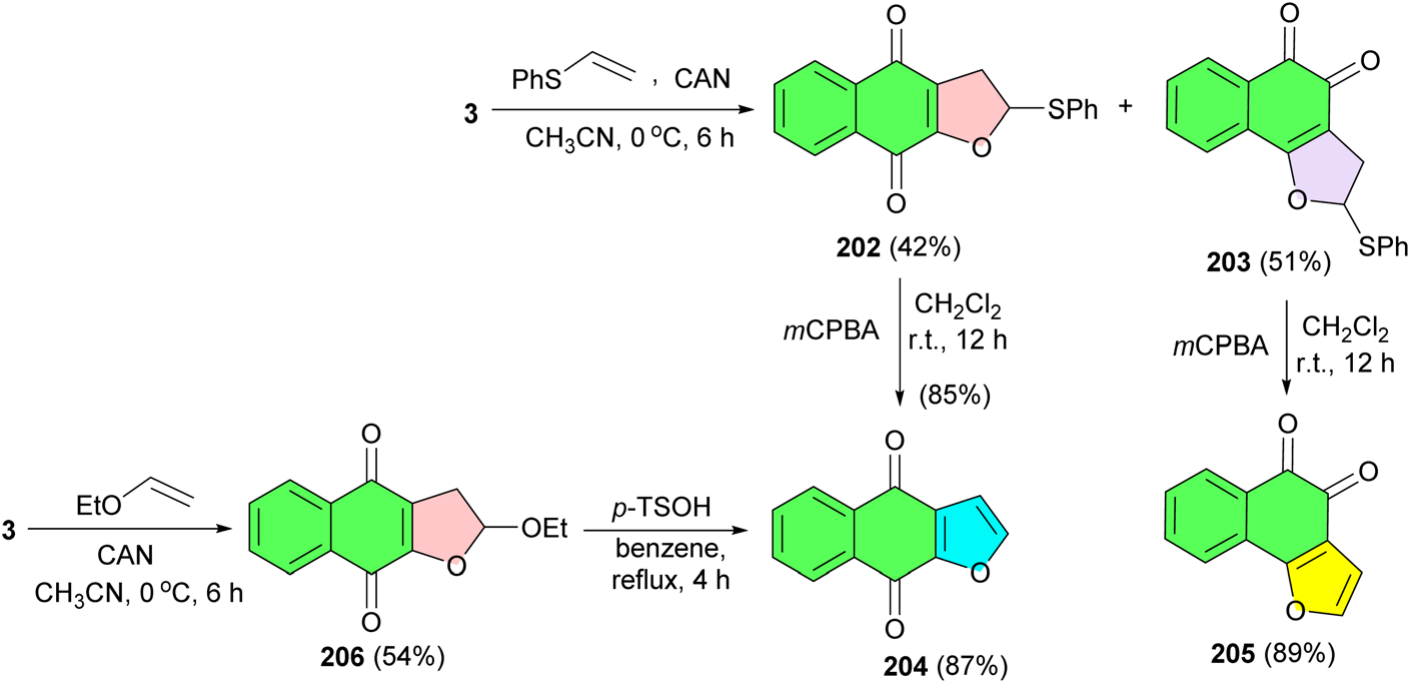
CAN-mediated synthesis of furonaphthoquinones 202–205.

CAN-mediated cycloaddition reaction of lawsone (3) with phenyl vinyl sulfide at 0 °C for 6 h in acetonitrile afforded the dihydrofuronaphthoquinone 202 (42% yield) and 203 (51% yield) as a mixture of linear and angular isomers. The mixture was easily separated by column chromatography and the two isomers were assigned by their spectroscopic data. The conversion of compound 202 to the natural product was begun by elimination of the phenyl sulfide group. Treatment of 202 and 203 with *m*-CPBA in CH_2_Cl_2_ at room temperature for 12 h resulted in avicequinone-B (204) (85% yield) and furonaphthoquinone 205 (82% yield), respectively. Treatment of 3 with ethyl vinyl ether in the presence of CAN at 0 °C for 6 h in acetonitrile gave dihydrofuronaphthoquinone 206 (54% yield) as a single compound, without the isolation required of the expected angular regioisomer. The dihydrofuronaphthoquinone 206 was easily converted to 204 in 87% yield by treatment of *p*-TsOH in refluxing benzene (Scheme 51).^84^

**Scheme 52:**
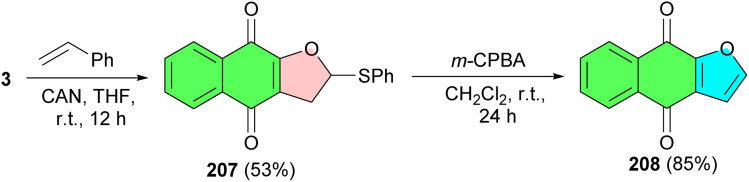
Ceric ammonium nitrate(CAN)-mediated synthesis of furonaphthoquinone 207–208.

Next, Lee and co-workers described synthesis of dihydrofuronaphthoquinone 207 in 53% yield from lawsone and phenyl vinyl sulfide in the presence of CAN at room temperature for 12 h in THF, without formation of any other possible regioisomers. Treatment of 207 with *m*-CPBA in CH_2_Cl_2_ at room temperature for 24 h afforded avicequinone-B 208 in 85% yield (Scheme 52).^85^ It has been shown to have a great cancer chemopreventive activity against Epstein–Barr virus early antigen (EBV-EA) activation, without showing any cytotoxicity.^86^

**Scheme 53:**
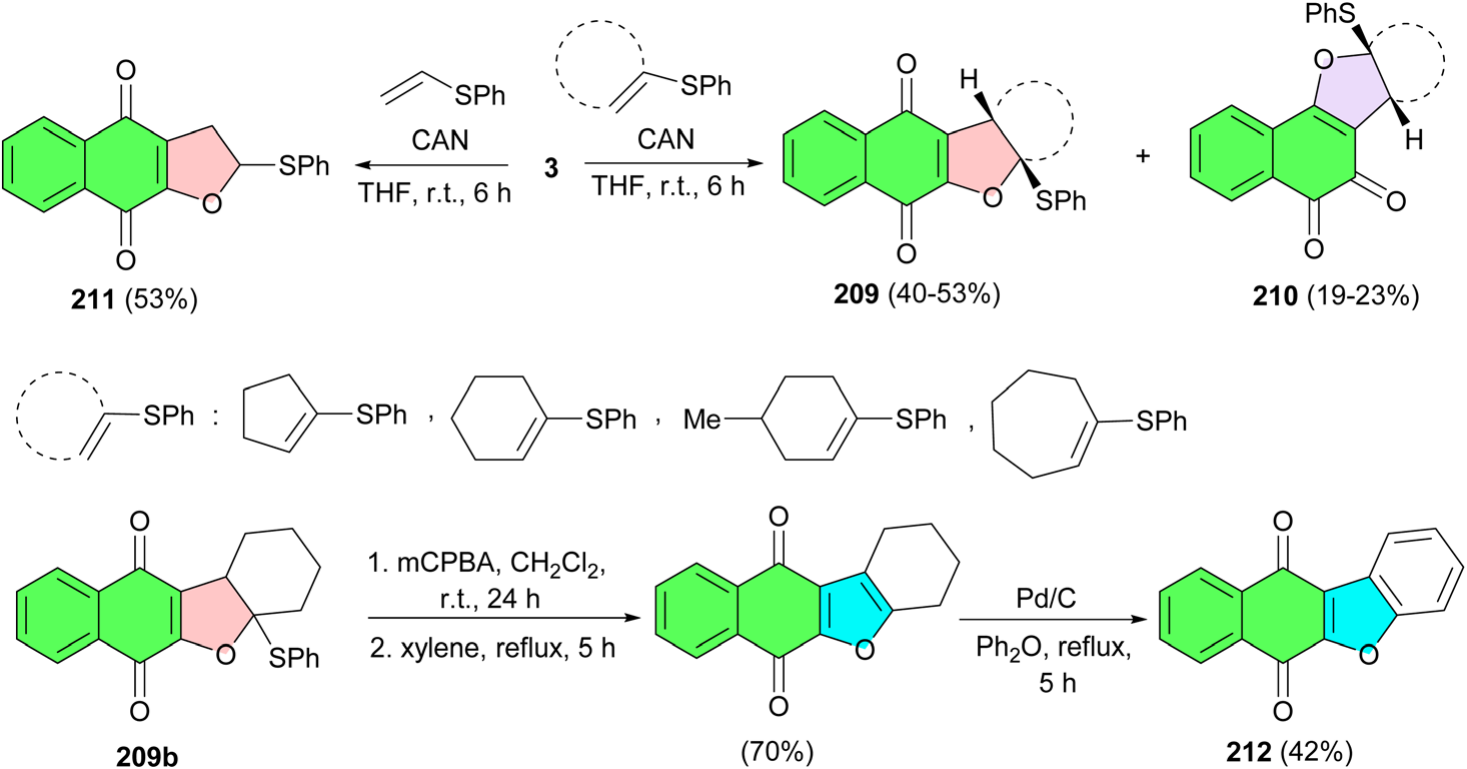
CAN-Mediated synthesis of furanonaphthoquinones 209–212.

The reaction of 2-hydroxy-1,4-naphthoquinone (3) with vinyl sulfides in the presence of ceric(iv) ammonium nitrate in THF at room temperature for 6 h resulted in dihydrofuronaphthoquinone 209 (40–53%) and 210 (19–23%) as a mixture of linear and angular regioisomers. The products were easily purified by column chromatography and the structures of the two isomers were determined by their spectroscopic data. However, reaction with vinyl sulfide at room temperature in THF afforded solely dihydrofuronaphthoquinone 211 in 53% yield, without formation of the other possible regioisomer. Dihydrofuran 209b can be readily converted to benzofuronaphthoquinone derivative 212 which has been reported to have significant biological activities such as antipruritic, antitumor, topo II-mediate DNA cleavage (Scheme 53).^87^

**Scheme 54:**
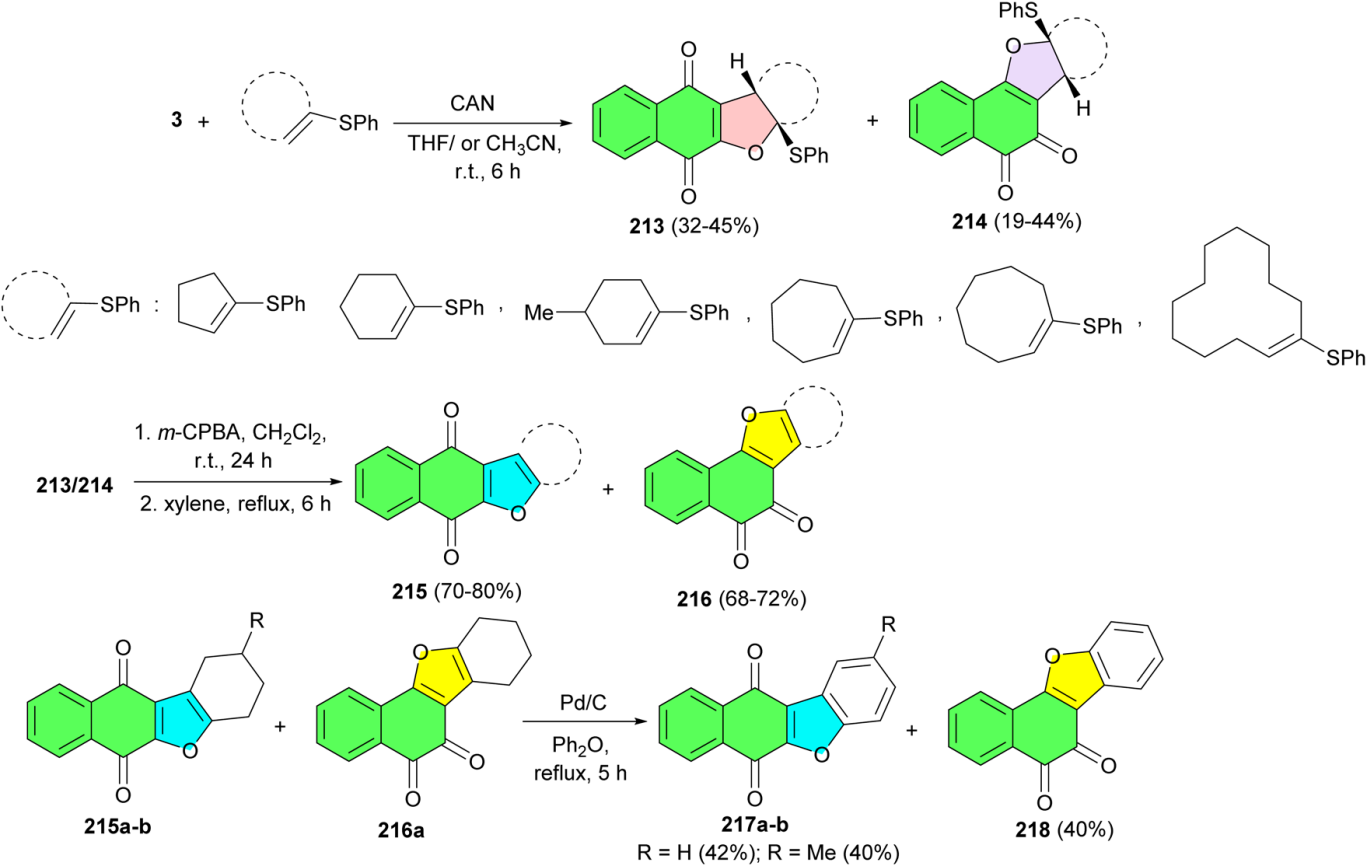
CAN mediated synthesis of furanonaphthoquinones 213–218.

An efficient synthesis of linear and angular dihydrofuranonaphthoquinones 213 and 214 in 19–45% yields, has been carried out starting from 2-hydroxy-1,4-naphthoquinone and a variety of vinyl sulfides using CAN in THF or CH_3_CN at room temperature for 6 h. Also, furanonaphthoquinones 215 and 216 were synthesized with yields of 68–80% by reacting dihydrofuranonaphthoquinones with *m*-CPBA in CH_2_Cl_2_ at room temperature for 24 hours, followed by refluxing in *m*-xylene for 6 h. Moreover, the reaction of furanonaphthoquinones with Pd/C in diphenyl ether under reflux conditions for 5 h afforded benzonaphthoquinones 217a–b and 218 in 40–42% yields (Scheme 54).^88^

**Scheme 55:**
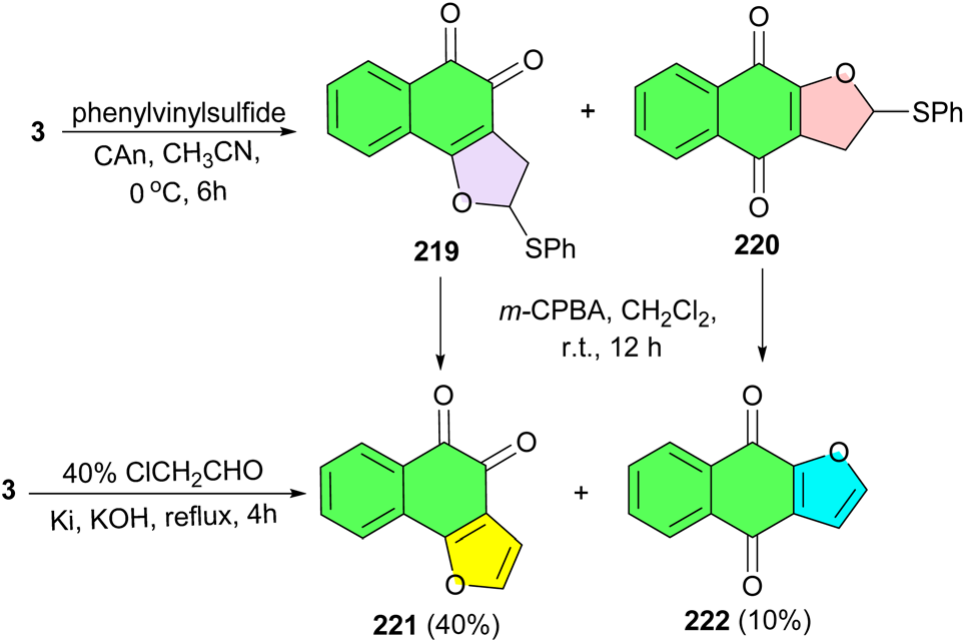
Preparation of furonaphthoquinones 219–222.

In 2009, Cheng and co-workers disclosed synthesis of angular dihydrofuronaphthoquinone 219 and its linear isomer 220 by the reaction of lawsone with phenylvinylsulfide in the presence of ceric ammonium nitrate (CAN) in CH_3_CN at 0 °C for 6 h. Oxidative elimination of 219 and 220 with *m*-CPBA in CH_2_Cl_2_ at room temperature for 12 h afforded angular naphtho[1,2-*b*]furan-4,5-dione (221) and linear naphtho[2,3-*b*]furan-4,9-dione (222). Also, lawsone was reacted with chloroacetaldehyde under reflux conditions for 4 h to give a mixture of 221 and 222 in a yield of 40% and 10%, respectively (Scheme 55).^89^

**Scheme 56:**
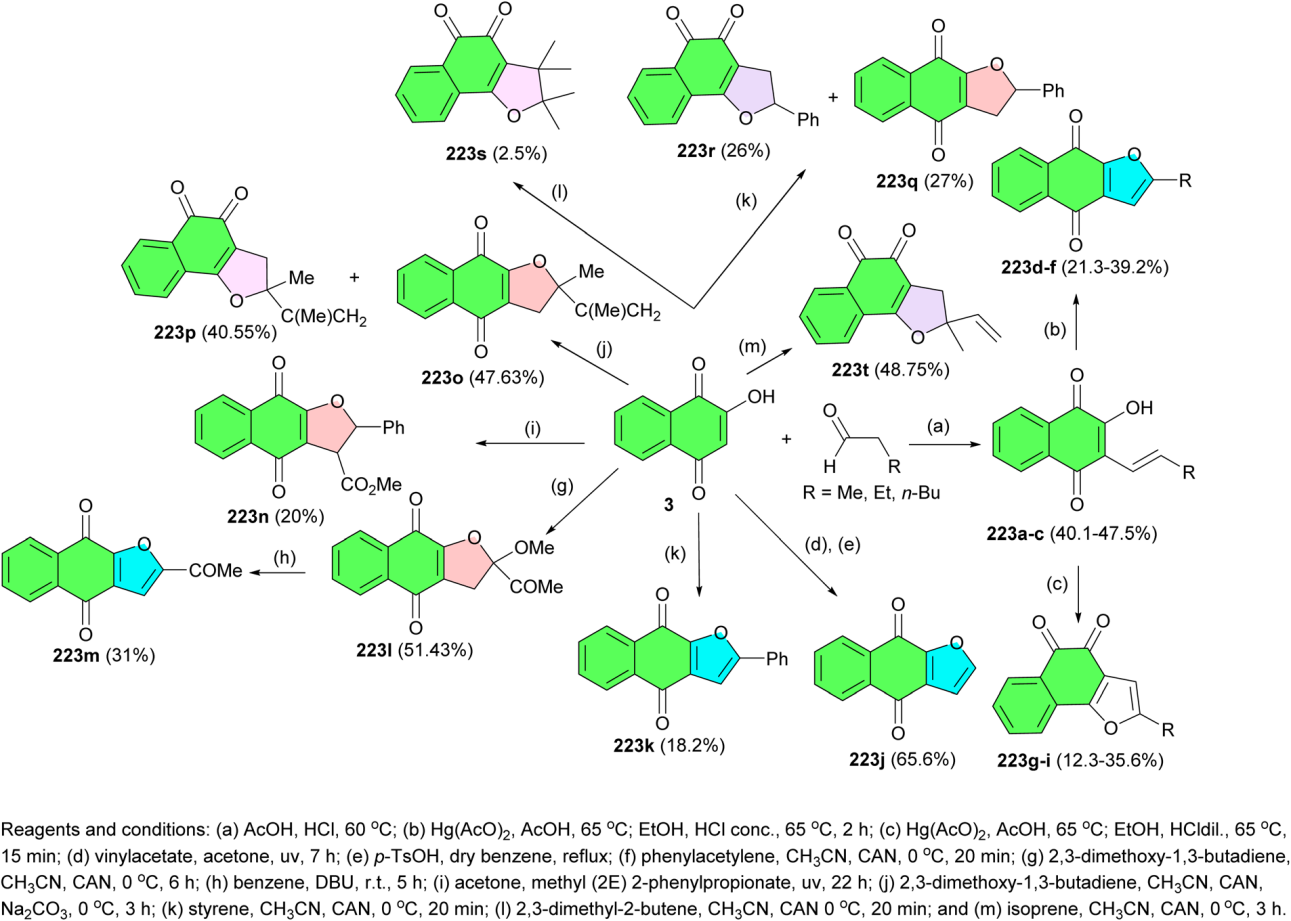
Synthesis of a series of furanonaphthoquinone derivatives 223.

In 2018, the Franco group reported synthesis of a series of furanonaphthoquinone derivatives 223 and evaluated their anti-proliferative activity against the human cell line of colorectal cancer HT-29. Several of these compounds exhibited significant anti-proliferative activity. Condensation of lawsone with aldehydes in acidic condition was carried out and afforded compounds 223a, 223b, and 223c. These compounds were oxidized and cyclized by mercuric acetate in harsh reaction conditions, concentrated hydrochloric acid, 65 °C and 2 h of reaction time, to afford 223d, 223e, and 223f, respectively. Alternatively, milder reaction conditions, *i.e.* diluted hydrochloric acid, 65 °C and 15 min of reaction time, were used to obtain 223g, 223h, and 223i. Cycloaddition reactions of lawsone with different reagents were carried out to obtain the compounds 223j–223t. The compound 223m was obtained by the hydrolysis of 223l (Scheme 56).^90^

**Scheme 57:**
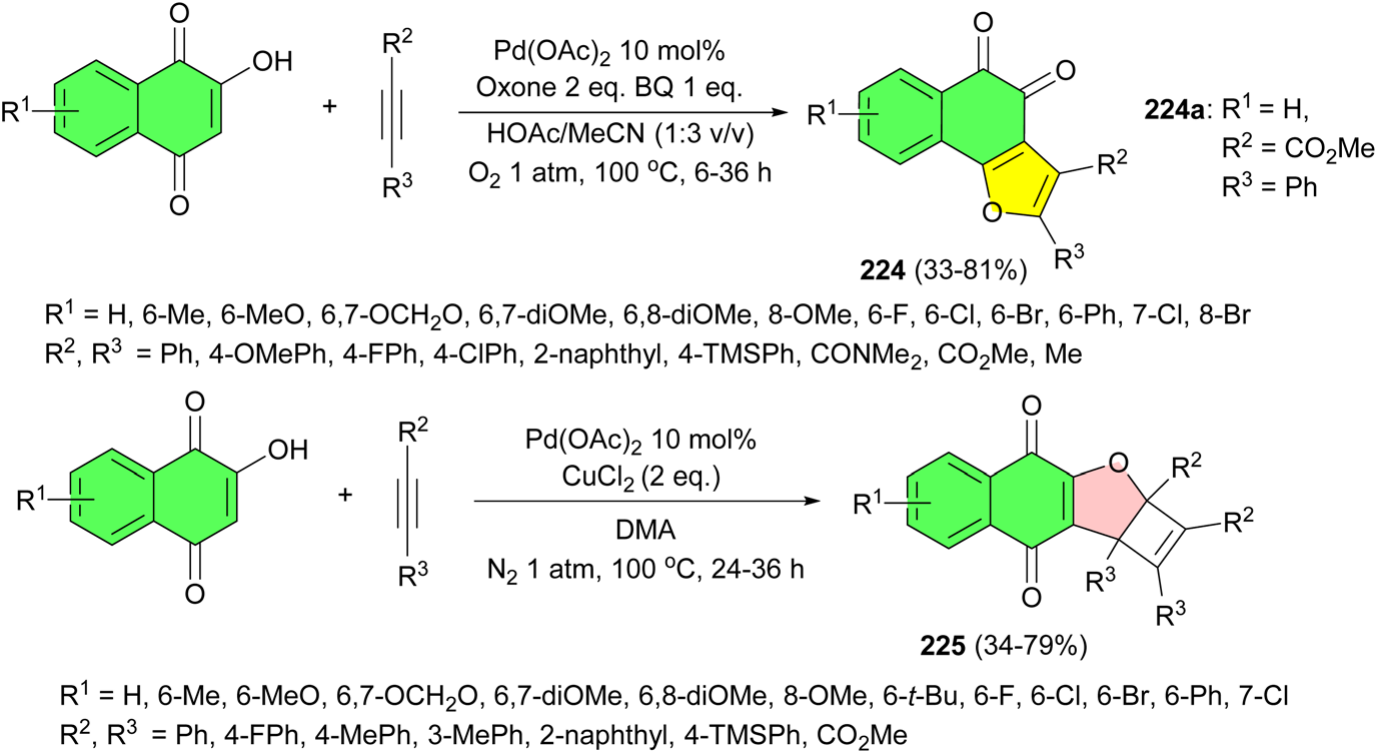
Palladium-catalyzed synthesis of naphthofuroquinones 224–225.

The Wang group developed palladium-catalyzed oxidative switchable annulation of naphthalquinones, bearing electron-neutral, electron-donating, and electron-withdrawing substituents on the aromatic ring, with symmetrical and unsymmetrical alkynes substituted with electron-rich and electron-deficient groups in HOAc/MeCN at 100 °C to assemble a series of biologically relevant functionalized 1,2-naphthofuroquinones 224 in 33–81% yields after 6–36 h and densely functionalized cyclobutene embedded 1,4-naphthofuroquinones 225 in the presence of CuCl_2_ in DMA at 100 °C for 24–36 h in 34–79% yields. Also, the bioactivity of the synthesized compounds was evaluated. The results showed that 224a exhibited a strong endothelial protective effect against oxidized low-density lipoprotein (ox-LDL)-induced human umbilical vein endothelial cell (HUVEC) injury. Additionally, it has no effects on the normal cells (Scheme 57).^91^

**Scheme 58:**
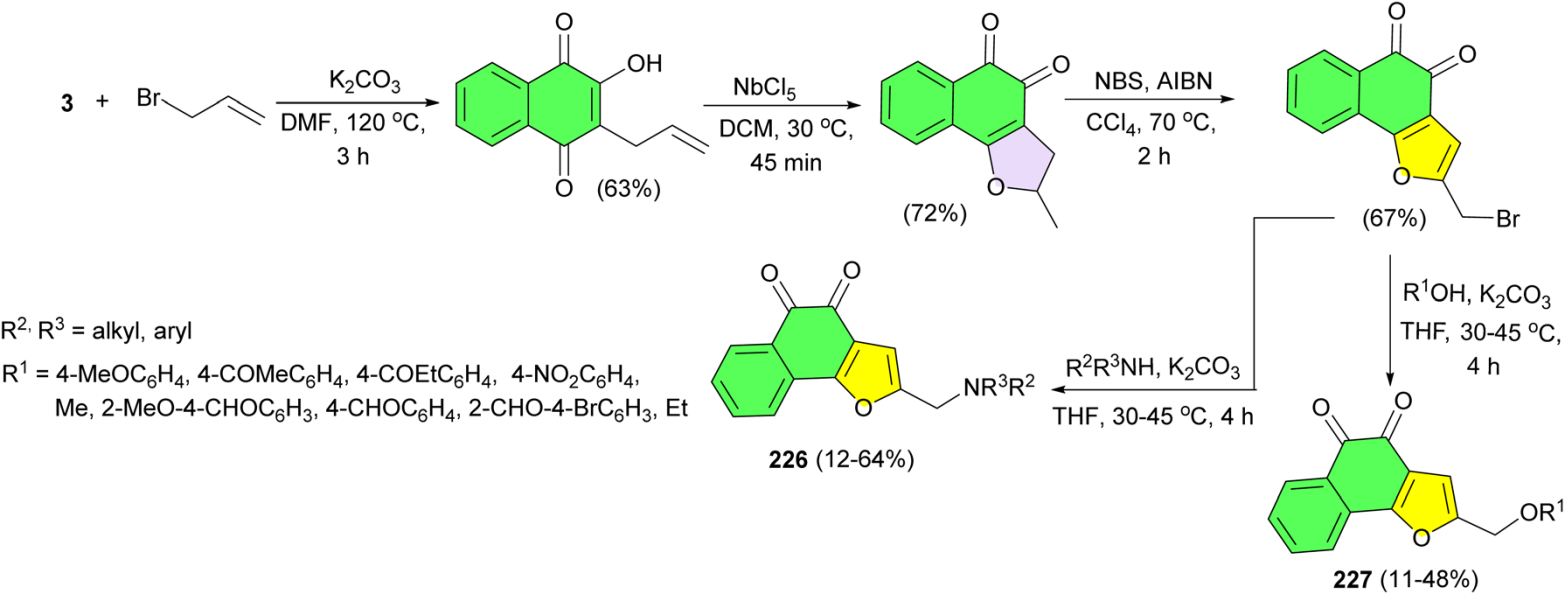
Synthesized L-shaped *ortho*-quinone analogs 226–227.

After that, the Chen group designed and synthesized L-shaped *ortho*-quinone analogs 226–227 using a one pot double-radical synthetic strategy followed by removing methyl at C-3 of the furan ring and introducing a diverse side chain at C-2 of the furan ring. Scheme 58 refers to a step-by-step diagram outlining the synthetic pathway used to produce the compounds. Moreover, the synthesized compounds exhibit cytotoxic activity against human leukemia cells K562, prostate cancer cells PC3, and melanoma cells WM9.^92^

**Scheme 59:**
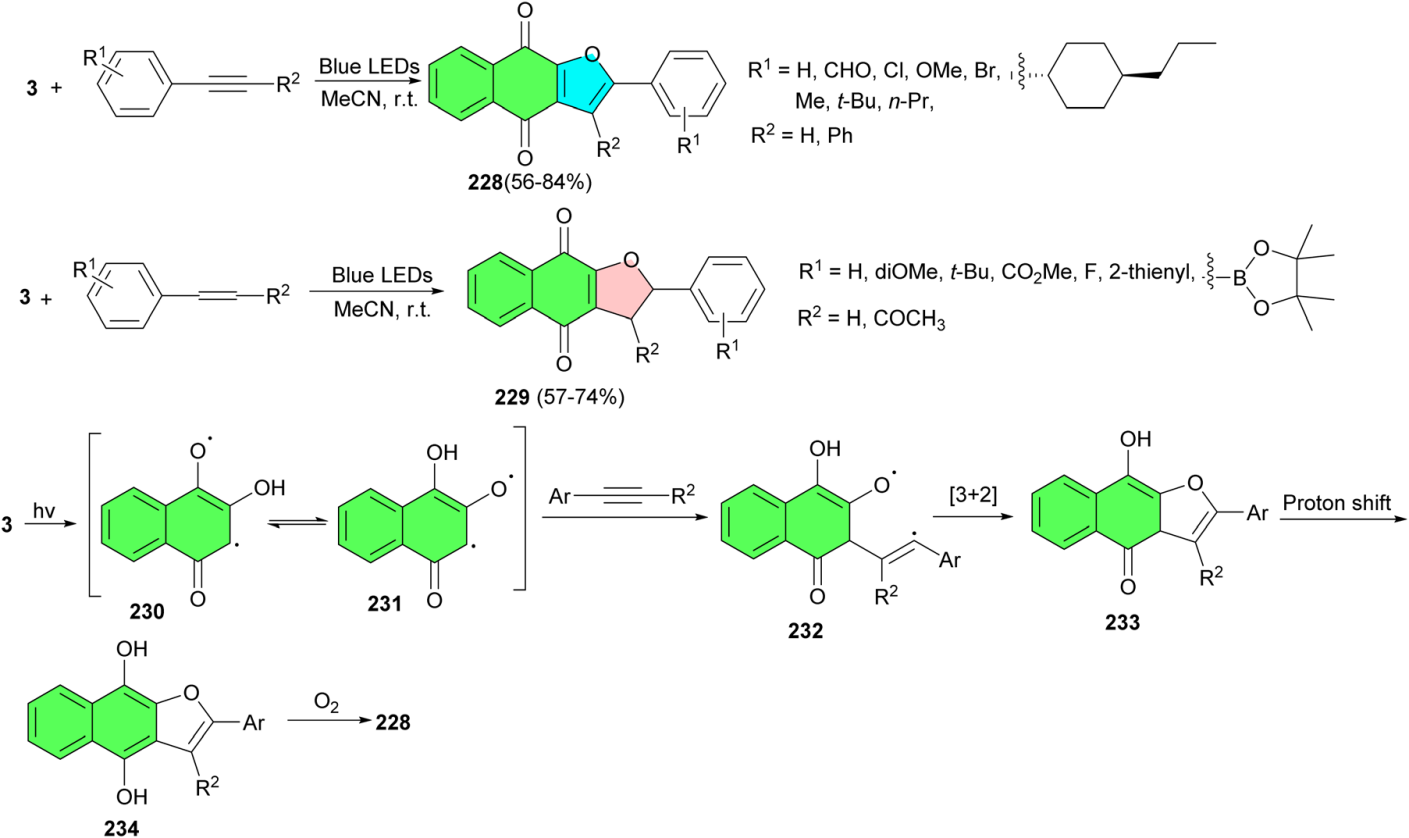
Visible-light-mediated synthesis of naphtho[2,3-*b*]furan-4,9-diones 228–229.

In 2023, Tan and co-workers developed visible-light-mediated [3 + 2] cycloaddition reactions of 2-hydroxy-1,4-naphthoquinones and alkynes and alkenes under irradiation of blue LEDs (460 nm) in the absence of any bases, metals, ligands, or other catalysts in CH_3_CN at room temperature. Under environmentally friendly conditions, a variety of naphtho[2,3-*b*]furan-4,9-diones 228 and dihydronaphtho[2,3-*b*]furan-4,9-diones 229 were delivered within 6 h in 56–84% yields. A plausible mechanism is demonstrated in Scheme 59. First, the irradiation of lawsone in MeCN generates tautomeric excited triplets 230 and 231, which react with an alkyne to give a 1,5-biradical intermediate 232. Subsequently, an intramolecular [3 + 2] cyclization of the intermediate 232 gives hydroquinone intermediate 233. Upon 1,3-hydrogen transfer, the hydroquinone intermediate 234 is formed, and then, naphtho[2,3-*b*]furan-4,9-diones 228 is produced by air oxidation of the hydroquinone by oxygen in the air. Similarly, the [3 + 2] cycloaddition reaction of lawsone with alkenes leading to product dihydronaphtho[2,3-*b*]furan-4,9-diones 229 may proceed in a manner parallel to the [3 + 2] cycloaddition of alkynes and may also involve biradical intermediates.^93^

## 5. Conclusions

Furonaphthoquinone and their dihydro derivatives are a highly important heterocyclic compounds and a well-known pharmacophoric unit present in natural products, drugs, and drug candidates. A great number of naphthofuroquinones have exhibited diverse biological activities. Over the past decades, numerous furonaphthoquinone compounds have been synthesized and studied by researchers, revealing promising biological activities. This review highlights recent strategies for synthesizing various furonaphthoquinones and their dihydro derivatives, along with the exploration of their biological activities. Primarily starting with 2-hydroxy-1,4-naphthoquinones, a variety of synthetic methods have been developed, including multicomponent reactions, CAN-mediated oxidative cycloaddition, photoaddition, thermal cyclization, coupling with olefins or alkynes, one-step cascade approaches involving lawsone, isocyanides, and aldehydes, the Wittig reaction, [3 + 2] cycloaddition, oxidative cyclization/isomerization, Friedel–Crafts acylation/oxidation, nitrogen ylide coupling reactions, and bromine-mediated intramolecular cyclization under diverse catalytic systems and reaction conditions. The synthesized compounds exhibited diverse biological activities such as anti-tumor, anti-plasmodial, anti-parasitic, anti-bacterial, anti-infectious, anti-oxidant, anti-inflammatory, anti-fungal, anti-cancer, anti-pruritic, anti-leukemia, anti-DNA-polymerase, anti-*Trypanosoma cruzi* and anti-epimastigote. We hope this review highlights the versatility of these compounds and inspires current and future generations of chemists to further explore and advance this field.

## Data availability

No new data were generated or analysed for this article.

## Author contributions

All authors contributed to the scientific writing of the review article. Each author has reviewed and approved the final version for submission.

## Conflicts of interest

We have no conflicts of interest to disclose.
